# Mitochondrial bioenergetics and cardiolipin remodeling abnormalities in mitochondrial trifunctional protein deficiency

**DOI:** 10.1172/jci.insight.176887

**Published:** 2024-09-10

**Authors:** Eduardo Vieira Neto, Meicheng Wang, Austin J. Szuminsky, Lethicia Ferraro, Erik Koppes, Yudong Wang, Clinton Van’t Land, Al-Walid Mohsen, Geancarlo Zanatta, Areeg H. El-Gharbawy, Tamil S. Anthonymuthu, Yulia Y. Tyurina, Vladimir A. Tyurin, Valerian Kagan, Hülya Bayır, Jerry Vockley

**Affiliations:** 1 Genetic and Genomic Medicine Division, Department of Pediatrics, UPMC Children’s Hospital of Pittsburgh,; 2 Children’s Neuroscience Institute, Department of Pediatrics, School of Medicine, and; 3 Department of Biological Sciences, Kenneth P. Dietrich School of Arts and Sciences, University of Pittsburgh, Pittsburgh, Pennsylvania, USA.; 4 School of Medicine and; 5 Department of Biophysics, Federal University of Rio Grande do Sul, Porto Alegre, Brazil.; 6 Division of Medical Genetics, Department of Pediatrics, Duke University Medical Center, Durham, North Carolina, USA.; 7 Adeptrix Corporation, Beverly, Massachusetts, USA.; 8 Department of Environmental and Occupational Health, Center for Free Radical and Antioxidant Health, School of Public Health;; 9 Department of Pharmacology and Chemical Biology, School of Medicine; Department of Chemistry, Kenneth P. Dietrich School of Arts and Sciences; and Department of Radiation Oncology, University of Pittsburgh, Pittsburgh, Pennsylvania, USA.; 10 Division of Critical Care and Hospital Medicine, Department of Pediatrics, Redox Health Center, Vagelos College of Physicians and Surgeons, Columbia University Irving Medical Center, New York, New York, USA.; 11 Department of Human Genetics, School of Public Health, Center for Rare Disease Therapy, UPMC Children’s Hospital of Pittsburgh, University of Pittsburgh, Pittsburgh, Pennsylvania, USA.

**Keywords:** Genetics, Metabolism, Fatty acid oxidation, Intermediary metabolism, Mitochondria

## Abstract

Mitochondrial trifunctional protein (TFP) deficiency is an inherited metabolic disorder leading to a block in long-chain fatty acid β-oxidation. Mutations in *HADHA* and *HADHB*, which encode the TFP α and β subunits, respectively, usually result in combined TFP deficiency. A single common mutation, *HADHA* c.1528G>C (p.E510Q), leads to isolated 3-hydroxyacyl-CoA dehydrogenase deficiency. TFP also catalyzes a step in the remodeling of cardiolipin (CL), a phospholipid critical to mitochondrial membrane stability and function. We explored the effect of mutations in TFP subunits on CL and other phospholipid content and composition and the consequences of these changes on mitochondrial bioenergetics in patient-derived fibroblasts. Abnormalities in these parameters varied extensively among different fibroblasts, and some cells were able to maintain basal oxygen consumption rates similar to controls. Although CL reduction was universally identified, a simultaneous increase in monolysocardiolipins was discrepant among cells. A similar profile was seen in liver mitochondria isolates from a TFP-deficient mouse model. Response to new potential drugs targeting CL metabolism might be dependent on patient genotype.

## Abstract 2

Cardiolipin remodeling is deregulated in trifunctional protein deficiency leading to a reduction in mitochondrial oxygen consumption rate that contributes to the pathophysiology of this disease.

## Graphical abstract

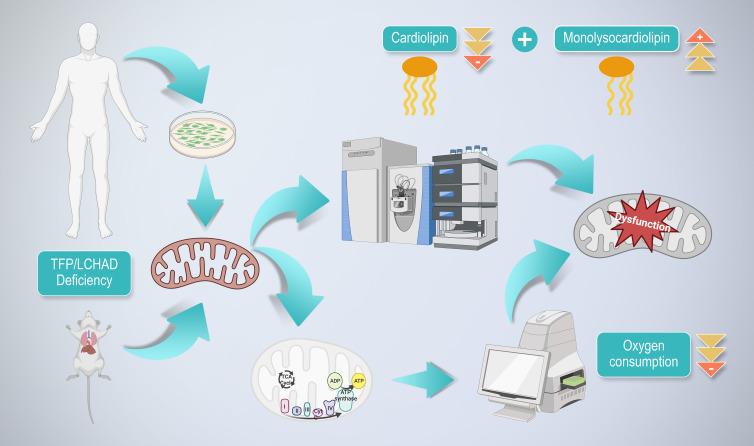

## Introduction

Mitochondrial fatty acid β-oxidation (FAO) is the process by which stored fats are utilized for energy production during times of physiologic stress such as fasting, exercise, and intercurrent illnesses (1). FAO is a major energy source especially for the myocardium, skeletal muscle, and liver. The introduction of tandem mass spectrometry (MS/MS) in newborn screening made it possible to detect genetic deficiencies of FAO by analysis of acylcarnitine profiles (2). Formerly considered rare, FAO disorders (FAODs) were subsequently found to be among the most prevalent conditions identified by newborn screening, affecting 1:9,000 live-born infants (~400 yearly in the United States) (3, 4). Presently, all 50 states include FAODs in their newborn screening programs (5).

Catabolism of long-chain fatty acids involves import into mitochondria by the carnitine palmitoyl transport system, then removal of the successive 2 carbon units (acetyl-CoA) in a 4-reaction cycle (6, 7). The first reaction of the cycle for substrates of greater than 12 carbons is catalyzed by very-long-chain acyl-CoA dehydrogenase (VLCAD), while the mitochondrial trifunctional protein (TFP) performs the next 3 reactions. TFP is a heterotetramer composed of 2 α subunits and 2 β subunits, encoded by the *HADHA* and *HADHB* genes, respectively (8, 9). The HADHA protein contains the long-chain enoyl-CoA hydratase and long-chain 3-hydroxyacyl-CoA dehydrogenase (LCHAD) activities, while the long-chain 3-ketoacyl-CoA thiolase (LKAT) activity is located on the HADHB subunit (9, 10). Most mutations in *HADHA* and all in *HADHB* result in reduced activity of both α and β subunit activities, leading to generalized TFP deficiency phenotype (MIM: 609015). However, a recurrent c.1528G>C (p.E510Q) missense mutation in *HADHA* accounts for about 87% of the alleles reported in isolated LCHAD deficiency (MIM: 609016) (11–13).

In addition to its role in FAO, the TFP α subunit has been shown to exhibit a monolysocardiolipin acyltransferase (MLCLAT) activity, establishing an unexpected link between mitochondrial FAO and cardiolipin (CL) remodeling (14). MLCLAT-1 has been identified as a splice variant of *HADHA*, lacking the first 227 amino acids of the TFP α subunit (15). Despite recent strides in understanding the molecular mechanisms surrounding the role of integral αTFP or a fragment thereof (MLCLAT-1) in CL maturation (14–16), how mutations in *HADHA* and *HADHB* affect this function is little studied. CL is a unique phospholipid that has a conical shape due to its dimeric structure with 4 acyl chains and 2 phosphatidylglycerol moieties that are negatively charged at physiological pH linked to a central glycerol backbone (16–18). This unusual shape promotes the curvature of the inner mitochondrial membrane (IMM), yielding the tubular invaginations called mitochondrial cristae. CL is important for organizing and stabilizing electron transport chain (ETC) supercomplexes, which are quaternary structures of the individual ETC enzyme complexes assembled within the folds of mitochondrial cristae. Following its initial synthesis, nascent CL is deacylated, forming monolysocardiolipin (MLCL), then reacylated with different acyl chains, a process called remodeling. It is believed that tafazzin, the protein product of the *TAFAZZIN* gene, is the major enzyme involved in CL remodeling, acting as a phospholipid-lysophospholipid transacylase that also generates mature CL from MLCL (19). Mature CLs are homoacylated polyunsaturated fatty acid CLs that predominate in high-energy-demanding organs like the heart, skeletal muscles, and liver, contrasting with nascent CLs, which are heteroacylated saturated fatty acid CLs (16). αTFP or its splice variant MLCLAT-1 also plays a role in remodeling CL, transferring a long-chain acyl-CoA moiety to MLCL, generating mature CL (14, 20, 21).

FAODs exhibit a wide variety of clinical phenotypes, degrees of severity, age of onset, organ involvement, and long-term survival, ranging from severe, fatal neonatal to adolescent/adult onset with recurrent rhabdomyolysis (12, 22). The phenotype in most long-chain FAODs includes fasting- and stress-induced hypoketotic hypoglycemia, cardiomyopathy and arrhythmias, skeletal myopathy, and recurrent rhabdomyolysis (3, 5, 6, 12). For unknown reasons, progressive axonal peripheral neuropathy and degenerative retinopathy are unique to TFP and/or isolated LCHAD deficiencies. Unfortunately, symptoms progress even with implementation of standard-of-care therapy following early detection by newborn screening (22, 23).

Peripheral neuropathy appears early in generalized TFP deficiency because of *HADHB* mutations (24). The axonal demyelination found in the affected patients has been attributed to the accumulation of toxic 3-hydroxyacylcarnitines secreted by Schwann cells (25, 26), though its etiology remains largely unsettled. Isolated LCHAD deficiency due to the common mutation in *HADHA*, c.1528G>C (p.E510Q), is associated with a pigmentary retinopathy that can lead to vision loss and later onset neuropathy (5). It has been hypothesized that high levels of 3-hydroxyacylcarnitines found in patients bearing the p.E510Q mutation (much higher than generalized TFP deficiency due to other mutations) leads to retinal damage (5). Of note, both findings are reminiscent of those in some patients with deficiencies of oxidative phosphorylation (OXPHOS), though importantly they do not appear in Barth syndrome due to mutations in the *TAFAZZIN* gene (27). It has previously been demonstrated that TFP α subunit physically interacts with the matrix arm of complex I and that mutations in FAO genes can impact the stability of the FAO-OXPHOS energy complex (28, 29). Alterations in CL content and composition as a result of MLCLAT-1 deficiency could negatively impact the activity of the supercomplexes, thus further compromising mitochondrial bioenergetics and function in peripheral axons and retina. These observations highlight the need to consider a secondary effect on OXPHOS function as an explanation of the unique neurologic and retinal symptoms seen in TFP/LCHAD deficiency.

Although the correlation of genotype with the severity of clinical phenotype, organ involvement, and survival beyond infancy has been extensively investigated, no clear in vitro molecular parameters have been defined that predict outcomes or explain the molecular mechanisms for the unique appearance of peripheral neuropathy and retinopathy in TFP/LCHAD deficiencies (3, 10, 22, 30, 31). Moreover, the impact of *HADHA* and *HADHB* mutations found in patients with TFP/LCHAD deficiency on CL content or structure has only been cursorily investigated in isolated LCHAD deficiency due to the *HADHA* c.1528G>C (p.E510Q) mutation (32).

In this study, we utilize a lipidomics approach with functional analyses of patient-derived fibroblasts to examine genetic heterogeneity and its correlation to phenotype in TFP/LCHAD deficiency and additionally investigate liver mitochondria CL profile of a TFP-deficient mouse model.

## Results

### Genotype confirmation

Biallelic mutations in *HADHA* or *HADHB* identified through clinical testing were confirmed in all patients. Initial PCR amplification was successful for all regions of interest, except exons 14 and 15 of *HADHB* for cell line FB854 (Supplemental Figure 1A; supplemental material available online with this article; https://doi.org/10.1172/jci.insight.176887DS1). *HADHB* exon 15 was amplified with the extra MgCl_2_ protocol of QIAGEN Taq DNA polymerase (Supplemental Figure 1B). *HADHB* exon 14 was successfully amplified with high-fidelity Invitrogen AccuPrime *Pfx* DNA Polymerase (Supplemental Figure 1C). Sanger sequencing confirmed 3 truncating variants: 2 *HADHA* canonical splice site variants (Supplemental Figure 2, A and B) and a single-base *HADHB* deletion leading to a frameshift and premature termination of translation (Supplemental Figure 2C). All truncation variants were found in a compound-heterozygous state with missense variants (Supplemental Table 2 and Supplemental Figure 3, A and C). *HADHB* compound-heterozygous missense variants in cell line FB854 (Supplemental Figure 3, D and E) and the homozygous *HADHA* LCHAD common variant, c.1528G>C (p.E510Q), in cell lines FB822 (Supplemental Figure 3F), FB944, and FB942 were also confirmed.

### Structure analysis and in silico predictive tools for variant pathogenicity assessment

#### αTFP p.E510Q.

**Figure 1:**
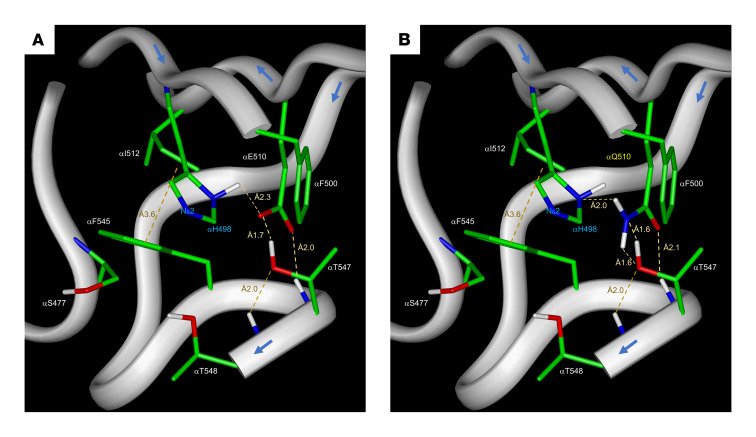
Ribbon and stick representation of part of the TFP-LCHAD active site highlighting residues predicted to play role in catalysis and substrate binding and activation. (**A**) Illustration of the triad αH498-αGlu510-αT547 of the native protein active site where the αGlu510 carboxylate is anchored to the peptide backbone through a hydrogen bond between αT547 backbone amide NH and αGlu510 Oε1. The carboxylate Oε2 is at an interacting distance to αH498 Nε1 hydrogen and the αT547 hydroxyl. (**B**) Illustration of the E510Q mutant protein showing how mutant αGln510 Nε1 can render the αH498 imidazole inert. Atomic coordinates used in this modeling according to Xia et al. (9) 6DV2.pdb. Carbon residues are depicted in green, oxygen in red, nitrogen in blue, and hydrogen in white.

Structural studies and molecular modeling of TFP have previously identified a critical interaction of αE510 and αH498 to form a catalytic dyad that is disrupted in the p.E510Q LCHAD mutation (8, 9). We performed additional analysis of the αE510 carboxylate and found that its configuration is also appropriate for activating the αH498 imidazole ring through direct interaction with αT547, forming an αH498-αE510-αT547 triad (Figure 1A). Substituting a glutamine for the αGlu510 position (Figure 1B) reveals that replacement of the Oε2 with an amino group reverses the role of the Oε2 atom in that position, inactivating the αH498 Nε2 hybridization state, rendering the αH498 residue catalytically inactive, and disrupting the αH498-αE510-αT547 triad function.

#### Other variants.

**Figure 2:**
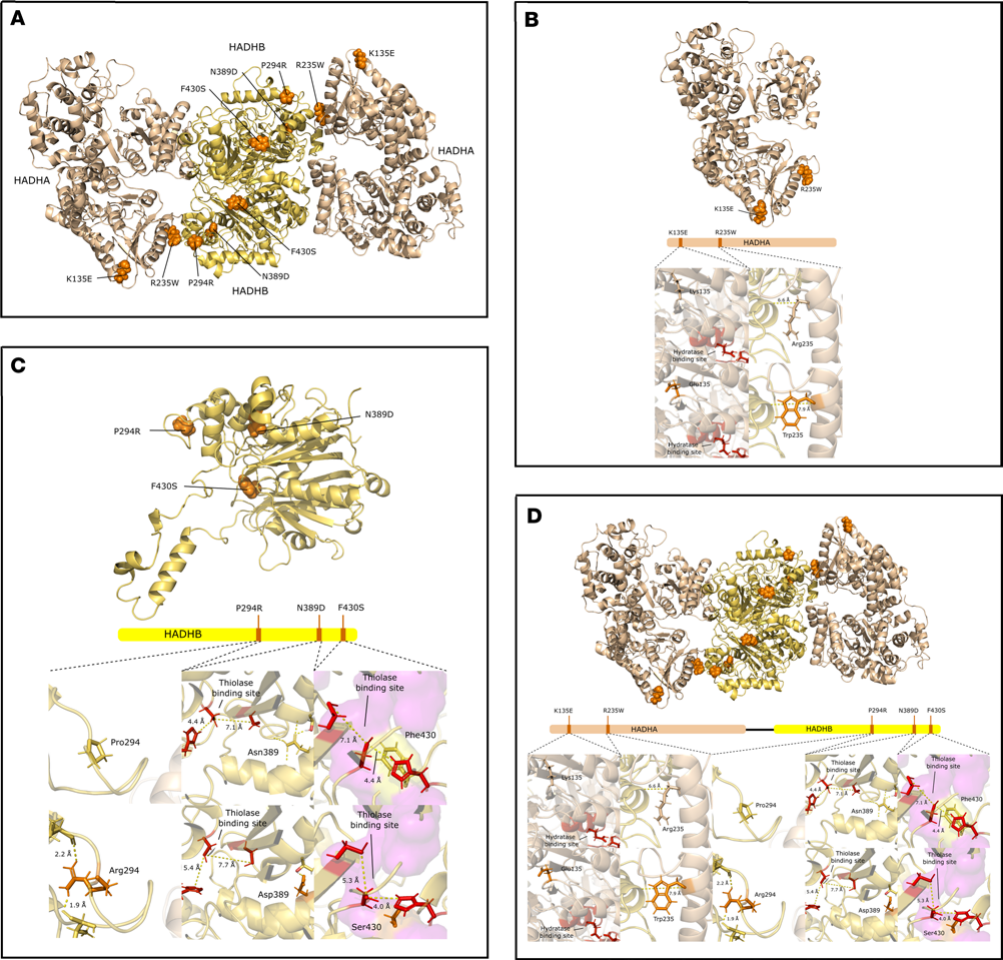
Solid ribbon model of the missense mutation sites in the tetramer structure of the mitochondrial TFP. (**A**) Mutations are identified in orange, α subunits in cream, and β subunits in yellow. Close-up views of the interaction of residues in wild-type (**B**, **C** upper panels), *HADHA* mutants (**B** bottom panels), and *HADHB* mutants (**C** bottom panels). All critical residues are shown as ball and stick, whereas the backbone is shown in cream color cartoon representation; numbering refers to reference sequences retrieved from National Center for Biotechnology Information (NCBI) (73, 74). Catalytic residues depicted as red sticks, mutated residues as orange sticks, wild-type residues as cream sticks, and stabilizing pockets as magenta surfaces. αLys135 (mutated) and αGlu135 (wild-type) both do not interact with the hydratase catalytic site (**B** bottom left). The distance between the α-carbon of βAla233 and α235 increases from 6.6 Å (αArg235) to 7.9 Å (αTrp235) (**B** bottom right). βAsp389 increases the distance between thiolase catalytic site residues βC458, βC138, and βH428 (**C** bottom center). βSer430 interferes with a stabilizing pocket near the catalytic site (**C** bottom right). The loss of proline’s conformational rigidity induced by βArg294 may affect protein tertiary structure (**C** bottom left). Cartoon representation of all 5 missense mutations as solid ribbon models (**D**). Residues represented as in panels **B** and **C**.

Molecular modeling was next used to assess the likely structural effects of the remaining 5 missense mutations identified in our patients (Figure 2A). In αTFP p.R235W, αW235 destroys 2 hydrogen bonds with βA232 (distances 1.9 and 2.1 Å), and an internal α subunit salt bridge with αE238 (1.76 Å), and although it creates a hydrogen bond with βA233 (distance 2.12 Å), the distance between the α carbon of this residue and α235 increases from 6.6 Å (αR235) to 7.9 Å (αW235). Therefore, it is predicted to destabilize α and β subunit interaction (Figure 2B). In contrast, αTFP p.K135E does not interfere with the hydratase catalytic site or disrupt the interaction with the β subunit. Rather, the change in protein folding potentially could be accommodated (Figure 2B). βTFP p.N389D clearly increases the distance between LKAT catalytic site residues βC458, βC138, and βH428, suggesting it indirectly impacts thiolase activity (Figure 2C). βTFP p.F430S interferes with a stabilizing pocket near the catalytic site, likely affecting substrate binding and LKAT function (Figure 2C). The loss of conformational rigidity induced by βTFP p.P294R is predicted to affect tertiary structure, reducing protein stability and LKAT activity (Figure 2C). Figure 2D shows a schematic representation of all 5 missense mutations. Summaries of the assessments of variant pathogenicity by structure analysis and by in silico predictive tools are found in Supplemental Tables 4 and 5.

### TFP protein in patient cell lines

**Figure 3:**
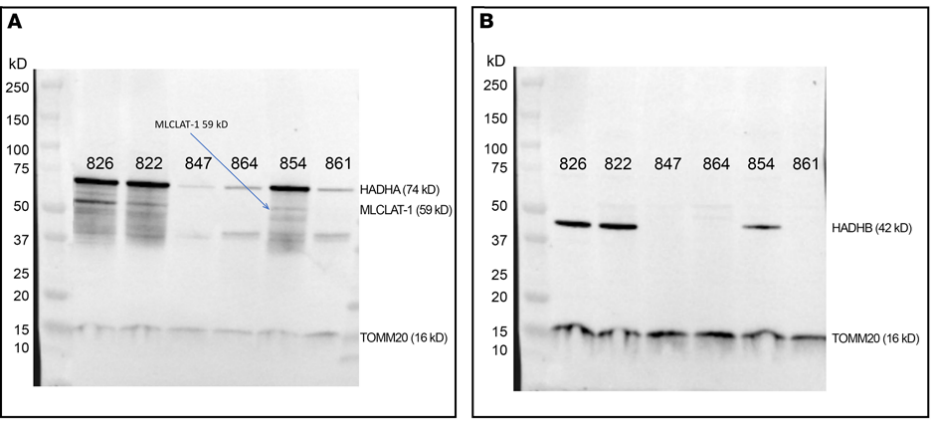
Western blot analysis of whole cell sonicates of cultured fibroblasts. (**A** and **B**) Patient cell lines are numbered as in Supplemental Tables 1 and 2; control fibroblasts: FB826 is from a healthy 40-year-old woman; rabbit monoclonal anti-TOMM20 was used as a positive control. (**A**) The primary antibody was mouse monoclonal anti-HADHA. αTFP and monolysocardiolipin acyltransferase (MLCLAT) protein content are stable in fibroblasts from patients with exclusively missense variant genotypes (FB822, homozygous LCHAD common variant; FB854, compound heterozygous *HADHB* missense variants). The presence of truncating variants in both *HADHA* and *HADHB* decreased the content of αTFP and MLCLAT in various degrees. The 54 kDa and 43 kDa derivatives of αTFP are also visible; their significance is unknown. (**B**) The primary antibody was mouse monoclonal anti-HADHB. βTFP is barely detectable in cells with truncating variants in both *HADHA* and *HADHB*, while this subunit protein is almost identical to controls in cells with exclusively missense variant genotypes (FB822 and FB854).

αTFP and βTFP subunit protein were substantially reduced in patient cells harboring truncating (null) variants, canonical splice site variants in *HADHA* (FB847 and FB864), and a single-base deletion with frameshift in *HADHB* (FB861) (Figure 3, A and B). The 59 kDa band corresponding to MLCLAT-1 was not visible (Figure 3A), and βTFP protein was barely detectable in these patient cells (Figure 3B). Cells homozygous for the common LCHAD variant (FB822) or compound heterozygous for *HADHB* missense variants (FB854) had protein levels comparable to controls.

### Expression of variant mRNA

**Figure 4:**
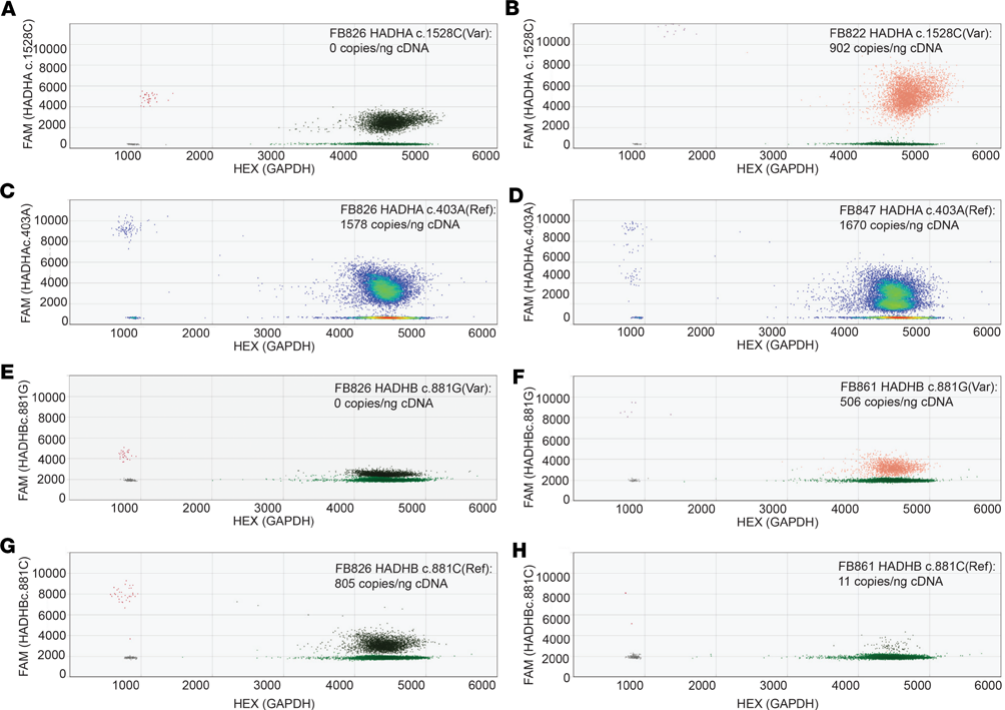
Quantitative expression analysis of *HADHA* and *HADHB* alleles in TFP patient fibroblasts by reverse transcription droplet digital PCR assay. Data are shown as 2-dimensional heatmaps with expression of *HADHA*/*HADHB* alleles on channel 1 vertical axis (6-carboxyfluorescein; FAM) and GAPDH (hexachloro-fluorescein; HEX) on channel 2 horizontal axis. Note that *GAPDH* fluorescence reached near saturation in all samples. Amplitude plots highlighting density in **C** and **D** and colored plots for double-positive droplet concentrations for reference (dark green) and variant (orange) expression in **A**, **B**, and **E**–**H**. Var, variant. (**A** and **B**) Assay assessing *HADHA* c.1528C LCHAD common variant shows a low-amplitude-positive cluster found in FB826 control cDNA that is shifted toward higher amplitude in FB822 cDNA, suggesting that the LCHAD mRNA is expressed. (**C** and **D**) The *HADHA* c.403A reference probe shows a single population of positive droplets in control FB826 but 2 clusters in FB847, indicating that both variant alleles *HADHA* c.2146+1G>A and c.403A>G are expressed. (**E** and **F**) Assay assessing *HADHB* c.881C reference allele expression in FB826 and FB861 reveals a single cluster in FB826 that is greatly diminished in FB861, consistent with degradation of the frameshift allele *HADHB* c.693delC. (**G** and **H**) Assay assessing *HADHB* c.881G variant allele verified that it is found only in FB861. Control FB826 shows a very low channel 1 amplitude cluster.

Although genotypes with truncating mutations invariably led to reduced TFP complex in patient cells, the level of mRNA was variable. In FB847, both the *HADHA* c.403A reference and c.403G mutant allele were expressed at equal levels, indicating that both mutant alleles were transcribed, and the mRNAs were stable (Figure 4, C and D). In contrast, the level of *HADHB* c.881C reference was diminished in FB861 compared with control FB826 (Figure 4, G and H). This finding is consistent with nonsense mediated decay of the *HADHB* c.693delC mRNA. Detection of the FB861 c.881G allele using a specific mutation probe indicated that it was expressed (Figure 4, A and B). The common LCHAD variant c.1528C was expressed as evidenced by the shift to drastically higher amplitude in FB822 cDNA compared with the low-amplitude-positive cluster in control FB826, probably from illegitimate probe annealing (Figure 4, G and H).

### Mitochondrial respiration

**Figure 5:**
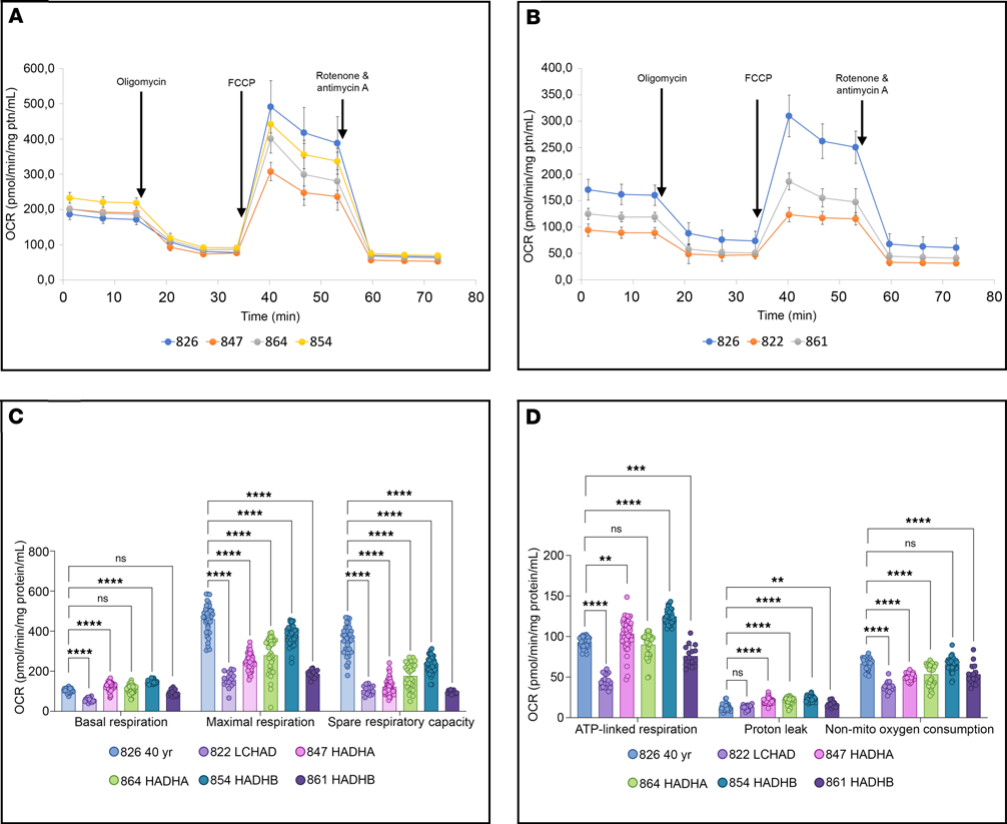
Mitochondrial bioenergetics parameters of TFP/LCHAD-deficient fibroblasts using the Seahorse XF Cell Mito Stress Test kit. Curve points or bars are means ± SD. (**A**) Mitochondrial oxygen consumption rate (OCR) changes in cell lines FB847 (*HADHA*), FB864 (*HADHA*), and FB854 (*HADHB*). ptn, protein; FCCP, carbonyl cyanide-*p*-trifluoromethoxyphenyl-hydrazone. (**B**) Mitochondrial OCR changes in cell lines FB822 (LCHAD common mutation, *HADHA*) and FB861 (*HADHB*). (**C**) Mitochondrial bioenergetics parameters: (a) basal respiration, (b) maximal respiration, (c) spare respiratory capacity. (**D**) Mitochondrial bioenergetics parameters: (a) ATP-linked respiration, (b) proton leak, (c) non-mito O_2_ consumption. Cells were cultured in media with glucose for 24 hours and incubated for 1 hour without glucose before the Seahorse analysis. A clear reduction of maximal respiration and spare respiratory capacity was observed in all TFP/LCHAD-deficient fibroblasts compared with control (**C**). Nevertheless, some cells were able to maintain basal respiration and ATP production, meeting their baseline energy demand, except for FB822 (LCHAD-deficient) and FB861 (*HADHB*) (**C** and **D**). Mitochondrial bioenergetics parameters represent data from 6 Seahorse XF Cell Mito Stress assays, totaling from 16 to 50 biological repeats for each fibroblast cell line. Statistical test: 1-way ANOVA followed by Dunnett’s multiple comparisons test. *P* values: **P ≤ 0.01, ***P ≤ 0.001, ****P ≤ 0.0001, compared with FB826, a control cell line from a 40-year-old woman.

Most of the patient fibroblasts had preserved basal respiration (Figure 5, A and C). ATP-linked respiration, represented by the decrease in OCR following injection of the ATP synthase (complex V) inhibitor oligomycin, was also similarly preserved in almost all cell lines (Figure 5, A and D). In contrast, the isolated LCHAD-deficient cell line (FB822), and the *HADHB* compound-heterozygous cell line (FB861), were particularly impaired in all mitochondrial bioenergetic parameters, including basal respiration and ATP-linked respiration (Figure 5, B–D). There was a clear reduction of maximal respiration and spare respiratory capacity in all patient cell lines when assayed without glucose (Figure 5, A–C).

### FAO flux is reduced in patient cells

**Figure 6:**
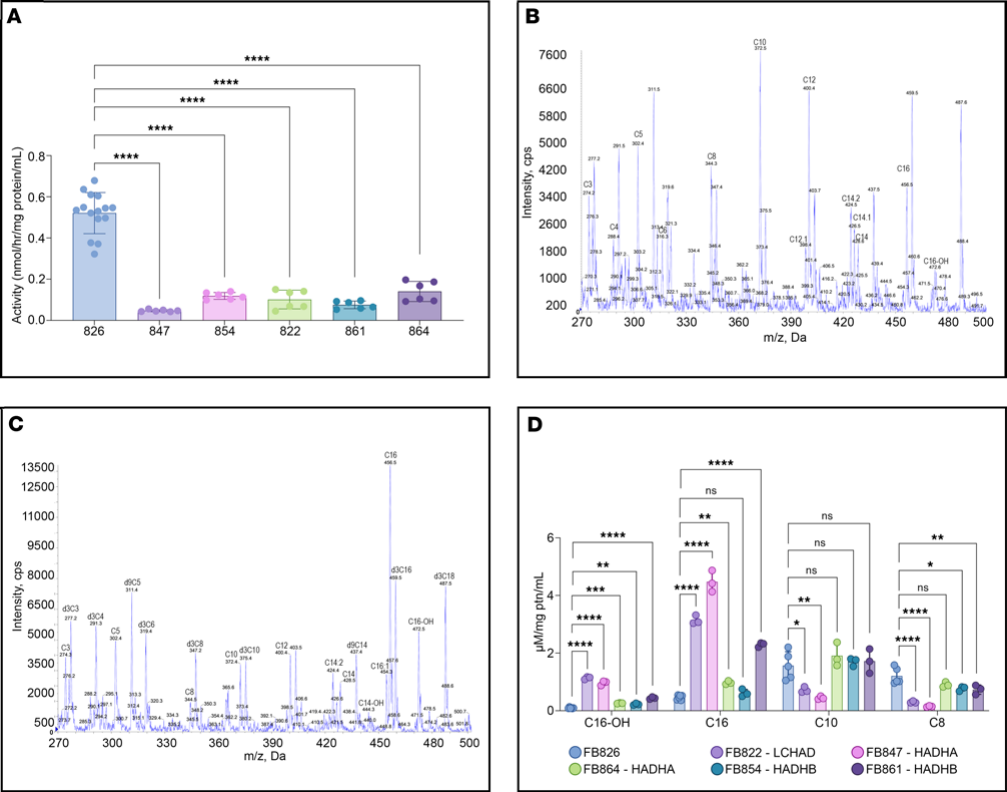
Functional assays of TFP/LCHAD-deficient fibroblasts. Scatterplots with bars show means with SD. (**A**) Fatty acid oxidation (FAO) flux. Cells were grown at 37°C for 24 hours in media containing glucose and then incubated in glucose-free media containing [9,10 ^3^H]-oleate and l-carnitine. FAO flux was measured by monitoring the release of ^3^H_2_O from [9,10 ^3^H]-oleate. The release of ^3^H_2_O was significantly decreased in all patient fibroblasts. Data from 7 assays with 6 and 15 biological repeats of the mutant fibroblasts and control fibroblasts, respectively. Statistical test: ordinary 1-way ANOVA followed by Dunnett’s multiple comparisons test. (**B**) Electrospray ionization–MS/MS (ESI-MS/MS) acylcarnitine profile of fibroblast cell line FB854, compound heterozygous for 2 *HADHB* gene mutations: missense mutations c.1165A>G (p.N389D) and c.1289T>C (p.F430S). cps, counts per second. (**C**) ESI-MS/MS acylcarnitine profile of fibroblast cell line FB822, homozygous for the common LCHAD mutation, p.E510Q. Higher levels of C16-OH were observed. (**D**) Levels of key acylcarnitines in culture media after 72 hours’ overload with unlabeled palmitic acid. Data from 3 to 6 biological repeats of acylcarnitine profiles after 2 distinct challenges with unlabeled palmitic acid. Statistical test: ordinary 1-way ANOVA followed by Dunnett’s multiple comparisons test. *P* values: *P ≤ 0.05, **P ≤ 0.01, ***P ≤ 0.001, ****P ≤ 0.0001, compared with FB826, a control cell line from a 40-year-old woman.

FAO flux as measured by the release of ^3^H_2_O from [9,10-^3^H] oleate upon incubation with cells was significantly decreased in all patient fibroblasts, including those with near-normal levels of TFP protein (FB822 and FB854; Figure 6A).

### Acylcarnitine profiles

Two distinct acylcarnitine profiles were found. The first was found in 2 patient fibroblasts, FB854 (*HADHB*, compound heterozygous) and FB864 (*HADHA*, compound heterozygous). The profile showed low levels of a marker of TFP/LCHAD deficiency, 3-hydroxypalmitoylcarnitine (C16-OH); low levels of palmitoylcarnitine (C16); and high levels of octanoylcarnitine (C8), decanoylcarnitine (C10), and dodecanoylcarnitine (C12) (Figure 6B). This indicates that the fibroblasts were able to catabolize palmitate but stalled at medium-chain acyl-CoA substrates. The other profile in FB822 (LCHAD common mutation homozygous) and FB847 (*HADHA*, compound heterozygous) exhibited high levels of C16-OH and C16 but reduced levels of medium-chain acylcarnitines (Figure 6C). This profile implies a more severe block in long-chain FAO. Figure 6D summarizes the observed acylcarnitine profiles’ quantitative data.

### CLs, MLCLs, dilysocardiolipins, and oxidized cardiolipins are abnormal in mitochondria isolated from patient fibroblasts

**Figure 7:**
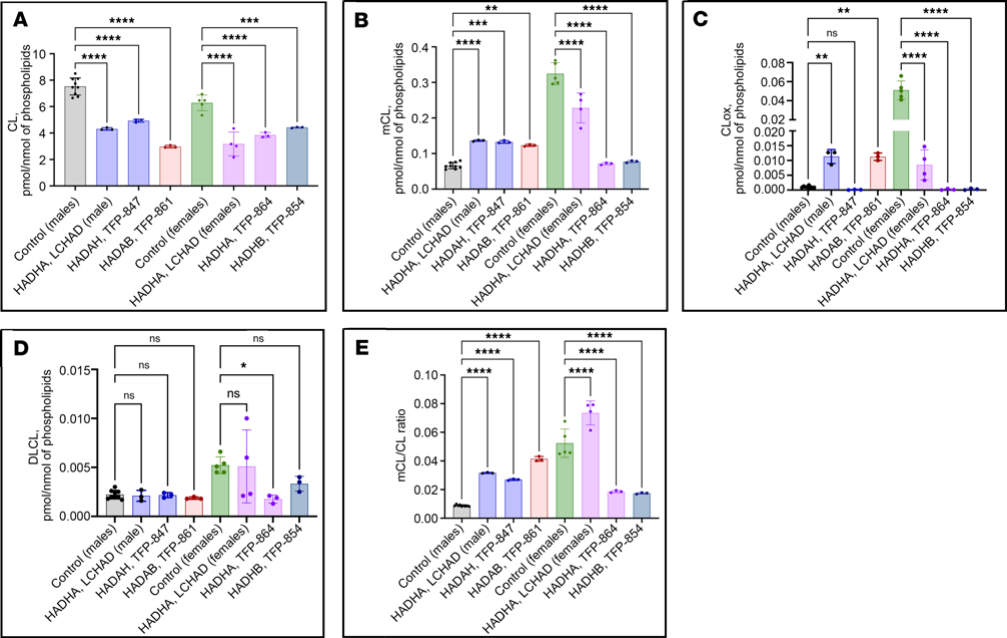
LC-MS/MS assessment of cardiolipins (CL), monolysocardiolipins (mCL), oxidized cardiolipins (CLox), and dilysocardiolipins (DLCL) in mitochondria isolated from TFP/LCHAD-deficient fibroblasts and controls (part I). (**A**) Quantification of CL. (**B**) Quantification of mCL. (**C**) Quantification of CLox. (**D**) Quantification of DLCL. (**E**) Calculated MLCL/CL ratios. The concentration of CL was higher in male and female controls than in TFP/LCHAD-deficient fibroblasts. mCL levels were higher in female than male controls. TFP/LCHAD-deficient fibroblasts from males had higher mCL concentrations, while those from females had lower mCL concentrations than their respective controls. A *HADHA* p.E510Q homozygous (FB822) and a *HADHB* compound heterozygous (FB861) cell line had increased levels of CLox compared with male controls, while all TFP/LCHAD-deficient fibroblasts from females had lower levels of CLox than their controls. DLCLs were decreased in a *HADHA* compound-heterozygous (FB864) cell line compared with female controls. MLCL/CL ratios were increased in all TFP-LCHAD-deficient fibroblasts, except in 2 cell lines from females, FB864 and FB854, *HADHA* and *HADHB* compound-heterozygous fibroblasts, respectively. Scatterplots with bars indicating means of 3 biological repeats with 3 technical repeats for male and female controls and female LCHAD-deficient fibroblasts, for all other cell lines means of 3 technical repeats. Statistical test: ordinary 1-way ANOVA followed by Holm-Šídák multiple comparisons test. SD shown by error bars. *P* values: *P ≤ 0.05, **P ≤ 0.01, ***P ≤ 0.001, ****P ≤ 0.0001, compared with FB826, a control cell line from a 40-year-old woman.

**Figure 8:**
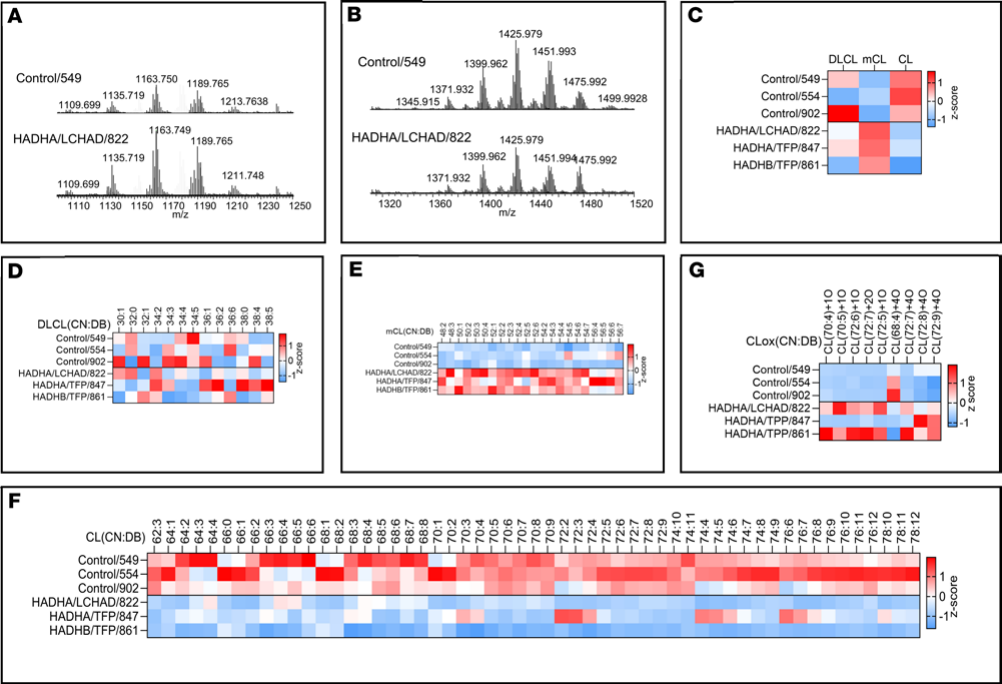
LC-MS/MS assessment of cardiolipins (CL), monolysocardiolipins (mCL), oxidized cardiolipins (CLox), and dilysocardiolipins (DLCL) in mitochondria isolated from male TFP/LCHAD-deficient fibroblasts and controls (part II). Results for patient fibroblasts and controls from females are found in Supplemental Figure 4. Typical mass spectra of mCL (**A**) and CL (**B**) obtained from control (FB549) and TFP/LCHAD-deficient fibroblasts (FB822). TFP/LCHAD mutations induced changes in the total content of CL, mCL, and DLCL (**C**). TFP/LCHAD mutations induced changes in the content of molecular species of DLCL (**D**), mCL (**E**), CL (**F**), and CLox (**G**) in human fibroblasts from males. CN:DB, total carbon number and double bonds of fatty acyl moieties in phospholipid species. Data (pmol/nmol of phospholipids) are presented as heatmaps autoscaled to *Z*-scores: *Z*-score of 0 is marked by white, +3 by dark red, and –3 by dark blue cells, indicating that the sample value is identical to mean value, or 3 SD above or below the mean, respectively. Each lane represents the mean of 3 technical repeats.

**Figure 9:**
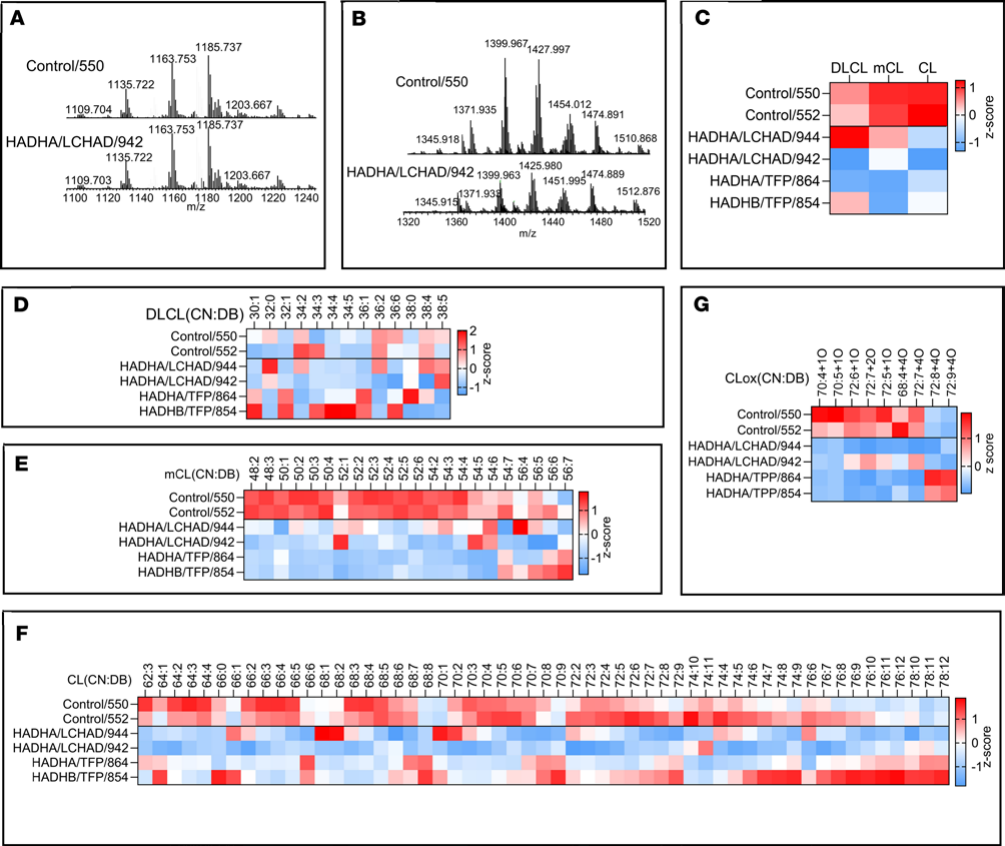
LC-MS/MS assessment of cardiolipins (CL), monolysocardiolipins (mCL), oxidized cardiolipins (CLox), and dilysocardiolipins (DLCL) in mitochondria isolated from female TFP/LCHAD-deficient fibroblasts and controls. Typical mass spectra of mCL (**A**) and CL (**B**) obtained from control (FB550) and TFP/LCHAD-deficient fibroblasts (FB942). TFP/LCHAD mutations induced changes in the total content of CL, mCL, and DLCL (**C**). TFP/LCHAD mutations induced changes in the content of molecular species of DLCL (**D**), mCL (**E**), CL (**F**), and CLox (**G**) in human fibroblasts from females. Data (pmol/nmol of phospholipids) are presented as heatmaps autoscaled to *Z*-scores: *Z*-score of 0 is marked by white, +3 by dark red, and –3 by dark blue cells, indicating that the sample value is identical to mean value, or 3 SD above or below the mean, respectively. Each lane represents the mean of 3 technical repeats.

The concentration of total CLs was higher in unaffected male and female controls than in TFP/LCHAD-deficient fibroblasts (Figure 7A). CLs along the whole spectra of chain length and saturation were reduced in male patient fibroblasts (Figure 8F), and the reverse was found for MLCLs, compared with control fibroblasts from unaffected males (Figure 8E). Reduction of CL species in female patient fibroblasts was also seen along the whole spectra, except for the *HADHB* generalized TFP-deficient cell line, FB854, which showed increased levels of some polyunsaturated and very-long-chain CLs (Figure 9F). Total MLCLs were increased in male patient fibroblasts, but this increase was not found in female patient fibroblasts compared to their respective controls (Figure 7B). Levels of MLCLs of different chain length and saturation were higher in unaffected female controls compared with their male counterpart fibroblasts (Figure 7B; Figure 8, C and E; and Figure 9, C and E). However, MLCL/CL ratios from LCHAD-deficient fibroblasts from 1 male (FB822) and 2 females (FB944 and FB942) were increased compared with their respective controls (Figure 7E). *HADHA* (FB847) and *HADHB* (FB861) generalized TFP-deficient fibroblasts from males also showed increased MLCL/CL ratios.

LCHAD-deficient (FB822) and *HADHB* (FB861) generalized TFP-deficient male fibroblasts had increased levels of oxidized cardiolipins (Figure 7C). LCHAD-deficient cell lines from 2 females (FB944, F942) had oxidized cardiolipin levels similar to FB822, though less than those found in female controls (Figure 7C). No clear-cut pattern of dilysocardiolipins was evident from the heatmaps of male (Figure 8D) and female (Figure 9D) patient fibroblasts when compared to their respective unaffected controls. Levels of dilysocardiolipins were similar to controls in male and female fibroblasts (Figure 7D).

### Oxidized phospholipid and lysophospholipid species in mitochondria differ in patient fibroblasts

**Figure 10:**
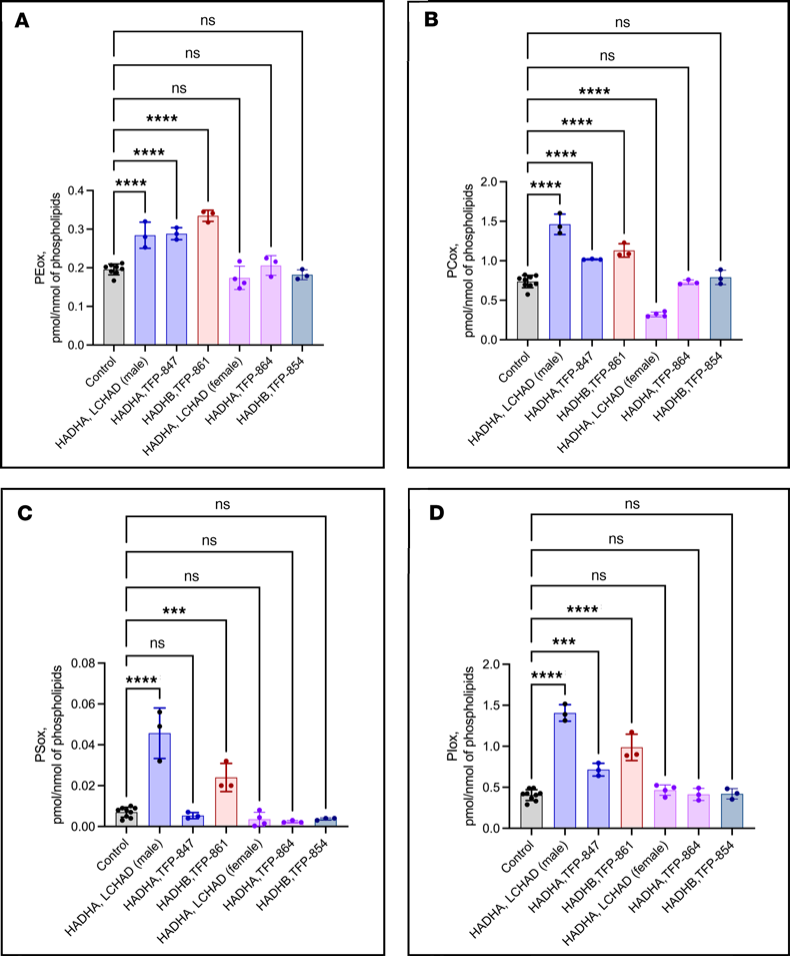
LC-MS/MS assessment of total oxidized phosphatidylethanolamine (PEox), phosphatidylcholine (PCox), phosphatidylserine (PSox), and phosphatidylinositol (PIox) in mitochondria isolated from TFP/LCHAD-deficient fibroblasts and controls. (**A**) Quantification of PEox. (**B**) Quantification of PCox. (**C**) Quantification of PSox. (**D**) Quantification of PIox. A *HADHA* p.E510Q homozygous LCHAD-deficient (FB822) and a *HADHB* compound-heterozygous (FB861) cell line, both from males, had increased levels of all oxidized phospholipid classes. A *HADHA* compound-heterozygous (FB847) cell line, also from a male, had increased levels of all oxidized phospholipid classes, except PSox. All fibroblasts from females (2 p.E510 homozygous LCHAD-deficient, 1 *HADHA*, and 1 *HADHB* compound-heterozygous generalized TFP-deficient) had levels of oxidized phospholipids equivalent to controls. Scatterplots with bars indicating means of 2–3 biological repeats with 2–3 technical repeats for controls and female LCHAD-deficient fibroblasts, for all other cell lines means of 3 technical repeats. SD shown by error bars. One-way ANOVA followed by Dunnett’s multiple comparisons test. *P* values: ***P ≤ 0.001, ****P ≤ 0.0001, compared with FB826, a control cell line from a 40-year-old woman.

Fibroblasts from 2 male patients, 1 homozygous for *HADHA* p.E510Q (FB822) and 1 compound heterozygous for *HADHB* p.A232Lfs*20;p.P294R (FB861), had increased levels of several oxidized phospholipid classes, including oxidized phosphatidylethanolamines (Figure 10A and Supplemental Figure 4E), phosphatidylcholines (Figure 10B and Supplemental Figure 5F), phosphatidylserines (Figure 10C and Supplemental Figure 6D), phosphatidylinositols (Figure 10D and Supplemental Figure 7D), and bis-monoacyl-glycerophosphates (Supplemental Figure 8D). FB847 (*HADHA* c.2146+1G>A;p.K135E compound heterozygous, male) also had increased levels of oxidized phospholipid classes, except for phosphatidylserines. Lysophospholipids (LPLs), including lysophosphatidylethanolamines, lysophosphatidylserines, and lysophosphatidylinositols, had increased levels in all patient-derived cell lines compared with controls (Supplemental Figures 4C, 6C, and 7C).

### Other phospholipids’ contents and molecular species in mitochondria are altered in patient fibroblasts

The patterns of diacyl-phosphatidylethanolamines (PEs, dPEs; Supplemental Figure 4, B and E) and diacyl-phosphatidylcholines (PCs, dPCs; Supplemental Figure 5, B and E) differed significantly among the 3 control cell lines from males. Thus, no genotype-specific pattern of PE and PC was evident. This contrasts with oxidized PE (Supplemental Figure 4E), and oxidized PC (Supplemental Figure 5F), which had increased levels in FB822, FB847, and FB861, and lysophosphatidylethanolamine (Supplemental Figure 4C), which had increased levels in all TFP/LCHAD-deficient fibroblasts, except FB854.

Phosphatidylserine levels were decreased in all TFP/LCHAD-deficient fibroblasts compared with controls (Supplemental Figure 6, B and D). In contrast, phosphatidylinositol levels were increased in all TFP/LCHAD-deficient fibroblasts compared with controls (Supplemental Figure 7, B and D).

All patient-derived fibroblasts had increased levels of bis-monoacyl-glycerophosphates compared with controls (Supplemental Figure 8, B and C). Phosphatidylglycerol and lysophosphatidylglycerol levels varied significantly among controls; consequently, no pattern could be attributed to genotype among TFP/LCHAD-deficient fibroblasts (Supplemental Figure 9, B–D).

### CLs in βTFP mouse liver mitochondria isolates

**Figure 11:**
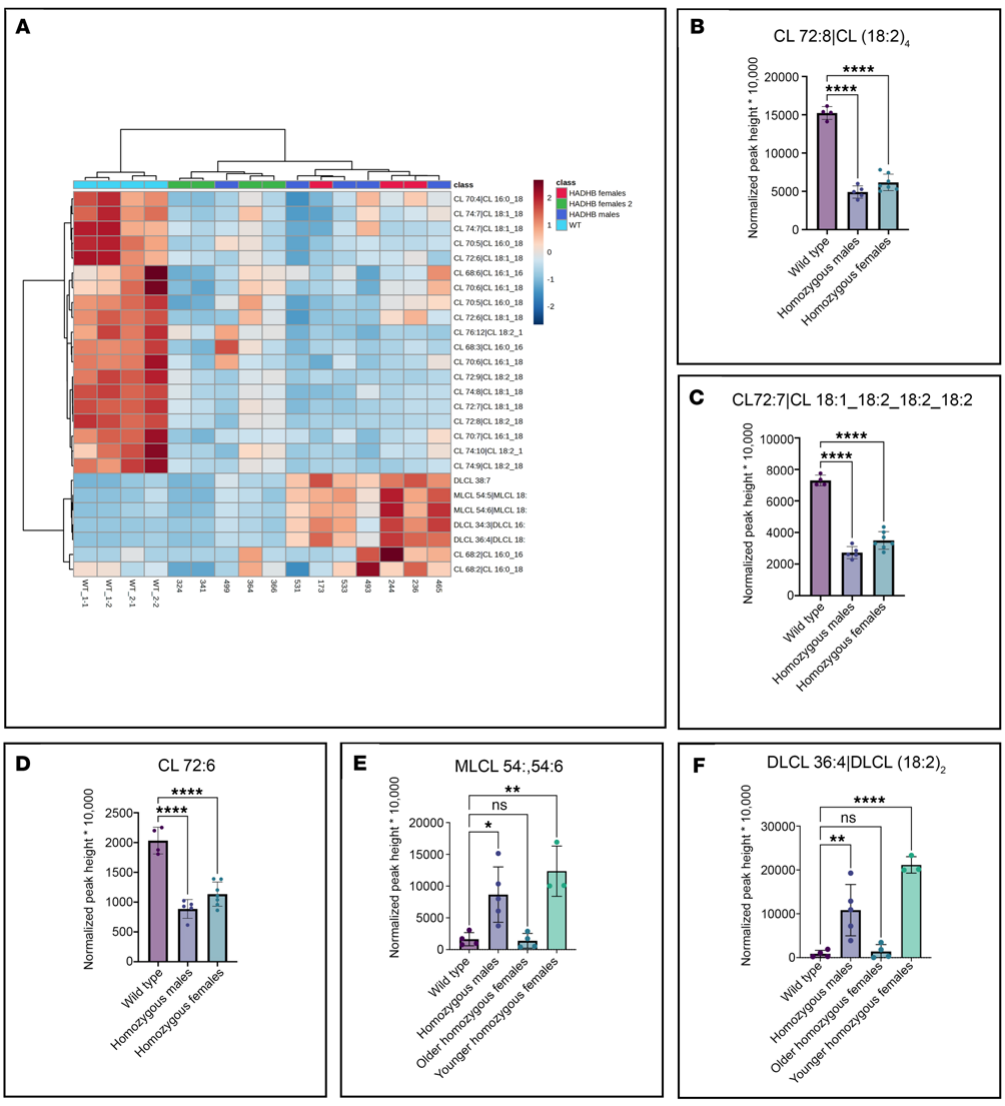
Ultrahigh-performance liquid chromatography (UHPLC) (BEH C_18_)–quadrupole time-of-flight MS assessment of cardiolipins (CL), monolysocardiolipins (MLCL), and dilysocardiolipins (DLCL) in βTFP mouse liver mitochondria. Heatmap after mTIC normalization of metabolites (**A**). mTIC, sum peak height of all structurally annotated compounds. Most abundant species of CL, CL72:8|(18:2)_4_, CL72:7|(18:1,(18:2)_3_), and CL72:6|((18:1)_2_,(18:2)_2_), in βTFP mice (males and females) and controls (**B**–**D**). MLCL and DLCL βTFP male and female mice and controls (**E** and **F**). Levels of these analytes in female mice varied with age. Data are mTIC-normalized peak intensities. Heatmaps are clustered by group of mice and metabolite and are also autoscaled to *Z*-scores: *Z*-score of 0 is marked by white, +3 by dark red, and –3 by dark blue cells, indicating that the sample value is identical to mean value, or 3 SD above or below the mean, respectively. Two groups of homozygous females: red class (homozygous females), 11–19 weeks; green class (homozygous females 2), 23–28 weeks. SD shown by error bars. One-way ANOVA followed by Dunnett’s multiple comparisons test. *P* values: *P ≤ 0.05, **P ≤ 0.01, ****P ≤ 0.0001, compared with FB826, a control cell line from a 40-year-old woman.

CLs 68:3 up to 74:10 were reduced in C57BL/6J-*Hadhb*^m1Ytc^ (βTFP) mice compared with controls (Figure 11A). The most abundant CLs in mouse liver mitochondria, CL72:8|(18:2)_4_, CL72:7|(18:1,(18:2)_3_), and CL72:6|((18:1)_2_,(18:2)_2_), were decreased in both females and males (Figure 11, B–D). MLCLs and dilysocardiolipins were increased in all males (Figure 11, E and F). However, older females (23–28 weeks) had normal levels of these phospholipids, while younger females (11–19 weeks) had a pattern similar to males (Figure 11, E and F).

**Figure 12:**
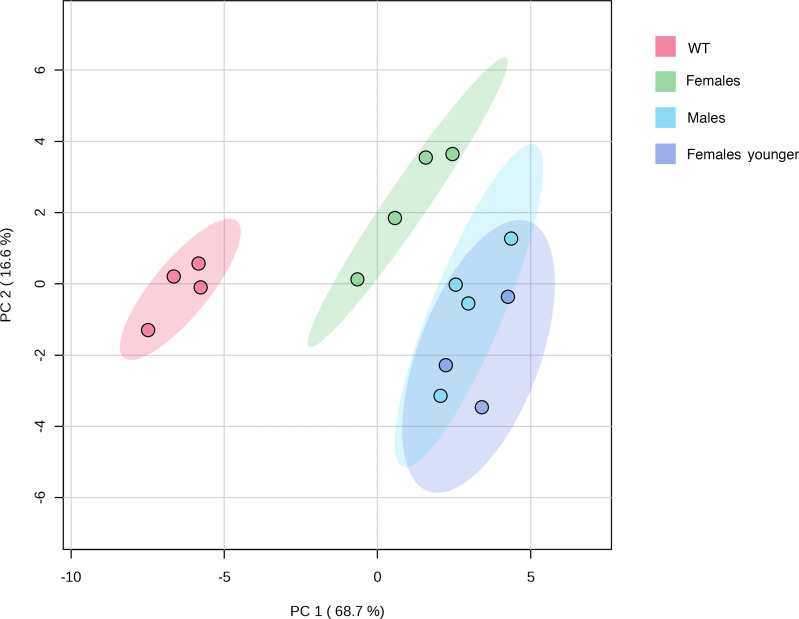
PCA model of the cardiolipidomes (cardiolipins, monolysocardiolipins, and dilysocardiolipins) from 4 groups of mice: wild-type (WT), (older) females, younger females, and males. WT samples were scattered over the left region of the plot, male (and younger female) samples were scattered across the right region of the plot, and the (older) female samples were in between. The PCA model captured 93.3% of the total variance (PC1, 68.7%; PC2, 16.6%; PC3, 8.0%). Four samples for each group, except younger females, represented by 3 samples.

Principal component analysis (PCA) showed that the cardiolipidomes from 3 groups of mice (wild-type, older females, and males) were clearly separated from each other (Figure 12). Wild-type samples were scattered over the left region of the plot, male (and younger female) samples were scattered across the right region of the plot, and the older female samples were in between. The PCA model captured 93.3% of the total variance (PC1, 68.7%; PC2, 16.6%; PC3, 8.0%) (Figure 12).

## Discussion

Patient-derived fibroblasts with *HADHA* and or *HADHB* truncating variants had a decrease in both TFP subunit proteins, verifying previous reports that TFP tetramer formation or stability requires the presence of α and β subunits (24, 30). In contrast, fibroblasts harboring only missense mutations (FB822 and FB854) had stable TFP protein. The 2 canonical splice site variants’ mRNAs were not subject to nonsense mediated mRNA decay (NMD). Other mutations in the 5′ end of *HADHA* (intron 19 and exon 20), such as c.2146+1G>A, have also been reported to escape NMD (10). Both *HADHA* c.919-2A>G and c.2146+1G>A have been reported in ClinVar as pathogenic and likely pathogenic. *HADHA* c.919-2A>G was also previously described in a patient with peripheral neuropathy and episodes of rhabdomyolysis with myoglobinuria (31). The third truncating variant in our case series is a small deletion in exon 9 of *HADHB* that creates a frameshift (p.A232Lfs*20). In contrast with the splice site variants, droplet digital PCR (ddPCR) data suggest that its mRNA undergoes NMD.

The unpublished missense exon 5 *HADHA* c.403A>G (p.K135E) variant has conflicting interpretations of pathogenicity in ClinVar. Although its structural analysis predicts it to be tolerated, a severe decrease in both HADHA and HADHB protein and a reduction of FAO flux to 9% of control levels in FB847 support it as disease causing. *HADHA* c.703C>T (p.R235W) is at a position that is conserved across species (PolyPhen-2, Mutation Taster). Structural analysis predicts it to destabilize α and β subunits’ interaction, consistent with our functional results. Three patients homozygous for p.R235W have previously been described (10). In agreement with our findings (FB864; Figure 6A), all were reported to have reduced LCHAD and LKAT activities, as well as FAO flux in fibroblasts or lymphocytes. Structural analysis of the missense *HADHB* exon 10 variant c.881C>G (p.P294R) predicts it to destabilize α and β subunits’ interaction, consistent with diminished levels of HADHA and HADHB protein in cell extracts along with reduced FAO flux in fibroblasts (FB861; Figure 6A).

While isolated LCHAD or generalized TFP deficiency leads to an understandable impairment in cellular FAO, patients with both defects have some clinical symptoms that differ from other long-chain FAODs, overlapping those seen in defects of OXPHOS (33, 34). We have previously shown that the proteins of long-chain FAO and ETC supercomplexes interact functionally and physically in a multiprotein complex, with the LCHAD moiety of αTFP specifically interacting with the NADH-binding domain matrix arm of OXPHOS complex I (28, 29). Thus, we proposed that mutations in long-chain FAO proteins could disrupt this interaction, leading to a secondary impairment in ETC complexes and supercomplexes. Indeed, knockout mice deficient in VLCAD protein show decreased ETC supercomplexes (29). However, the recent identification of a fourth function for TFP in CL remodeling offers a second possibility that a reduction in mature CL and an increase in MLCL lead to a disruption of the IMM, destabilizing the respiratory chain supercomplexes (14, 15). Indeed, mutations in *HADHA* have been shown to alter cellular CLs and impair cellular oxygen consumption (32, 35). In keeping with these reports, we found that the common LCHAD mutation in a homozygous state produced significant impairments in mitochondrial bioenergetics and altered acylcarnitine and CL profiles in patient-derived fibroblasts, though α and β subunit protein and mRNA levels were preserved. Jones et al. suggested that homozygous p.E510Q led to a greater accumulation of long-chain 3-hydroxy fatty acids than heterozygous p.E510Q (36). The effects of the p.E510Q mutation are not surprising as Glu510 is part of the His-Glu catalytic dyad in the active site of LCHAD, presumably affecting the MLCLAT activity as well (8, 9). Additional experiments are necessary to validate the catalytic triad αH498-αGlu510-αT547 predicted by our structural modeling. Of note, it is not clear if the entire TFP molecule is the active moiety containing the MLCLAT activity or a smaller independent 59 kDa protein derived from alternative splicing of the *HADHA* mRNA. Our results are consistent with either possibility.

Miklas et al. (32) investigated CL and MLCL profiles in CRISPR/Cas9 system–generated clones with deletions and insertions in exon 1 of *HADHA* from unaffected male donor human induced pluripotent stem cells (hiPSCs) (GM25256*I). While we found lower levels of all species of CLs in patient and mouse mitochondria isolates, these authors found increased levels of CLs containing C16 and C18:1 in CRISPR/Cas9-generated clones. Additionally, we found increased levels of MLCL in all 3 male-derived patient fibroblasts and in male and younger female mice but not in 4 female-derived TFP/LCHAD-deficient fibroblasts and in older female mice, demonstrating an important sex-dependent effect on phospholipid profiles. The previous study did not find a significant increase in MLCL levels in male hiPSC-derived clones and female-derived clones were not studied (32). The authors inferred that HADHA remodels CL but not MLCL, in disagreement with Taylor et al. (14) and Taylor and Hatch (15), who originally described the MLCLAT function linked to HADHA. Our results are in alignment with these latter authors and also point to a requirement for HADHB to stabilize HADHA and MLCLAT activity. Alatibi et al. (37) found an increase in total CL levels in fibroblasts from 3 patients with LCHAD deficiency, homozygous for p.E510Q. Although they attributed this increase to a CL remodeling dysfunction, it is hard to reconcile their data with a blockade of this pathway and the more logical decrease of the product of MLCLAT enzymatic action seen in our data and those of Miklas et al. (32).

In addition to the bioenergetic and CL changes seen in isolated LCHAD-deficient cells, we have identified similar changes in cells with generalized TFP deficiency. Patient-derived fibroblasts with null mutations in *HADHA* (FB847, FB864) and *HADHB* (FB861) showed considerable global deficits in mitochondrial bioenergetics, with decreases in maximal respiration and spare respiratory capacity compared with control cells. Cells homozygous for the common LCHAD mutation as well as cells with an *HADHB* frameshift variant (FB861) showed a substantial decrease in ATP production, also consistent with a global bioenergetics defect. In contrast, ATP production was unaffected in FB864 with the *HADHA* c.919-2A>G splice variant and even higher than controls in FB847 with the *HADHA* c.2146+1G>A splice variant. A reduction of maximal respiration, spare respiratory capacity, and ATP production has previously been reported in cells homozygous for the common LCHAD mutation and for *HADHB* c.182G>A (p.R61H), but those from a compound-heterozygous patient with 2 missense variants in *HADHB* [c.182G>A (p.R61H);c.740G>A (p.R247H)] were not different from controls (38). Here, a compound-heterozygous patient with 2 *HADHB* missense variants (FB854) had minimally reduced maximal respiration and spare respiratory capacity. Some *HADHB* missense variants seem to be less detrimental to mitochondrial bioenergetics. This contrasts with results from similar experiments in cells from patients with VLCAD deficiency, where mitochondrial bioenergetics were universally impaired compared with controls regardless of genotype (39). Thus, our results indicate a genotype-specific mutation effect on OXPHOS in TFP/LCHAD-deficient cells, though it remains to be determined if this is due to a direct impairment of the interaction between LCHAD and OXPHOS complex I or to an effect mediated by CL abnormalities.

To examine the role of CL abnormalities in TFP/LCHAD pathophysiology, mitochondria isolated from TFP/LCHAD-deficient fibroblasts and from βTFP mouse liver were submitted to lipidomics analysis. In aggregate, TFP/LCHAD mitochondria from human fibroblasts had decreased levels of mature CLs, though the levels of MLCLs were sex dependent, with males presenting a significant increase while females had no accumulation of these phospholipids. Quantitative and semiquantitative (heatmaps) analyses clearly distinguished mutant fibroblasts from males and females based on the cardiolipidome (CL, MLCL, dilysocardiolipin, oxidized cardiolipin) profiles (Figures 8 and 9). Homozygous p.E510Q fibroblasts from a male (FB822) and 2 females (FB944 and FB942) had clearly distinct cardiolipidomes (Figures 8 and 9). The increase in oxidized cardiolipins seen in homozygous p.E510Q and compound-heterozygous *HADHB* male fibroblasts (FB822 and FB861, respectively) showed a negative correlation with mitochondrial bioenergetics, as these were the cells with the lowest results for basal and maximal respiration and spare respiratory capacity (Figure 5). βTFP mice also had a decrease in CLs and a sex-dependent (and perhaps age-dependent) increase in MLCLs. To reconcile the presence of the MLCLAT activity in αTFP LCHAD with our findings of decreased CL levels in *HADHB*-mutated human and mouse mitochondria, the requirement of βTFP for MLCLAT activity, associated with integral αTFP or MLCLAT-1, must be reappraised.

Three-dimensional PCA of CLs, MLCLs, and dilysocardiolipins of βTFP mouse liver mitochondria clearly showed 3 groups, wild-type, 23- to 28-week-old homozygous females, and a group comprising 19- to 31-week-old homozygous males and 11- to 19-week-old homozygous females (Figure 12). Older homozygous females were closer to wild-type than homozygous males and younger females, substantiating the findings of a decrease in CL levels in all β-TFP mice groups and a simultaneous increase in MLCLs and dilysocardiolipins in males and younger females.

A previous report described a reduction in total CL and CL(18:2)_4_ and unaltered MLCL(18:2)_3_ levels in liver mitochondria from heterozygous αTFP-knockout mice (35). The difference in genetic defect and the fact that the animals were not homozygous deficient complicate comparison to our studies; however, our βTFP mouse model also showed decreased total CL and CL(18:2)_4_.

Studies concerning sex differences in CLs’ and other phospholipids’ contents and profiles in mitochondria are scarce. In humans, a meta-analysis of several mitochondria parameters, including CL content, found that only 2 measures demonstrated aggregate binary sex differences: higher mitochondrial content in women’s white adipose tissue and isolated leukocyte subpopulations and higher ROS production in men’s skeletal muscle (40). In rodents, sex differences were reported in CL profiles of liver and kidney, among several tissues that were analyzed in rats (41), and of cerebral cortex in very early postnatal life in mice (42). However, our results do not necessarily agree with the latter study, as our data displayed sex-dependent differences in MLCL and dilysocardiolipin contents, not in CL profiles; the former compounds were not analyzed by those authors. Moreover, while in the latter study targeted lipidomics analyses were performed, untargeted lipidomics analyses were done in our study.

Increased MLCL/CL ratios were found in p.E510Q homozygous (FB822, FB944, FB942), FB847, and FB861 fibroblasts. However, all results were below a value of 0.08. In Barth syndrome (BTHS), an X-linked recessive inborn error of metabolism caused by pathogenic mutations in the *TAFFAZIN* gene, MLCL/CL ratios are at least 0.3 (18, 20, 43). MLCL/CL ratios reported in lymphoblasts from patients with BTHS are 690-fold higher than control levels (44), while in TFP/LCHAD-deficient fibroblasts showing an increase in MLCL/CL ratios, they were 1.4-fold higher than controls in cells from females and 3.8-fold higher than controls in cells from males. The protein encoded by *TAFFAZIN*, a phospholipid-LPL transacylase, mainly replaces acyl groups of CL with linoleic acid (18:2), being responsible for the generation of CL(18:2)_4_, the principal CL species in mammalian cardiac and skeletal muscle (16, 45). However, there might be a pathophysiological difference between TFP/LCHAD-deficient cells with different MLCL/CL ratios. Of note, the MLCL/CL ratio in our βTFP-deficient male mice was 16-fold higher than in wild-type mice. Most of these mice (61%) develop cardiac fibrosis at a mean age of 6 months (our unpublished observations). The possible contribution of CL and MLCL alterations to the development of cardiomyopathy in patients with TFP/LCHAD deficiency remains to be investigated. The occurrence of cardiomyopathy in the female patient from whom FB854, which had the lowest MLCL/CL ratio, was obtained precludes us from making conclusions about the contribution of CL remodeling defect to the pathogenesis of this complication. Further studies with cardiomyocytes or human postmortem or posttransplantation heart samples of patients with TFP/LCHAD deficiency are necessary.

Human acyl-CoA–dependent MLCLAT-1 activity was first identified as a 59 kDa protein in cells identical to the 74 kDa αTFP minus its first 227 amino acids (15). It was subsequently reported that full-length purified human recombinant αTFP acyl-CoA also had acyltransferase activity (14). Thus, the in vivo nature of the MLCLAT remains unclear. In our study, fibroblasts missing the 59 kDa protein as well as having reduced 74 kDa protein (FB847 and FB861) had a small increase in MLCL/CL ratios (3.1- and 4.7-fold higher than controls, respectively). Moreover, the homozygous common LCHAD variant fibroblasts had a sex-dependent increase in MLCL/CL ratios (1.4-fold and 3.6-fold higher than controls in females and males, respectively). However, both the 74 kDa and 59 kDa *HADHA*–derived proteins were present on Western blots, albeit the latter was reduced compared with controls. Since this variant alters a critical amino acid in the catalytic dyad, eliminating LCHAD activity, it seems likely that the MLCLAT catalytic site, which is shared with LCHAD, is also impaired.

The relative elevation of the concentrations of several LPLs in TFP/LCHAD-deficient fibroblast mitochondria might be related to a dysfunction of lysoacyltranferases, enzymes that incorporate acyl chains into LPL, generating corresponding phospholipids (46–48).

The increase in several oxidized phospholipid classes found in some of the patient fibroblasts could reflect elevated oxidative stress due to mitochondrial dysfunction (49). In turn, this could lead to a vicious cycle with raised ROS production, aggravating even more mitochondrial homeostasis (50). This feed-forward loop could promote the symptoms specific to TFP/LCHAD deficiency: cardiomyopathy, peripheral neuropathy, and retinopathy, as well as those that may be related to inflammation, e.g., rhabdomyolysis (51).

In summary, TFP/LCHAD fibroblasts exhibit substantial impairment in CL maturation along with impairment of mitochondrial bioenergetics. Synthetic peptides such as SS-31 (elamipretide) that selectively bind to CL have been proposed as a possible therapeutic agent to improve mitochondrial function in several conditions affecting CL synthesis or structure, including BTHS (52). Our preclinical studies, with both animal models and human cells, add TFP/LCHAD deficiency to this list. However, caution is necessary in considering such a therapy as our series of TFP/LCHAD-deficient cells showed variable TFP protein levels, mitochondrial bioenergetics disruption, and CL profile alterations that could respond differently to drugs targeting mitochondrial bioenergetics and CL metabolism. Therapy in this disease is likely to be genotype specific at least to some extent. Evaluation of more patient cell lines as well as studies of other tissues besides liver of suitable mouse models will be necessary to establish the magnitude of such an effect.

## Methods

### Sex as a biological variable

Our study examined human fibroblast cell lines from both unaffected (controls) and TFP/LCHAD-deficient male and female patients. Male and female βTFP mutant mice were also examined. Findings are reported for both sexes in humans and mice.

### Participants and fibroblast cell lines

Seven patients were previously diagnosed with TFP/LCHAD deficiency based on at least 2 criteria: plasma acylcarnitine profile and genomic DNA analysis showing biallelic (homozygous or compound heterozygous) pathogenic or likely pathogenic variants as defined by the guidelines of the American College of Medical Genetics (53). Supplemental Table 1 presents the clinical characteristics of these patients. Fibroblast cell lines established from skin biopsy samples of these patients had been previously obtained for other clinical testing. A list of cell lines and their mutations is shown in Supplemental Table 2. Skin biopsies of the patients included in this article were performed from 3 to 16 years, average 8 years. FB861 has previously been reported (54).

Control fibroblasts were dermal fibroblast cell lines from the American Type Culture Collection (ATCC) — FB826 (PCS-201-012TM) — and from the NIH National Institute of General Medical Sciences Human Genetic Cell Repository at the Coriell Institute for Medical Research — FB902 (GM23976), FB554 (GM08398), and FB549 (GM05565). FB826 from a 40-year-old healthy woman was used as a control in almost all experiments. For lipidomics, the control fibroblasts were FB549, FB554, and FB902, from 3-year-old, 8-year-old, and 22-year-old healthy males, and FB550, FB552, and FB557, from 19-year-old, 9-year-old, and 2-day-old healthy females, respectively. Details of these control fibroblasts are displayed in Supplemental Table 3.

### Animal studies

Previously reported C57BL/6J-*Hadhb*^m1Ytc^ (β-TFP) mice were used in this study (55). In-house WT C57BL/6J, The Jackson Laboratory Strain 000664, mice were used as controls. Animals were euthanized by O_2_/CO_2_ inhalation, and liver samples were then collected for mitochondrial isolation and lipidomics analyses.

#### Genotype confirmation.

Genotypes determined by clinical laboratories were confirmed prior to use of fibroblast cell lines by standard techniques (11). Information on primers for PCR amplification was provided by Lodewijk Ijlst, Amsterdam University Medical Centers, the Netherlands (Supplemental Table 6).

### Structure analysis and variant pathogenicity criteria

#### Structure analysis.

Structure analysis of the variant protein αTFP p.E510Q and other missense mutations, including their molecular dynamics simulations, can be found in Supplemental Methods.

#### Variant pathogenicity criteria and allele frequency.

For the assessment of variant pathogenicity and allele frequency, the databases HGMD (http://www.hgmd.cf.ac.uk/ac/index.php), ClinVar (http://www.ncbi.nlm.nih.gov/clinvar/), and gnomAD were utilized (56). The in silico predictive tools PROVEAN, SIFT, PolyPhen-2, and Mutation Taster were employed to assess the potential biological effect of *HADHA* and *HADHB* missense variants. Mutation Taster was used to evaluate small deletions and splicing site variants.

### Cell culture

Dermal fibroblast cell lines derived from TFP/LCHAD-deficient patients and controls were cultivated as previously described (39, 57). The passage number of the cells used in this study was maintained between 4 and 7.

### Western blot

Western blotting was performed on lysates from cultured fibroblasts as previously described (58), with the ensuing modifications: fibroblast pellets were sonicated twice for 10 seconds each using a Sonic Dismembrator model 550 (Thermo Fisher Scientific); Laemmli buffer (Bio-Rad Laboratories), under reducing conditions, was added to the samples that were then heated at 95°C for 10 minutes. The following primary antibodies were used: mouse monoclonal IgG_1_ anti-HADHA (1:1,000, Proteintech; catalog 60250-1-Ig; RRID:AB_2881371) and mouse monoclonal IgG_2a_ anti-HADHB (1:1,000, Santa Cruz Biotechnology; catalog sc-271495, RRID:AB_10649497). Rabbit monoclonal anti-TOMM20 (1:5,000, Abcam; catalog ab186735, RRID:AB_2889972) was employed as a mitochondrial marker. The secondary antibodies were goat anti-mouse HRP-conjugated antibody (1:3,000, Bio-Rad Laboratories; catalog 1706516, RRID:AB_2921252) and/or goat anti-rabbit HRP-conjugated antibody (1:3,000, Bio-Rad Laboratories; catalog 1706515, RRID:AB_2617112).

### mRNA quantification by ddPCR

RNA was isolated from fibroblasts using the RNeasy Plus Mini Kit (QIAGEN). Single-strand cDNA was synthesized from RNA, using SuperScript IV Vilo Master Mix (Invitrogen) according to the manufacturer’s protocol. PrimeTime qPCR probe assays (IDT) utilizing FAM-labeled probe sets for *HADHA* normal and variant (c.403A/c.403G; c.1528G/c.1528C) and *HADHB* (c.881C/c.881G) were custom designed using a combination of NCBI Primer-Blast (59) and the IDT PrimerQuest Tool. A HEX-labeled probe for *GAPDH* was used for cDNA expression control. Primers and probes are listed in Supplemental Table 7. Each 20 μL ddPCR had 2 μL input of diluted cDNA (approximately 80 or 8 ng cDNA) using the ddPCR for probes (no dUTP) 2× master mix (Bio-Rad Laboratories) and manufacturer-suggested reagent and thermocycling parameters. An automated ddPCR droplet generator and QX200 reader (Bio-Rad Laboratories) were used to run the reactions, and the data were analyzed with manual 2D amplitude thresholds applied in QuantaSoft (Bio-Rad Laboratories).

### Mitochondrial respiration

The key parameters of mitochondrial bioenergetics of the TFP/LCHAD-deficient and control culture fibroblasts were evaluated by directly measuring the OCR in a Seahorse XFe96 Extracellular Flux Analyzer (Agilent Technologies), according to previously published protocols (39, 57, 60), with minor modifications: cells were seeded in an uncoated 96-well cell culture microplate at a density of 30,000 cells per well in complete DMEM with 4.5 g/L d-glucose. The microplate was incubated overnight at 37°C in a 5% CO_2_ humidified atmosphere. Each fibroblast cell line was seeded in at least 12 technical replicates. Culture medium was replaced with Seahorse XF assay medium devoid of glucose, supplemented with 1 mM pyruvate and 2 mM L-glutamine, and the microplate was incubated for 1 hour at 37°C in a non-CO_2_ incubator. Seahorse XF assay medium does not contain fetal bovine serum, and its constituents are based on DMEM, with no phenol red, sodium bicarbonate, glucose, glutamine, or sodium pyruvate. The FCCP concentration used, 1.0 μM, was previously determined in a series of experiments employing a commercially available mouse C2C12 myoblast cell line (ATCC, catalog CRL-1772) and a tafazzin-KO cell line generated by CRISPR-mediated inactivation of tafazzin in C2C12 cell line (61). The FCCP concentrations tested varied from 0.25 μM to 2.0, and 1.0 μM gave the best results for maximal respiration and spare respiratory capacity. OCR measurements were performed as previously described (39, 57, 60).

### FAO flux analysis

A modified tritium release assay was employed to evaluate FAO flux as previously described (57, 62). Three technical replicates of each individual cell line were prepared with triplicate blanks (cell-free wells). Standards contained a 10 μL aliquot of the incubation mix with 3 mL of deionized water and 10 mL of scintillation fluid.

### ESI-MS/MS–based acylcarnitine profiling

Acylcarnitine profiling in fibroblasts was performed after a challenge with unlabeled palmitic acid as previously described (63–65). Free carnitine and acetylcarnitine were monitored using multiple reaction monitoring, while all other acylcarnitines were monitored by the precursor ion scan of *m/z* 85 and scanning a range from *m/z* 270 to 502.

### Mitochondria isolation

#### Fibroblasts.

Mitochondria Isolation Kit for Cultured Cells (Thermo Fisher Scientific) was employed for isolating mitochondria from at least 2 × 10^6^ cells. The protocol was performed according to the manufacturer’s instructions. Halt Protease Inhibitor Cocktail, EDTA-Free (100×) (Thermo Fisher Scientific), was added to the amounts of the kit’s reagents A and C immediately prior to the isolation procedure. Cells were lysed in a Dounce tissue grinder on ice. No less than 80% lysis was accepted.

#### Mouse liver.

Liver samples were homogenized in mitochondrial separation buffer: 0.25 M sucrose, 1 mM EDTA, 2.5% glycerol, 50 mM phosphate buffer (pH 8.0), and freshly added protease inhibitor cocktail (Roche). Nuclei and cell debris were first removed by a 10-minute centrifugation at 700*g*, and then the supernatant was collected and centrifuged at 14,000*g* for 15 minutes at 4°C. Mitochondrial fractions were maintained at –80°C until analysis.

### Identification and quantification of human fibroblast mitochondrial phospholipids (including CL) and oxidized phospholipids by LC-MS/MS

Phospholipids (including CL) and oxidized phospholipids in mitochondria isolated from fibroblasts were measured by LC-MS/MS as essentially detailed elsewhere (66–68). Total lipids were extracted using the Folch method (69). The total phosphate content of the lipids were quantified by a micro method (66, 70). Samples corresponding to approximately 2.5 nmol of total phosphate were added with the appropriate internal standards and injected into the LC-MS/MS system for lipid identification and quantification. LC-MS/MS system consisted of a Dionex Ultimate 3000 RSLCnano System coupled online to a Q-Exactive hybrid Quadrupole-Orbitrap mass spectrometer (Thermo Fisher Scientific) equipped with a normal phase column (Silica Luna 3 μm, 100 Å, 150 × 2 mm, Phenomenex). Analysis was performed in negative-ion mode at a resolution of 140,000 for the full MS scan in a data-dependent mode.

Analysis of raw LC/MS data was performed using the software package Compound Discoverer 2.1 (Thermo Fisher Scientific) with an in-house–generated analysis workflow and oxidized phospholipid database. Peaks with signal-to-noise ratio of more than 3 were identified and searched against phospholipid database. Lipids were further filtered by retention time (±3 minutes from the internal standard) and confirmed by a fragmentation mass spectrum. Values for *m/z* were matched within 5.0 parts per million to identify the lipid species. Peak areas were used for quantification of phospholipid species.

### Mouse liver mitochondria lipidomics

Samples were extracted using Matyash extraction procedure (71). The organic phase was dried down and resuspended in a solution of 9:1 methanol/toluene containing deuterated internal standards (UltimateSPLASH ONE, Avanti Polar Lipids), 12-[[(cyclohexylamino)carbonyl]amino]-dodecanoic acid, and supplemental standards and used for LC-MS/MS analysis. Samples were injected into a Vanquish UHPLC liquid chromatography system coupled to a Q-Exactive HF orbital ion trap mass spectrometer (Thermo Fisher Scientific) and analyzed in both positive and negative mode. An ACQUITY Premier BEH C18 column (1.7 μm, 2.1 × 50 mm) (Waters) was used to separate complex lipids. Data were collected with a scan range of *m/z* 120–1,700 and 60,000 mass resolution.

### Statistics

The statistical software package GraphPad Prism version 9.0.0 for Mac (GraphPad Software) was used to analyze the results of mitochondrial respiration parameters, FAO flux analysis, acylcarnitine profiles, and mouse liver lipidomics. Statistical analysis of fibroblast lipidomics results was performed using SPSS Statistics 25 (IBM Corporation). Mouse liver lipidomics data were analyzed by MetaboAnalyst 5.0. Iglewicz and Hoaglin’s (72) and ROUT methods were employed for the detection of outliers. One-way ANOVA followed by Dunnett’s multiple comparisons test was used for comparing the FB826 control fibroblast result means for mitochondrial respiration parameters, FAO flux analysis, and acylcarnitine profiles and means of the TFP/LCHAD-deficient fibroblasts. One-way ANOVA followed by Holm-Šídák multiple comparisons test was employed when there was more than 1 control, e.g., control fibroblasts from males and females. The level of significance for all statistical tests was *P* ≤ 0.05. Lipidomics heatmaps were elaborated based on *Z*-scores.

### Study approval

Human fibroblast cell lines from patients confirmed to have TFP/LCHAD deficiency have been previously obtained for other clinical testing, so no procedures specific to this study were performed. Consent to provide the samples was obtained per referring physicians’ institutional review boards. Patients in the UPMC system provided written consent for the study, and one of the authors shared results with them at their request. For patients from Children’s Hospital of Pittsburgh, informed consent was obtained for testing and storage of a fibroblast or tissue sample as approved by our IRB Protocol CR19030195-010.

All animal studies were done in accordance with research protocols approved by the University of Pittsburgh IACUC: Protocol IS00020939.

### Data availability

Lipidomic analysis data of mitochondria isolated from human fibroblasts have been deposited to Zenodo (http://dx.doi.org/10.5281/zenodo.8078578). Original acylcarnitine profiles (http://dx.doi.org/10.17632/hs9t22ydy7.1), Seahorse XFe96 Extracellular Flux Analyzer assay result files (http://dx.doi.org/10.17632/8jch3rtcb2.1), and Western blot images (http://dx.doi.org/10.17632/dr4fhz7smm.1) have been deposited to Mendeley Data. Supporting data values for all individual points shown in graphs and values behind any reported means, are also publicly available in the Supporting Data Values file.

## Author contributions

EVN, AHE, VK, HB, and JV conceived the study; EVN, MW, EK, CVL, GZ, TSA, YYT, VAT, VK, HB, and JV developed methodology; EVN, EK, CVL, AWM, GZ, TSA, YYT, and VAT performed formal analysis; EVN, MW, AJS, LF, EK, YW, CVL, AWM, GZ, TSA, YYT, and VAT investigated; EVN and JV wrote the original draft; EVN, EK, TSA, YYT, and JV reviewed and edited the manuscript; EVN, VK, HB, and JV acquired funding; EVN, EK, CVL, AWM, GZ, TSA, VK, HB, and JV provided resources; and VK, HB, and JV supervised the study.

## Supplementary Material

Supplemental data

Unedited blot and gel images

Supporting data values

## Version 1 08/01/2024

In-Press Preview

## Version 2 09/10/2024

Electronic publication
