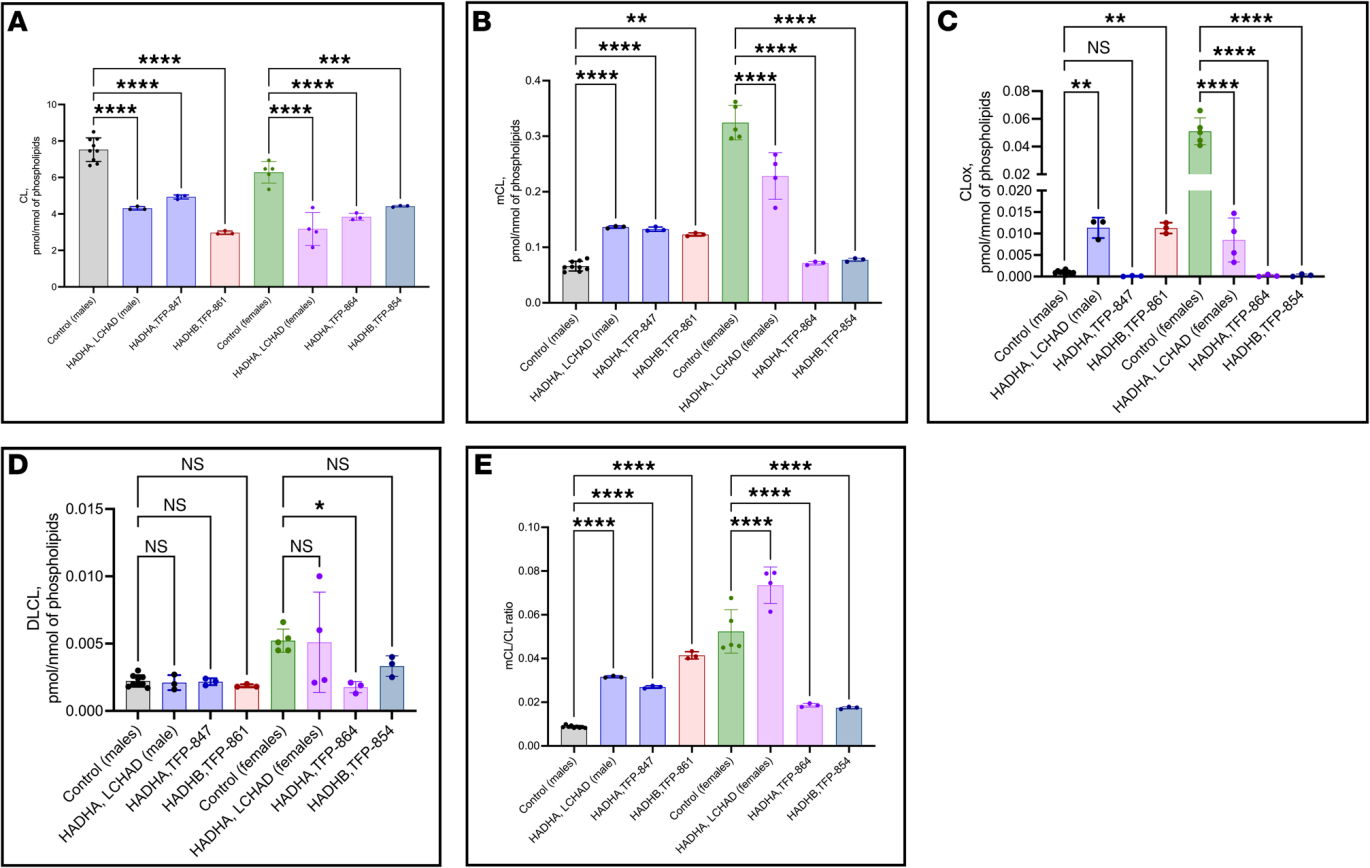# Corrigendum to: Mitochondrial bioenergetics and cardiolipin remodeling abnormalities in mitochondrial trifunctional protein deficiency

**DOI:** 10.1172/jci.insight.206548

**Published:** 2026-04-08

**Authors:** Eduardo Vieira Neto, Meicheng Wang, Austin J. Szuminsky, Lethicia Ferraro, Erik Koppes, Yudong Wang, Clinton Van’t Land, Al-Walid Mohsen, Geancarlo Zanatta, Areeg H. El-Gharbawy, Tamil S. Anthonymuthu, Yulia Y. Tyurina, Vladimir A. Tyurin, Valerian Kagan, Hülya Bayır, Jerry Vockley

Original citation: *JCI Insight*. 2024;9(17):e176887. https://doi.org/10.1172/jci.insight.176887

Citation for this corrigendum: *JCI Insight*. 2026;11(7):e206548. https://doi.org/10.1172/jci.insight.206548

After publication, the authors became aware of errors in figure legends, figure panel callouts, statistical comparison descriptions, and figure labels. In the legend for Figure 4E, the allele-specific expression descriptions in panels E–H were incorrect. In the Results section, under the heading “Expression of variant mRNA,” the original callouts for Figures 4A, 4B, 4E, and 4F were incorrect. The legends for Figure 7 and Figure 10 incorrectly specified the control group used for statistical comparisons as FB826, a control cell line from a 40-year-old woman. Patient numbering in Supplemental Table 2 was incorrect. Finally, the HADHA and HADHB groups were incorrectly labeled in Figure 7. The authors declare that these errors do not affect the results, interpretation, or conclusions of the study. The online HTML and PDF versions of the manuscript and the Supplemental Data Values file have been updated to correct these issues.

The authors regret the errors.

## Supplementary Material

Supplemental data

Supporting data values

## Figures and Tables

**Figure 7:**