# Supplementary material for: Mitochondrial bioenergetics and cardiolipin remodeling abnormalities in mitochondrial trifunctional protein deficiency
Source: JCI Insight. 2024 Sep 10;9(17):e176887. doi: 10.1172/jci.insight.176887 (PMC11385086; doi:10.1172/jci.insight.176887)
Supplement: Supplemental data [file jciinsight-9-176887-s089.pdf]

**Supplemental Table 1.** Clinical characteristics of patients with TFP/LCHAD deficiency included in the study

| Patient | Cell line | Sex | Current state | TFP/LCHAD Deficiency | Hypoglycemia | Hypotonia/Motor Delay | Muscle weakness | Rhabdomyolysis | Cardiomyopathy | Peripheral neuropathy | Retinopathy | Maternal HELLP syndrome |
|---|---|---|---|---|---|---|---|---|---|---|---|---|
| 1 | FB864 | F | 12y, AFOs | TFP (*HADHA*) | N | N | Y | Y | N | Y | N | N |
| 2 | FB847 | M | 7y | TFP (*HADHA*) | N | N | Y | Y | N | N | N | Y |
| 3 | FB822 | M | 12y | LCHAD | N | N | N | Y | Y (reversed by treatment) | N | N | Y |
| 4 | FB944 | F | Died 19y | LCHAD | Y | N | Y | Y | Y | Y | Y | N |
| 5 | FB942 | F | Died 19y | LCHAD | Y | N | Y | Y | N | N | Y | Y |
| 6 | FB854 | F | Died 4y | TFP (*HADHB*) | Y | Y | Y | Y | Y (severe, dilated/ hypertrophic) | Y | N | N |
| 7 | FB861 | M | 20y, wheelchair-bound | TFP (*HADHB*) | Y | Y | Y | Y | N | Y | N | N |

Legend: AFOs, Ankle-Foot Orthoses; HELLP syndrome, hemolysis, elevated liver enzymes, and low platelets syndrome

**Supplemental Table 2.** TFP/LCHAD-deficient fibroblast cell lines and their respective genotypes

| Patient | Sex | TFP/LCHAD Deficiency | Cell line | Gene Affected | Allele 1 | Amino acid change | Reference sequence | Allele 2 | Amino acid change | Reference sequence |
|---|---|---|---|---|---|---|---|---|---|---|
| 1 | F | TFP | FB864 | *HADHA* | c.919-2A>G | — | NG_007121.1 | c.703C>T | p.R235W | NM_000182.5 |
| 2 | M | TFP | FB847 | *HADHA* | c.2146+1G>A | — | NG_007121.1 | c.403A>G | p.K135E | NM_000182.5 |
| 3 | M | LCHAD | FB822 | *HADHA* | c.1528G>C | p.E510Q | NM_000182.5 | c.1528G>C | p.E510Q | NM_000182.5 |
| 4 | F | LCHAD | FB944 | *HADHA* | c.1528G>C | p.E510Q | NM_000182.5 | c.1528G>C | p.E510Q | NM_000182.5 |
| 5 | F | LCHAD | FB942 | *HADHA* | c.1528G>C | p.E510Q | NM_000182.5 | c.1528G>C | p.E510Q | NM_000182.5 |
| 4 | F | TFP | FB854 | *HADHB* | c.1165A>G | p.N389D | NM_000183.3 | c.1289T>C | p.F430S | NM_000183.3 |
| 5 | M | TFP | FB861 | *HADHB* | c.693delC | p.A232Lfs*20 | NM_000183.3 | c.881C>G | p.P294R | NM_000183.3 |

**Supplemental Table 3.** Control dermal fibroblast cell lines obtained from apparently healthy individuals used in this study

| Cell line | Sex | Age (at sampling) | Origin | Skin source | Race/Ethnicity | Karyotype |
|---|---|---|---|---|---|---|
| FB826 | F | 40 yr. | ATCC | Abdominal | White | Unspecified |
| FB902 | M | 22 yr. | Coriell | Unspecified | White | Unspecified |
| FB554 | M | 8 yr. | Coriell | Inguinal | White | 46,XY |
| FB549 | M | 3 yr. | Coriell | Inguinal | Hispanic/Latino | 46,XY |
| FB550 | F | 19 yr. | Coriell | Unspecified | Unspecified | 46,XX; 2% of cells show random chromosome loss/gain |
| FB552 | F | 9 yr. | Coriell | Unspecified | Black/African American | 46,XX; 2% of cells show random chromosome loss and 4% show random chromosomal aberrations |

**Supplemental Table 4.** Pathogenicity and allele frequency of the variants found in TFP/LCHAD-deficient fibroblasts included in this study

| Gene affected | Reference sequence | DNA change | Protein effect | Type | Gene region | Protein domain | Biological effect | Pathogenicity [a] | Allele frequency [b] |
|---|---|---|---|---|---|---|---|---|---|
| HADHA | NG_007121.1 | c.919-2A>G | — | Splicing | Intron 9 | — | Splice acceptor loss | Pathogenic/ Likely pathogenic | 0.00003890 |
| HADHA | NG_007121.1 | c.2146+1G>A | — | Splicing | Intron 19 | — | Splice donor loss | Pathogenic/ Likely pathogenic | — |
| HADHA | NM_000182.5 | c.1528G>C | p.E510Q | Missense | Exon 15 | Long-chain 3-hydroxyacyl-CoA dehydrogenase (LCHAD) catalytic site | LCHAD activity loss | Pathogenic | 0.001294 |
| HADHA | NM_000182.5 | c.703C>T | p.R235W | Missense | Exon 8 | Long-chain 2,3-enoyl-CoA hydratase (LCEH) | Destabilization of $\alpha$ and $\beta$ subunits interaction | Likely pathogenic | — |
| HADHA | NM_000182.5 | c.403A>G | p.K135E | Missense | Exon 5 | Long-chain 2,3-enoyl-CoA hydratase (LCEH) | Possibly a change in protein folding that may be tolerated. | Likely pathogenic/ Uncertain significance | — |
| HADHB | NM_000183.3 | c.1165A>G | p.N389D | Missense | Exon 14 | Long-chain 3-ketoacyl-CoA thiolase activity (LKAT) | Reduces thiolase activity by increasing the distance between the catalytic site residues $\beta$C458, $\beta$C138 and $\beta$H428 | Not previously reported in ClinVar | 0.000006569 |
| HADHB | NM_000183.3 | c.1289T>C | p.F430S | Missense | Exon 15 | Long-chain 3-ketoacyl-CoA thiolase activity (LKAT) | Interference with a stabilizing pocket near the catalytic site, affecting substrate binding and thiolase function | Conflicting interpretations of pathogenicity: likely pathogenic; uncertain significance | 0.00001768 |
| HADHB | NM_000183.3 | c.693delC | p.A232Lfs*20 | Frameshift deletion | Exon 9 | Long-chain 3-ketoacyl-CoA thiolase activity (LKAT) | Protein truncation or nonsense-mediated mRNA decay | Not previously reported in ClinVar | — |
| HADHB | NM_000183.3 | c.881C>G | p.P294R | Missense | Exon 10 | Long-chain 3-ketoacyl-CoA thiolase activity (LKAT) | Loss of conformational rigidity that affects tertiary structure and protein stability | Likely pathogenic | — |

[a] ClinVar

[b] The Genome Aggregation Database (gnomAD)

**Supplemental Table 5.** Assessment of pathogenicity of variants found in this study by *in silico* predictive tools

| Gene affected | DNA change | Protein effect | Mutation Taster prediction [a] | Provean score and prediction [b] | SIFT score and prediction [c] | Polyphen-2 score and prediction [d] |
|---|---|---|---|---|---|---|
| HADHA | c.919-2A>G | — | Disease causing; 1 | — | — | — |
| HADHA | c.2146+1G>A | — | Disease causing; 1 | — | — | — |
| HADHA | c.1528G>C | p.E510Q | Disease causing; 0.9999999998 | -2.853; deleterious | 0.00; deleterious | 1.000; probably damaging |
| HADHA | c.703C>T | p.R235W | Disease causing; 0.9999988954 | -5.437; deleterious | 0.00; deleterious | 1.000; probably damaging |
| HADHA | c.403A>G | p.K135E | Disease causing; 0.9999998388 | -3.750; deleterious | 0.00; deleterious | 0.998; probably damaging |
| HADHB | c.1165A>G | p.N389D | Disease causing; 0.9999999981 | -4.402; deleterious | 0.01; deleterious | 1.000; probably damaging |
| HADHB | c.1289T>C | p.F430S | Disease causing; 0.9999999999 | -7.888; deleterious | 0.00; deleterious | 1.000; probably damaging |
| HADHB | c.693delC | p.A232Lfs*20 | Disease causing; 1 | — | — | — |
| HADHB | c.881C>G | p.P294R | Disease causing; 0.9999999995 | -7.871; deleterious | 0.00; deleterious | 1.000; probably damaging |

[a] Prediction and probability of the prediction, i.e. a value close to 1 indicates a high “security” of the prediction.

[b] Provean predictions are based on protein structure and function; variants with a score equal to or below -2.5 are considered “deleterious”.

[c] SIFT predictions are based on evolutionary conservation (sequence homology) and the physical properties of amino acids; amino acids with probabilities < 0.05 are predicted to be deleterious.

[d] Polyphen-2 predictions are based on evolutionary conservation (sequence homology) and protein structure and function. The HumDiv-trained Polyphen-2 model was chosen.

**Supplemental Table 6.** Sequences of oligonucleotide primers used in genotype confirmation for exon and adjacent intronic region amplification.

| *HADHA* Fragment | *HADHA* Exons | *HADHA* position cDNA | *HADHA* Primer set | Size, bp | *HADHA* sequence |
|---|---|---|---|---|---|
| A | 1+NC | 1-179 | Ex1-450HADHAfor | ± 734 | [-21M13]-AAGTGGAATCTCGCCTTTGG |
| A | | | In1MTPa rev | | [M13rev]-GGAAGTGACAGTCTCTTCAG |
| B | 2,3 | 68-180 | In1MTPa for | ± 520 | [-21M13]-GCAGTAGTCATATGGCCTAG |
| B | | | In3MTPa rev | | [M13rev]-CCCTTGGCAATCTGTCAAAG |
| C | 4 | 181-314 | In3HADHAfor | ± 530 | [-21M13]-AAGCTACTGACTTGCTGGGA |
| C | | | In4HADHArev | | [M13rev]-GCTCTTGGCCTCATGTGATCC |
| D | 5 | 315-453 | In4MTPa for | ± 570 | [-21M13]-GGTCTCATGGATCCAGAAGT |
| D | | | In5MTPa rev | | [M13rev]-GTGATGCACACCTGTAATCC |
| E | 6 | 454-573 | In5MTPII for | ± 540 | [-21M13]-GCTTCTCTAGCTTTCAGTAG |
| E | | | In6+131HADHArev | | [M13rev]-GTCACTGATCCTGTACACTC |
| F | 7 | 574-676 | In6MTPa for3 | ± 490 | [-21M13]-GCATAAACATATGGCTTGACTT |
| F | | | In7+232HADHArev | | [M13rev]-TGGAACTACAGGTTTGCACC |
| G1 | 8 | 677-799 | In7MTPa for | ± 425 | [-21M13]-CAGTCTCCAGTCTGAAATGG |
| G1 | | | In8+144HADHArev | | [M13rev]-GAGTTGTTACATCTCCTACC |
| G2 | 9 | 800-918 | In8+125HADHAfor | ± 593 | [-21M13]-GGTAGGAGATGTAACAACTC |
| G2 | | | In9MTP rev | | [M13rev]-GGCAATAAGGAGGAGTGATC |
| H | 10 | 919-975 | In9MTPa for | ± 640 | [-21M13]-CATGCATCTGAGGGAGAAAG |
| H | | | In10MTPa rev | | [M13rev]-CACATGCTGGTCTTGAACTC |
| I | 11 | 976-1085 | In10MTPa for | ± 500 | [-21M13]-CAGTGAGAGACAGACTTCTG |
| I | | | In11MTPa rev | | [M13rev]-CTATAGGAATGACCAGAGGG |
| J | 12 | 1086-1220 | In11MTPa for | ± 410 | [-21M13]-CACCTTATAGTTGTGCCTGC |
| J | | | In12MTPa rev | | [M13rev]-TGGCTTCACTACGGAGTATC |
| K | 13 | 1221-1392 | In12-141HADHAfor | ± 392 | [-21M13]-GAAAGTCTCCCTCAAAATGG |
| K | | | In13+43HADHArev | | [M13rev]-TGTTCACTACACTAGGATTC |
| L | 14 | 1393-1479 | In13-82HADHAfor | ± 380 | [-21M13]-GGTGTCACTCATTAGCTTTG |
| L | | | In14MTPa rev | | [M13rev]-CTTACTCCTGCATCTCACAC |
| M1 | 15 | 1480-1620 | In14-152HADHAfor | ± 490 | [-21M13]-AAGACCCATGGAACCAAACC |
| M1 | | | In15+164HADHArev | | [M13rev]-CAGTGGGACAGTCAATACCA |
| M2 | 16 | 1621-1689 | In15+149HADHAfor | ± 580 | [-21M13]-ATTGACTGTCCCACTGAGTC |
| M2 | | | In16MTPa rev | | [M13rev]-CAGTATAAGCCCAACTTCCG |
| N | 17 | 1690-1885 | In16MTPa for | ± 730 | [-21M13]-GTTGGTCATGACACACTCAG |
| N | | | In17+179HADHAarev | | [M13rev]-AGACCATAAGTGGTTGCTAC |
| O | 18 | 1886-2000 | In17MTPa for | ± 670 | [-21M13]-CAGTGGCATAATCTCGTCTC |

| | | | | | |
|---|---|---|---|---|---|
| O | | | In18MTPa rev | | [M13rev]-CTGGTTCTCACCTAGATGCTG |
| P | 19,20 | 2001-2292 | In18HADHAfor | ± 570 | [-21M13]-GACTTCCATTCTGCATCTGC |
| P | | | In20MTP rev | | [M13rev]-TAGACACCACTCTGTTGGAG |

| ***HADHB*** **Fragment** | ***HADHB*** **Exons** | ***HADHB*** **position cDNA** | ***HADHB*** **Primer set** | **Size, bp** | ***HADHB*** **sequence** |
|---|---|---|---|---|---|
| NC | 1 | -251_-9 | ex1-456HADHBfor | ± 605 | [-21M13]-GATCCTGAAGGCAGAAAAGC |
| NC | | | in1+121HADHBrev | | [M13rev]-TCAACTCCCACTTTCCCAGG |
| A | 2,3 | 1-112 | HADHBbin1-173for | ± 650 | [-21M13]-ACCATGTTGGTGTTCCTATC |
| A | | | MTPb in3 rev | | [M13rev]-CCCATTAACATCCTCAAGCC |
| B | 4 | 113-212 | MTPb in3 for | ± 530 | [-21M13]-CAGGAGAATTGCTTGAATC |
| B | | | MTPb in4 rev | | [M13rev]-AAGCTATCTGGACCTCTAGG |
| C | 5 | 213-257 | MTPb in4 for | ± 420 | [-21M13]-GGAGTTAAGGCTTGCTAGAC |
| C | | | MTPb in5rev | | [M13rev]-CTAAGACACTGTTAGGCCTG |
| D | 6 | 258-357 | In5 MTPb for | ± 560 | [-21M13]-GAGAAGGTGCCAAATGCTTG |
| D | | | In6 MTPb rev | | [M13rev]-AGACAATGTCCTAAACCAGC |
| E | 7 | 358-445 | MTPb in6 for | ± 510 | [-21M13]-GTATCATAGCCTCGTGTCTG |
| E | | | MTPb in7 rev | | [M13rev]-CTTGAGCTCAAGAGTTTGAGC |
| F1 | 8 | 446-633 | MTPb in7 for | ± 670 | [-21M13]-GAAGTTGACGTCCATATGGC |
| F1 | | | In8-123HDHBrev | | [M13rev]-CAAGACAAGCAGAGGATCTC |
| F2 | 9 | 634-814 | IN8FORWHADHB | ± 470 | [-21M13]-GCTTGTCTTGGACTTGATTGA |
| F2 | | | IN9REVHADHB | | [M13rev]-GGCTGAATACTTTGGAAACAG |
| G | 10 | 815-936 | MTPb in9 for | ± 600 | [-21M13]-CCTGAATCGTCTGACTTCTG |
| G | | | MTPb in10 rev | | [M13rev]-GCTGGATTAGGTGACTTCTC |
| H | 11,12 | 937-1064 | In10HADHBfor | ± 650 | [-21M13]-TTTGAACAGCCTTCTCTACC |
| H | | | In12HADHBrev2 | | [M13rev]-ACCAATTATGAACTCAGGGC |
| I | 13 | 1065-1152 | MTPb in12 for | ± 270 | [-21M13]-CAGTTTGGGGAATATGAAGG |
| I | | | MTPb in13 rev | | [M13rev]-ACTCCTGCTCTACTCACATC |
| J | 14 | 1153-1227 | In13-143HADHBfor | ± 520 | [-21M13]-TACAGATGTGAGCCACTGTG |
| J | | | MTPb in14 rev | | [M13rev]-CATCTAATGTCAGAATGCAAG |
| K | 15 | 1228-1392 | MTPb in14 for | ± 530 | [-21M13]-CTTGCATTCTGACATTAGATG |
| K | | | In15HADHBrev | | [M13rev]-CAGAATTACAGCTGTTATCC |
| L | 16 | 1393-1428 | MTPb in15 for | ± 270 | [-21M13]-CAGTGATTGATTGGCAGAGC |
| L | | | MTPb ex16 rev | | [M13rev]-TCCTAAGAGGAGCTAGGAAC |

| | |
|---|---|
| -21M13 | TGTAAAACGACGGCCAGT |
| M13rev | CAGGAAACAGCTATGACC |

**Supplemental Table 7.** Sequence of oligonucleotide primers and probes used in *HADHA* and *HADHB* ddPCR variant detection from cDNA and genomic DNA.

| Oligo Name | Sequence (5′ to 3′) |
|---|---|
| Hs_HADHAc403_F | GCTATCACAAGAAGCACAGAGA |
| Hs_HADHAc403_R1 | CATGAAATGGCAACCTCAAGTC |
| Hs_HADHAc403_R2 | GCCACCAATGAGTTGGACAGT |
| Hs_HADHAc403A_pr | /56-FAM/CCACAATAG/ZEN/GCTTTGTGGACTTTTC/3IABkFQ/ |
| Hs_HADHAc403G_pr | /56-FAM/CCACAATAG/ZEN/GCTCTGTGGACTTTTC/3IABkFQ/ |
| Hs_HADHAc1528_F1 | GACCTGAGAAGGTGATTGGCA |
| Hs_HADHAc1528_F2 | CAGGTGATTGGCATGCACTAC |
| Hs_HADHAc1528_R | CTGAAGCACTGGTGTCTTTGG |
| Hs_HADHAc1528G_pr | /56-FAM/ATGCAGCTG/ZEN/CTGGAGATTATCACGA/3IABkFQ |
| Hs_HADHAc1528C_pr | /56-FAM/ATGCAGCTG/ZEN/CTGCAGATTATCACGA/3IABkFQ/ |
| Hs_HADHBc881_F1 | GTACCCTTCAAAGTACCAGGAA |
| Hs_HADHBc881_F2 | ACCAAAGATAATGGCATCCGTC |
| Hs_HADHBc881_R | CAGCTGTCACTGTGCCGTAG |
| Hs_HADHBc881C_pr | /56-FAM/CCAAACTAA/ZEN/AACCTGCATTCATCAAG/3IABkFQ/ |
| Hs_HADHBc881G_pr | /56-FAM/CCAAACTAA/ZEN/AACGTGCATTCATCAAG/3IABkFQ/ |
| GAPDH_ex7_fwd | TGGCATTGCCCTCAACGACC |
| GAPDH_ex8_rev | TACTCCTTGGAGGCCATGTGG |
| GAPDH_probe | /5HEX/CTACAGCAA/ZEN/CAGGGTGGTGGAC/3IABkFQ/ |
| Hs_RPP30_in1_fwd | TTCCTAGCGCGGGAAACTCG |
| Hs_RPP30_in1_rev | TGCAAATCCCTCGCCCTCGT |
| Hs_RPP30_in1_HEX | /5HEX/TCCTGCAAT/ZEN/GAGGGAACTGAGGC/3IABkFQ/ |

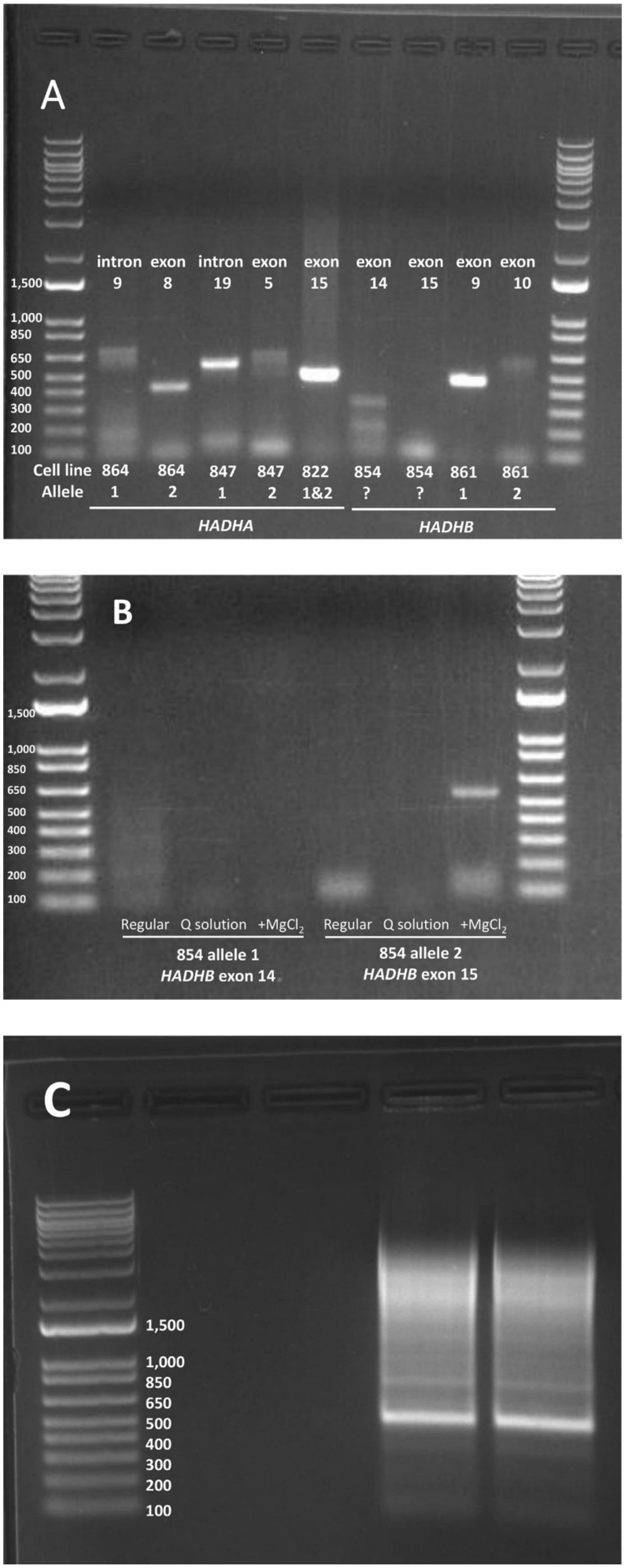


Supplemental Figure 1. PCR amplification of all regions of interest for genotype confirmation. (A) PCR amplification with Promega PCR Master Mix was successful for all regions of interest, except exons 14 and 15 of HADHB for cell line 854. (B) Amplification of exon 15 of HADHB with Qiagen Taq DNA polymerase with added Q solution, and extra MgCl2. (C) Amplification of exon 14 of HADHB from cell line 854 was successful with Invitrogen AccuPrime™ High-fidelity Pfx DNA Polymerase.

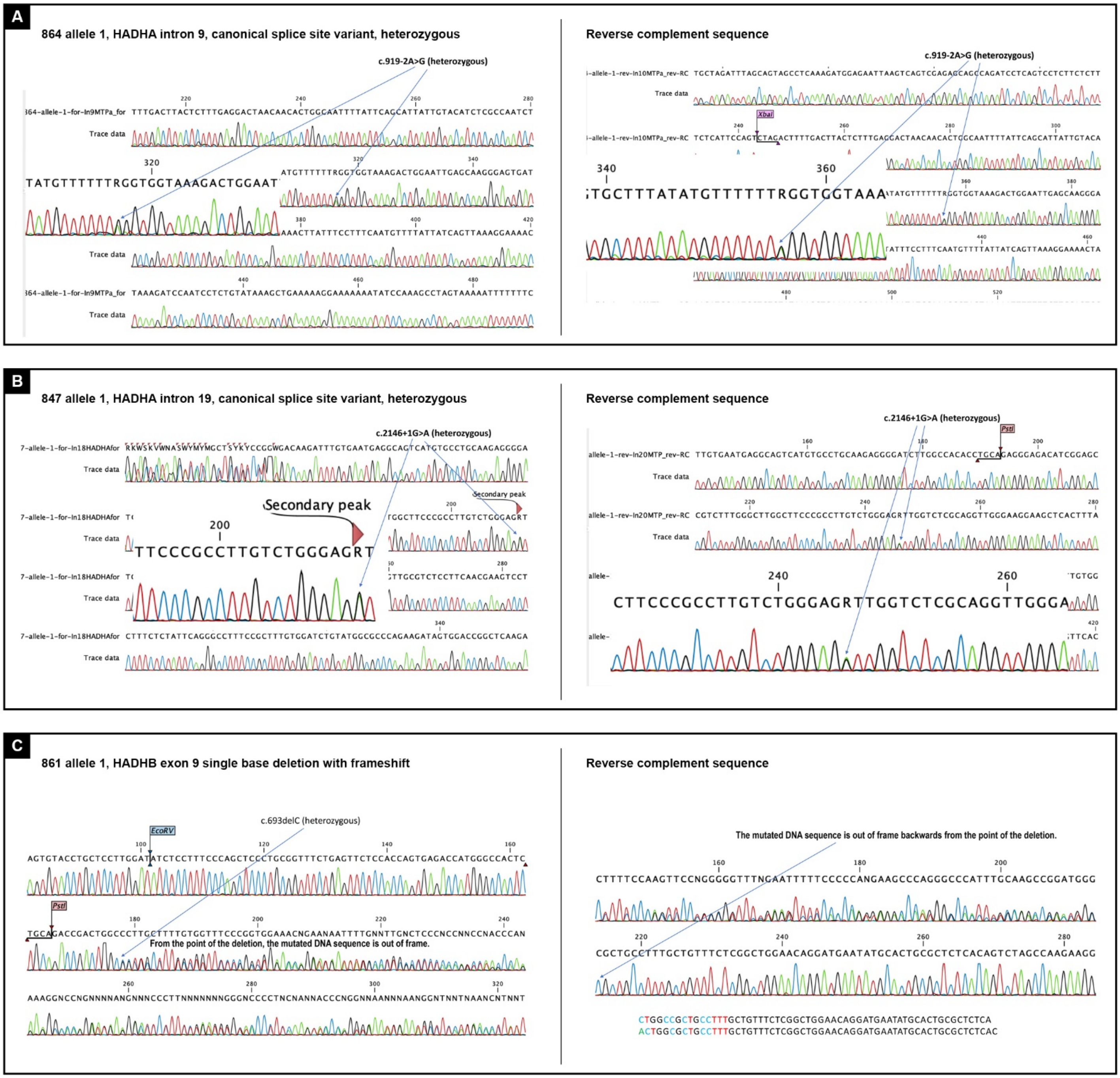


*Supplemental Figure 2. Sanger sequencing confirmation of three truncating variants found in patients of this study. (A) HADHA intron 9 canonical splice site variant, heterozygous; (B) HADHA intron 19 canonical splice site variant, heterozygous; (C) Single base HADHB deletion in exon 9 leading to a frameshift.*

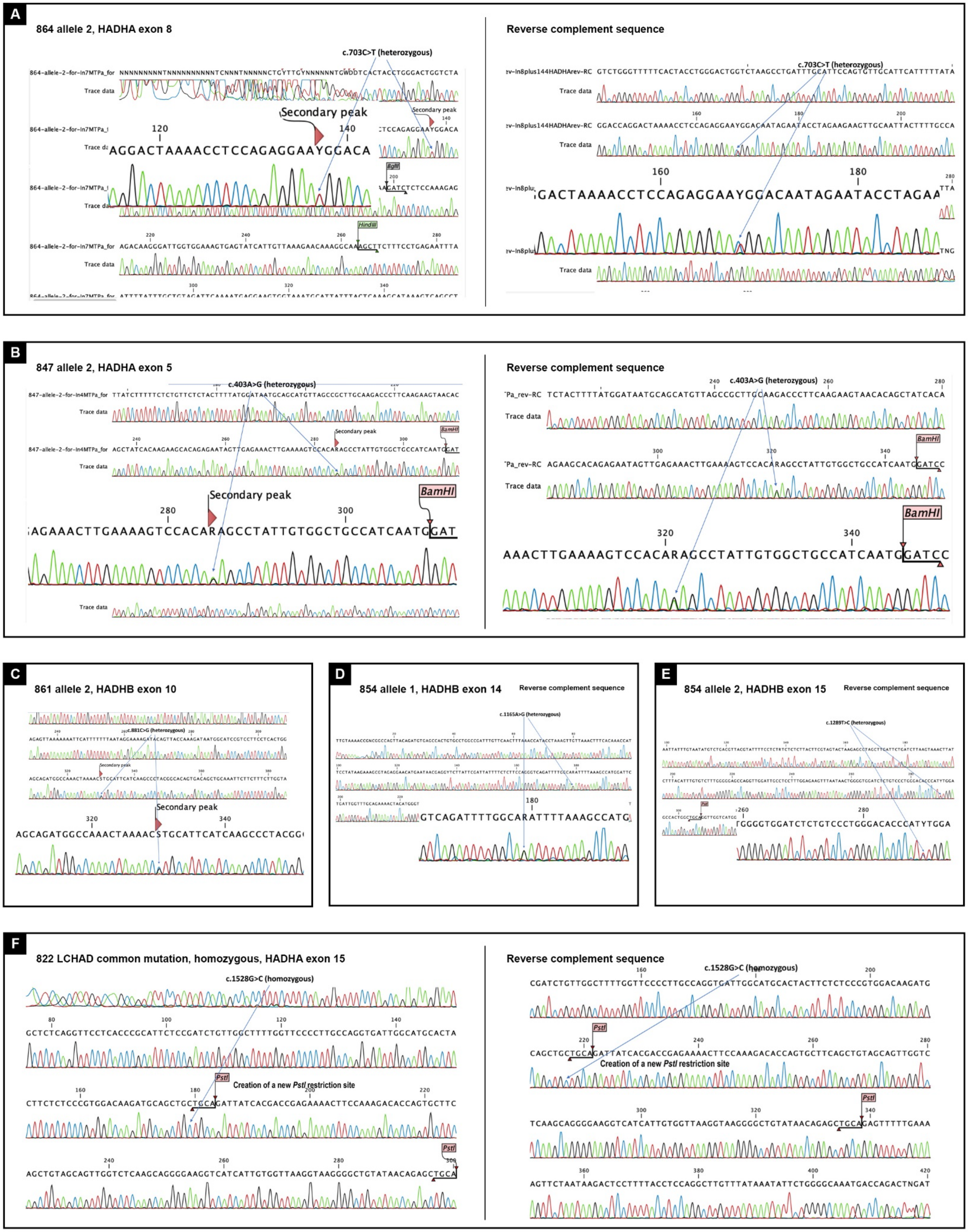


*Supplemental Figure 3. Sanger sequencing confirmation of missense variants found in patients of this study. (A) HADHA exon 8, c.703C>T, heterozygous; (B) HADHA exon 5, c.403A>G, heterozygous; (C) HADHB exon 10, c.881C>G, heterozygous; (D) HADHB exon 14, c.1165A>G, heterozygous; (E) HADHB exon 15, c.1289T>C, heterozygous; (F) HADHA exon 15, c.1528G>C, homozygous.*

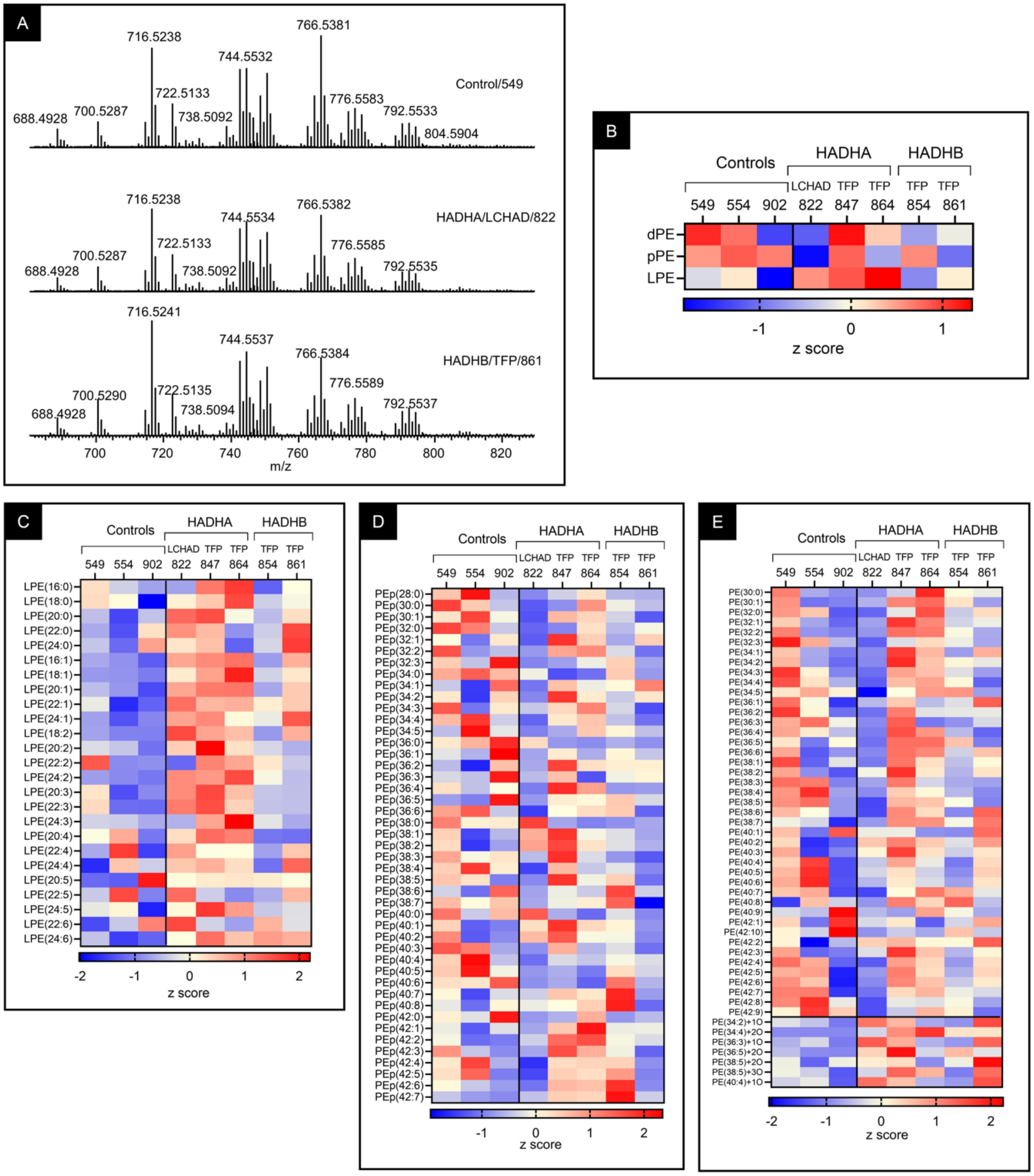


*Supplemental Figure 4. LC-MS/MS assessment of phosphatidylethanolamine (PE) species in human fibroblasts. Typical mass spectra of phosphatidylethanolamine (PE) obtained from control (FB549) and TFP/LCHAD-deficient fibroblasts (FB822 and FB861) (A). Diacyl-PE (dPE, PE) had significant variation among controls, hindering the interpretation of the patterns found in TFP/LCHAD-deficient fibroblasts (B&E). Lyso-PE (LPE) and oxidized PE had clearly increased levels in TFP/LCHAD-deficient fibroblasts (B,C&E). FB822 and FB861, the cells with the highest levels of oxidized PE, had the lowest levels of plasmalogen-PE (pPE) (D). Figures 10-15: Data (pmol/nmol of total phospholipids) are presented as heatmaps auto-scaled to z scores as in Figure 8. Each lane represents the mean of three technical repeats.*

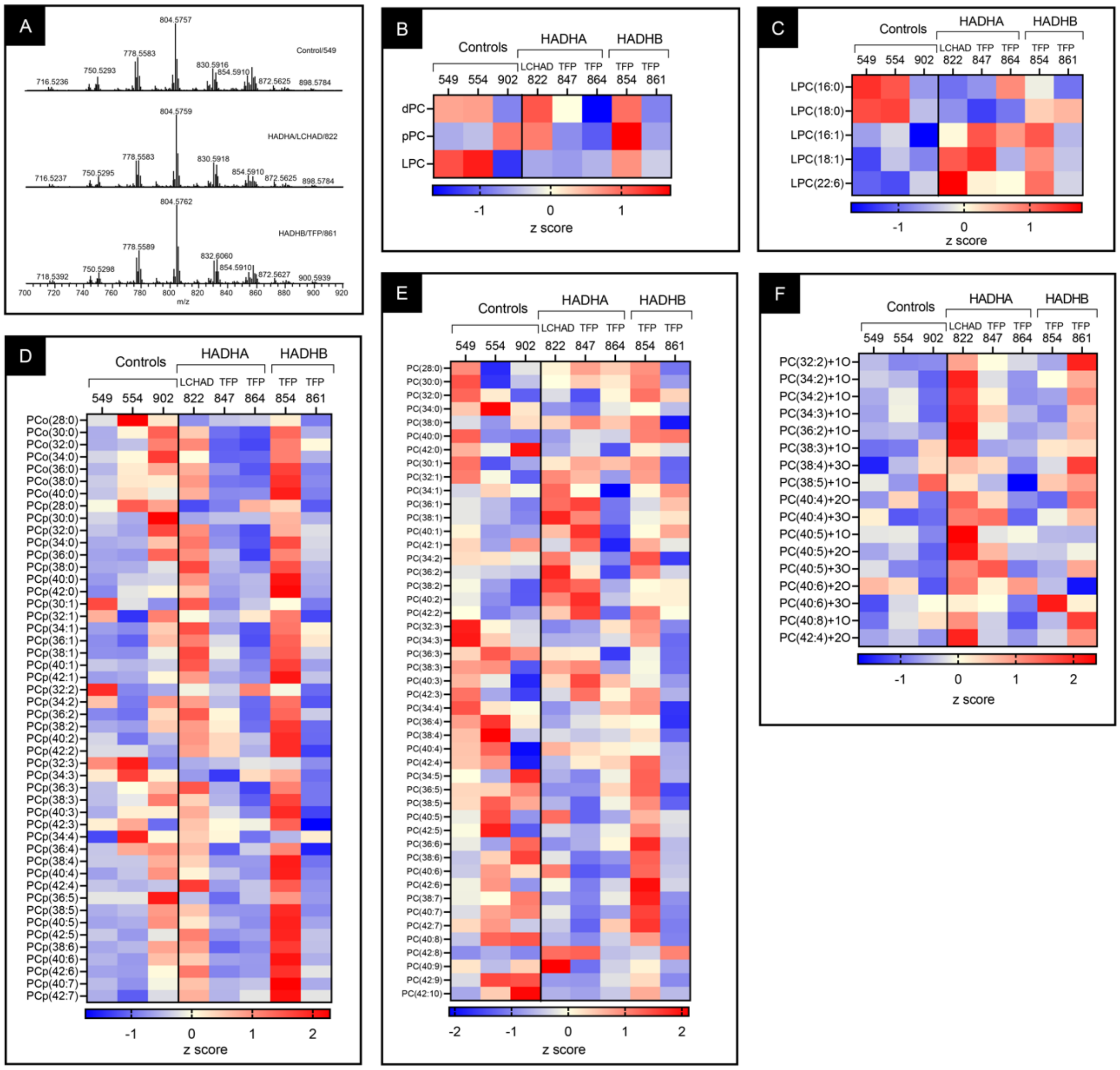

*Supplemental Figure 5. LC-MS/MS assessment of phosphatidylcholine (PC) species in human fibroblasts. Typical mass spectra of PC obtained from control (FB549) and TFP/LCHAD-deficient fibroblasts (FB822 and FB861). Diacyl-PC (dPC, PC), plasmalogen-PC (pPC), and lyso-PC (LPC) had significant variation among controls, hindering the interpretation of the patterns found in TFP/LCHAD-deficient fibroblasts (B-E). However, FB822 and FB854 had increased levels of PC and pPC when compared to the average of controls (B,D&E). Oxidized PC was clearly raised in FB822, FB847, and FB861 (F). Each lane represents the mean of three technical repeats.*

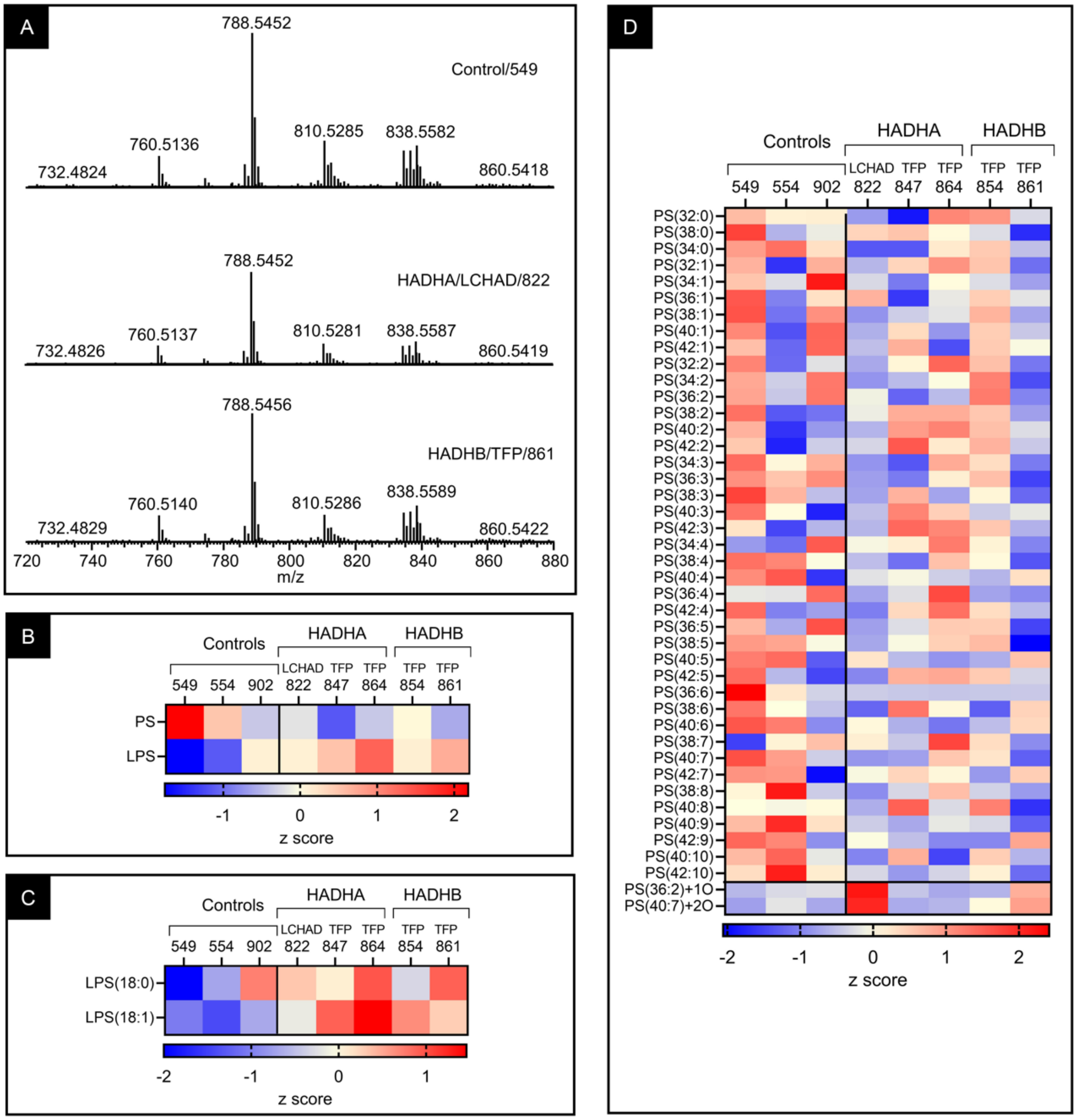


*Supplemental Figure 6. LC-MS/MS assessment of phosphatidylserine (PS) species in human fibroblasts. Typical mass spectra of PS obtained from control (FB549) and TFP/LCHAD-deficient fibroblasts (FB822 and FB861). Phosphatidylserine (PS) levels were decreased in all TFP/LCHAD-deficient fibroblasts, considering the average of the three controls (B&D). An opposite pattern was seen in LPS, which had increased levels in patient-derived fibroblasts compared to controls (B&C). Oxidized PS was clearly raised in FB822 and FB861 (D). Each lane represents the mean of three technical repeats.*

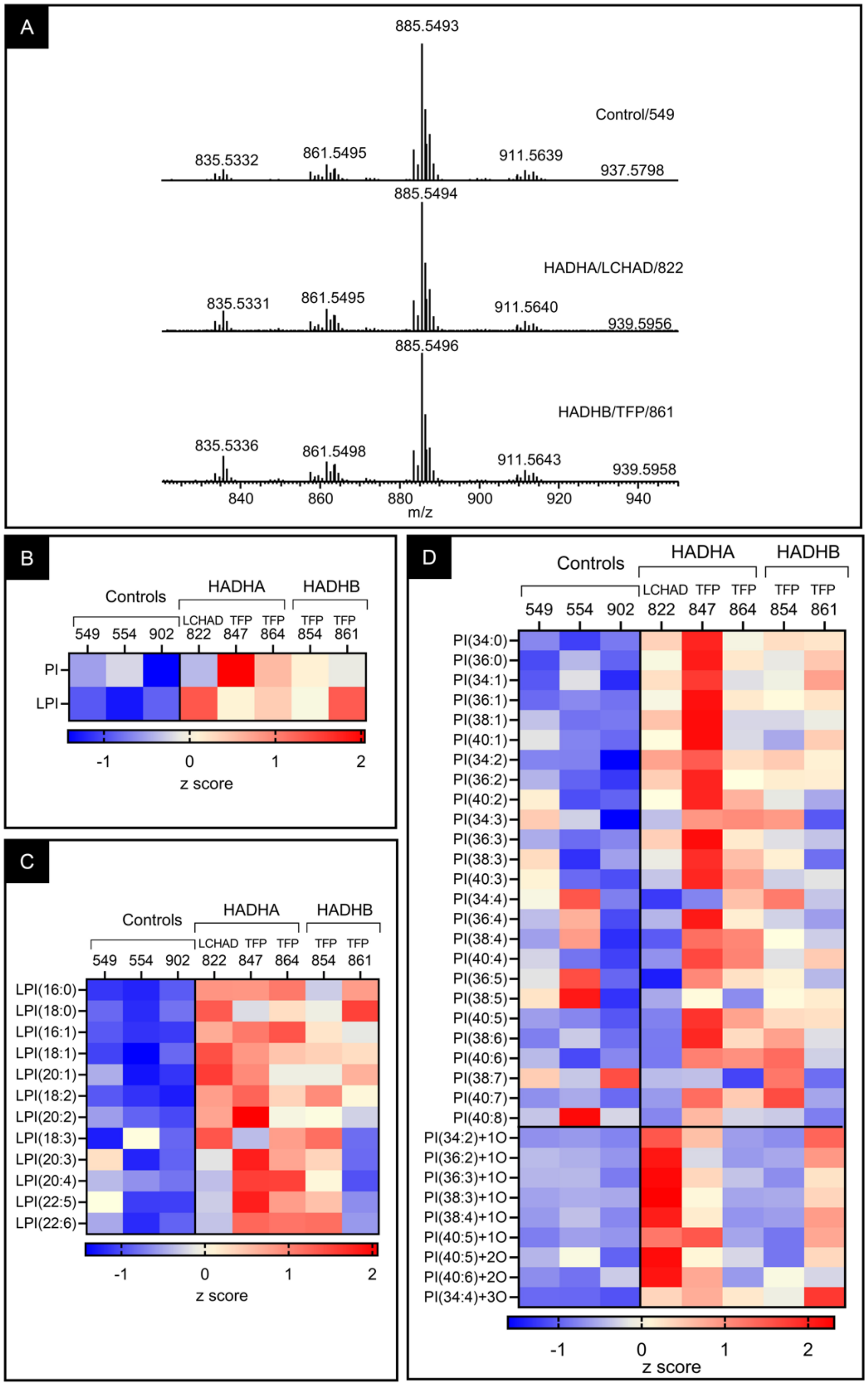


*Supplemental Figure 7. LC-MS/MS assessment of phosphatidylinositol (PI) species in human fibroblasts. Typical mass spectra of PI obtained from control (FB549) and TFP/LCHAD-deficient fibroblasts (FB822 and FB861). Phosphatidylinositol (PI) levels were increased in all TFP/LCHAD-deficient fibroblasts, considering the average of the three controls (B&D). The same pattern was seen in LPI, which had increased levels in patient-derived fibroblasts compared to controls (B&C). Oxidized PI was clearly raised in FB822, FB847, and FB861 (D). Each lane represents the mean of three technical repeats.*

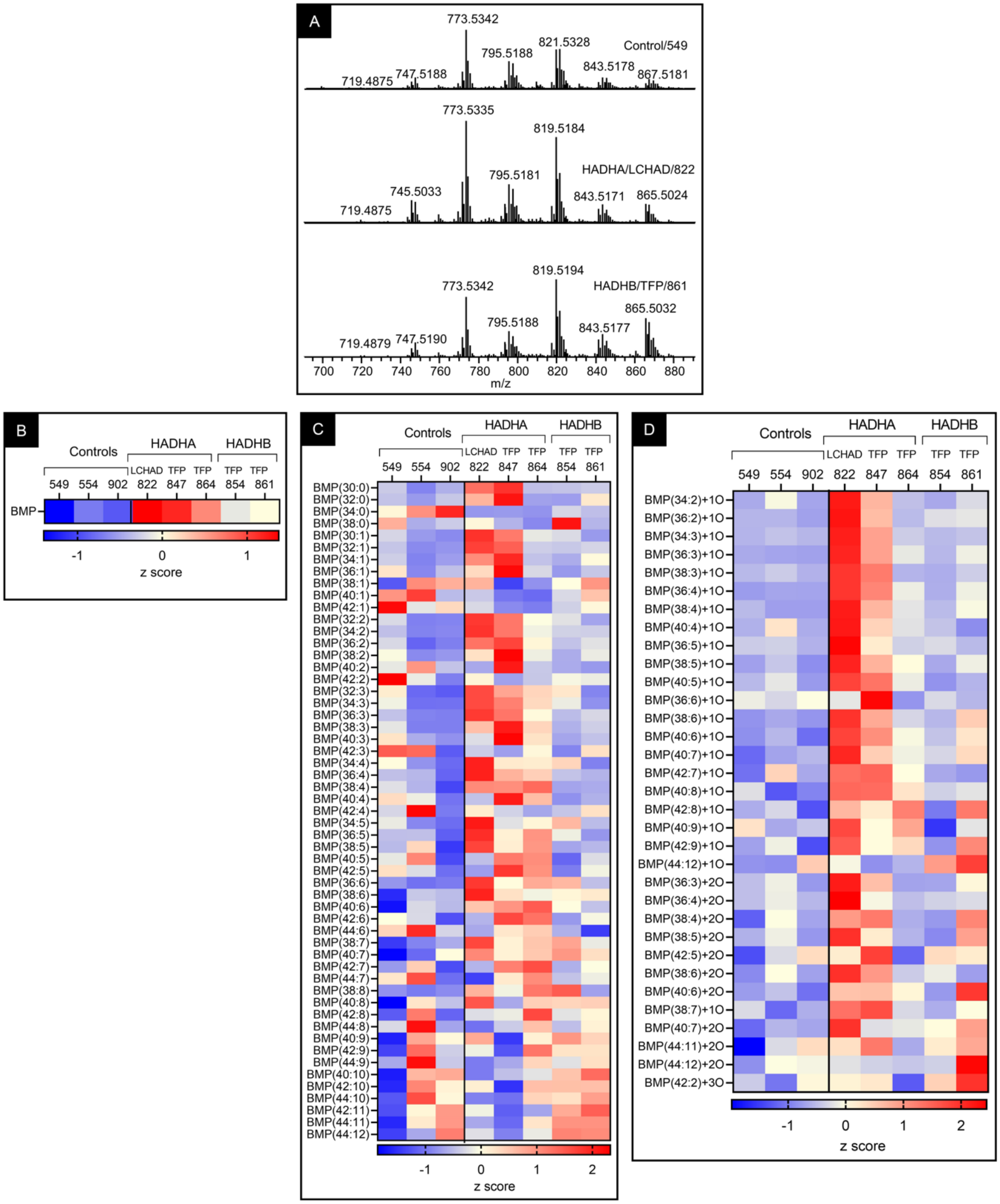


*Supplemental Figure 8. LC-MS/MS assessment of bis-monoacyl-glycerophosphate (BMP) species in human fibroblasts. Typical mass spectra of BMP obtained from control (FB549) and TFP/LCHAD-deficient fibroblasts (FB822 and FB861). BMP levels were increased in all TFP/LCHAD-deficient fibroblasts compared to controls (B&D). The same pattern was seen in LPI, which had increased levels in patient-derived fibroblasts compared to controls (B&C). Oxidized BMP was clearly raised in FB822, FB847, and FB861 (D). Each lane represents the mean of three technical repeats.*

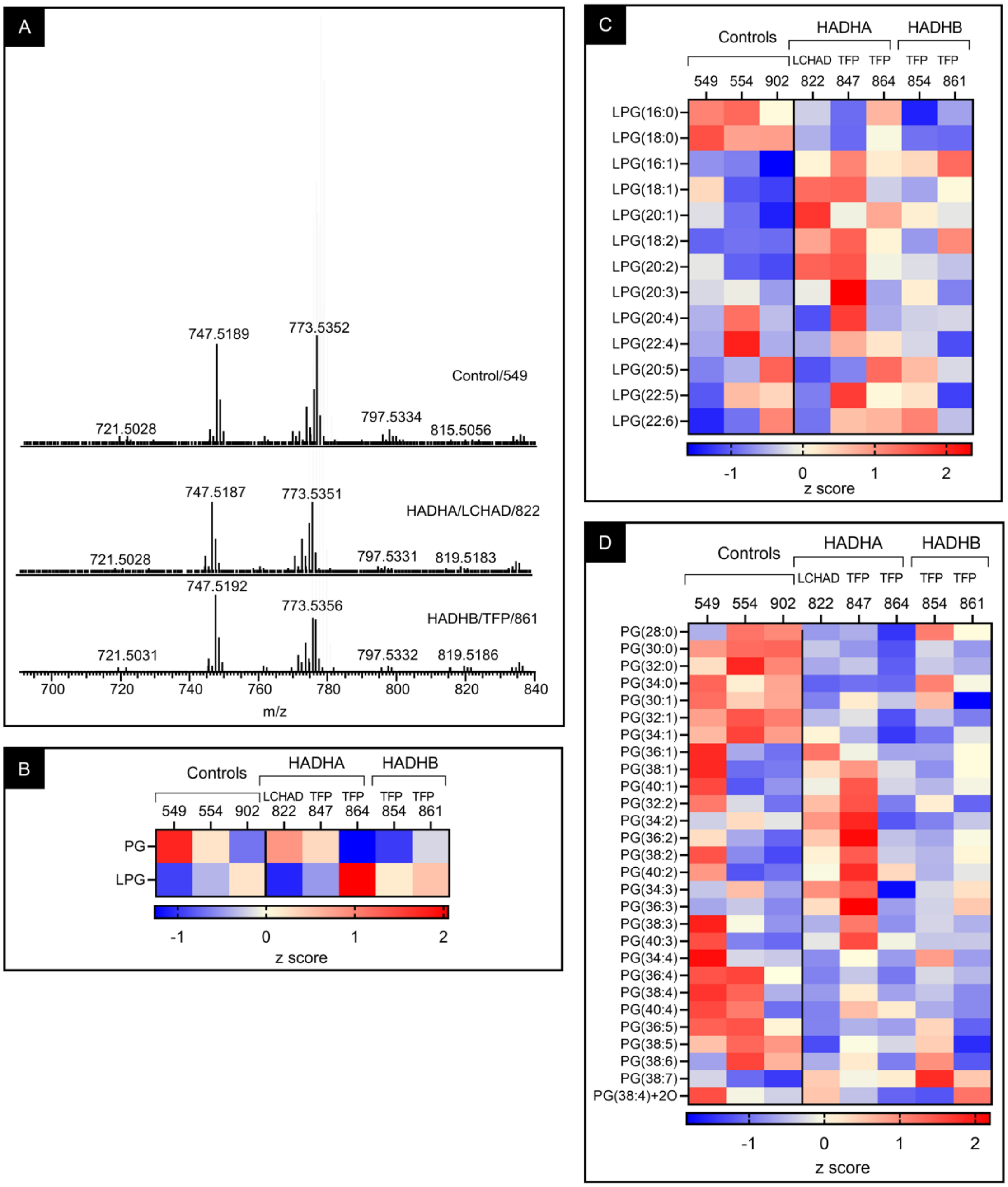


*Supplemental Figure 9. LC-MS/MS assessment of phosphatidylglycerol (PG) species in human fibroblasts. Typical mass spectra of PG obtained from control (FB549) and TFP/LCHAD-deficient fibroblasts (FB822 and FB861). PG and lyso-PG (LPG) levels had significant variation among controls, making it difficult to attribute the patterns found in TFP/LCHAD-deficient fibroblasts to genotype (B-D). Each lane represents the mean of three technical repeats.*

**Supplemental methods**

**Structure analysis and molecular dynamics (MD) simulations of variant proteins**

**Structure data of αTFP p.E510Q**

Structure analysis of the variant protein αTFP p.E510Q was performed on a Silicon Graphics Fuel Workstation (Mountain View, CA, USA) using the Insight II 2000 software package (BIOVIA Dassault Systèmes, San Diego, CA, USA) using the previously published TFP atomic coordinates (6DV2.pdb) (1).

**Structure data of other missense mutations**

The tetrameric form of TFP, comprising of two α (HADHA) and two β (HADHB) subunits were modelled using Swiss-Model server (2). Reference sequences were retrieved from Protein [Internet], National Center for Biotechnology Information (3, 4). Crystallographic structure 6DV2 (1) was used as template. At total, two tetramers containing mutations K135E or R235W in the α subunit and three tetramers containing mutations N389D, F430S, or P294R in the β subunit were obtained. All structures were protonated at pH 7.5 using ProPKA algorithm in the PDB2PQR server (5).

**Molecular dynamics (MD) simulations of missense mutations**

To account for the effect of conformational changes due to mutations, the wild-type and the mutant tetrameric structures of TFP were submitted to 100 ns molecular dynamics simulations each. All MD simulations were performed using GROMACS 2020.6 (6) with the AMBER99SB forcefield (7). Each simulation system consisted of ~ 260,000 atoms that included the TFP tetramer, water molecules, and ions Na+ and Cl− at 150mM concentration. The integration steps of all simulations were set to 2 fs. Simulations were set using Particle Mesh Ewald (PME) method (8). Temperature coupling was done with V-rescale thermostat at 297.15 K, and the Parrinello-Rahman barostat (9). Water molecules were described using TIP3P water model (10). Covalent bonds were constrained to their

equilibrium length by the LINCS algorithm (11). Tetramer stability for all structures was assessed through visual inspection and root-mean-square deviation (RMSD) calculations using the “gmx rms” algorithm.

## References


1. Xia C, Fu Z, Battaile KP, and Kim JP. Crystal structure of human mitochondrial trifunctional protein, a fatty acid β-oxidation metabolon. *Proc Natl Acad Sci U S A.* 2019;116(13):6069-74.
2. Biasini M, Bienert S, Waterhouse A, Arnold K, Studer G, Schmidt T, et al. SWISS-MODEL: modelling protein tertiary and quaternary structure using evolutionary information. *Nucleic Acids Res.* 2014;42(Web Server issue):W252-8.
3. Protein [Internet]. trifunctional enzyme subunit beta, mitochondrial isoform 1 precursor [Homo sapiens]. https://www.ncbi.nlm.nih.gov/protein/NP_000174.1. Updated 3/29 Accessed 6/13, 2023.
4. Protein [Internet]. trifunctional enzyme subunit alpha, mitochondrial precursor [Homo sapiens]. https://www.ncbi.nlm.nih.gov/protein/NP_000173.2. Updated 3/12 Accessed 6/13, 2023.
5. Dolinsky TJ, Czodrowski P, Li H, Nielsen JE, Jensen JH, Klebe G, et al. PDB2PQR: expanding and upgrading automated preparation of biomolecular structures for molecular simulations. *Nucleic Acids Res.* 2007;35(Web Server issue):W522-5.
6. Van Der Spoel D, Lindahl E, Hess B, Groenhof G, Mark AE, and Berendsen HJ. GROMACS: fast, flexible, and free. *J Comput Chem.* 2005;26(16):1701-18.
7. Villavicencio B, Ligabue-Braun R, and Verli H. All-Hydrocarbon Staples and Their Effect over Peptide Conformation under Different Force Fields. *J Chem Inf Model.* 2018;58(9):2015-23.
8. Essmann U, Perera L, Berkowitz ML, Darden T, Lee H, and Pedersen LG. A Smooth Particle Mesh Ewald Method. *J Chem Phys.* 1995;103(19):8577-93.
9. Parrinello M, and Rahman A. Polymorphic Transitions in Single-Crystals - a New Molecular-Dynamics Method. *J Appl Phys.* 1981;52(12):7182-90.
10. Bjelkmar P, Larsson P, Cuendet MA, Hess B, and Lindahl E. Implementation of the CHARMM Force Field in GROMACS: Analysis of Protein Stability Effects from Correction Maps, Virtual Interaction Sites, and Water Models. *J Chem Theory Comput.* 2010;6(2):459-66.
11. Hess B, Bekker H, Berendsen HJC, and Fraaije JGEM. LINCS: A linear constraint solver for molecular simulations. *Journal of Computational Chemistry.* 1997;18(12):1463-72.
