# Supplementary material for: Mitochondrial bioenergetics and cardiolipin remodeling abnormalities in mitochondrial trifunctional protein deficiency
Source: JCI Insight. 2024 Sep 10;9(17):e176887. doi: 10.1172/jci.insight.176887 (PMC11385086; doi:10.1172/jci.insight.176887)

Full unedited gel for Figure 3A
ProteinTech mouse monoclonal anti-HADHA; Abcam rabbit monoclonal anti-TOMM20

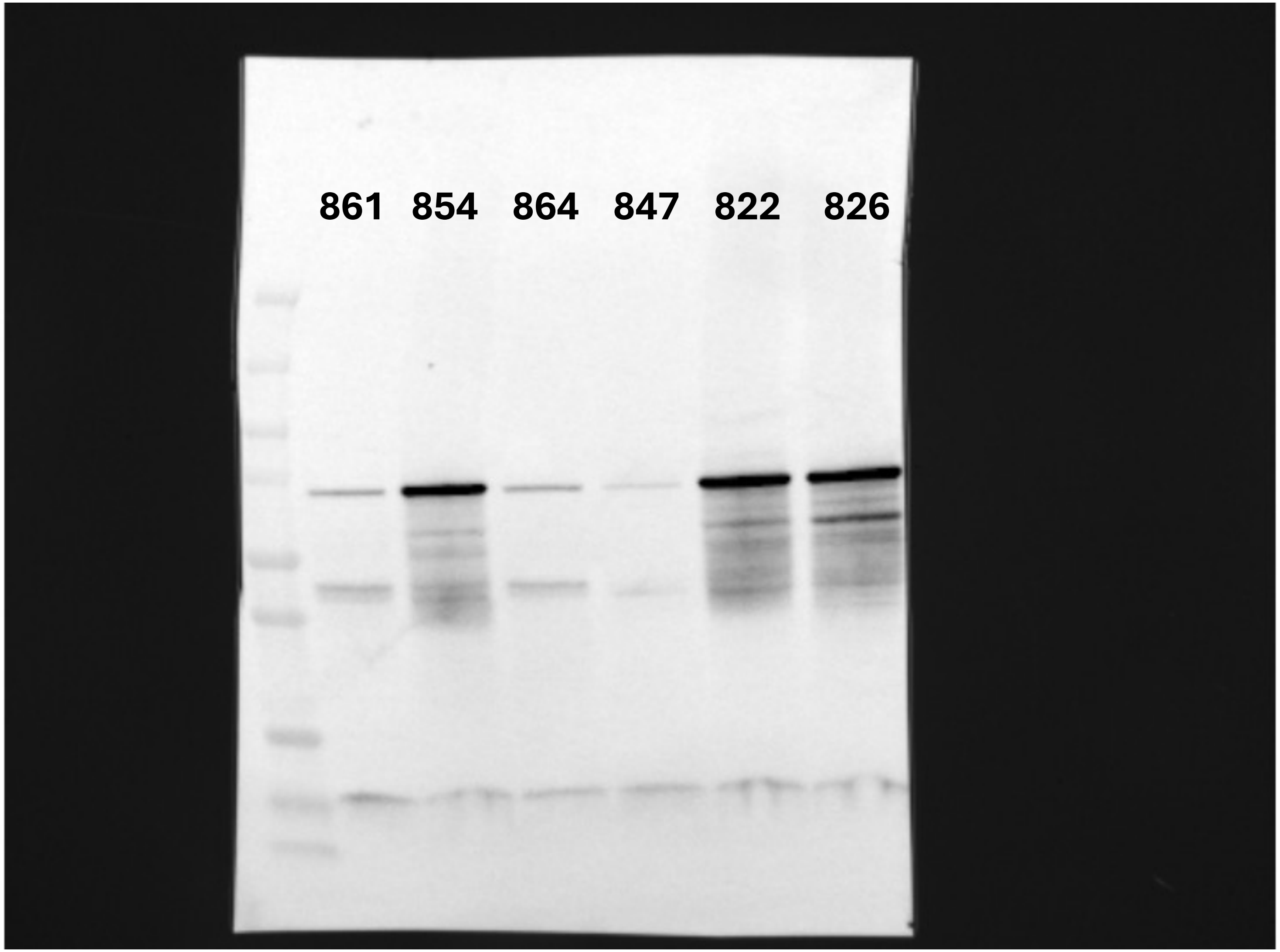

Full unedited gel for Figure 3B
Santa Cruz Biotech mouse monoclonal anti-HADHB; Abcam rabbit monoclonal anti-TOMM20

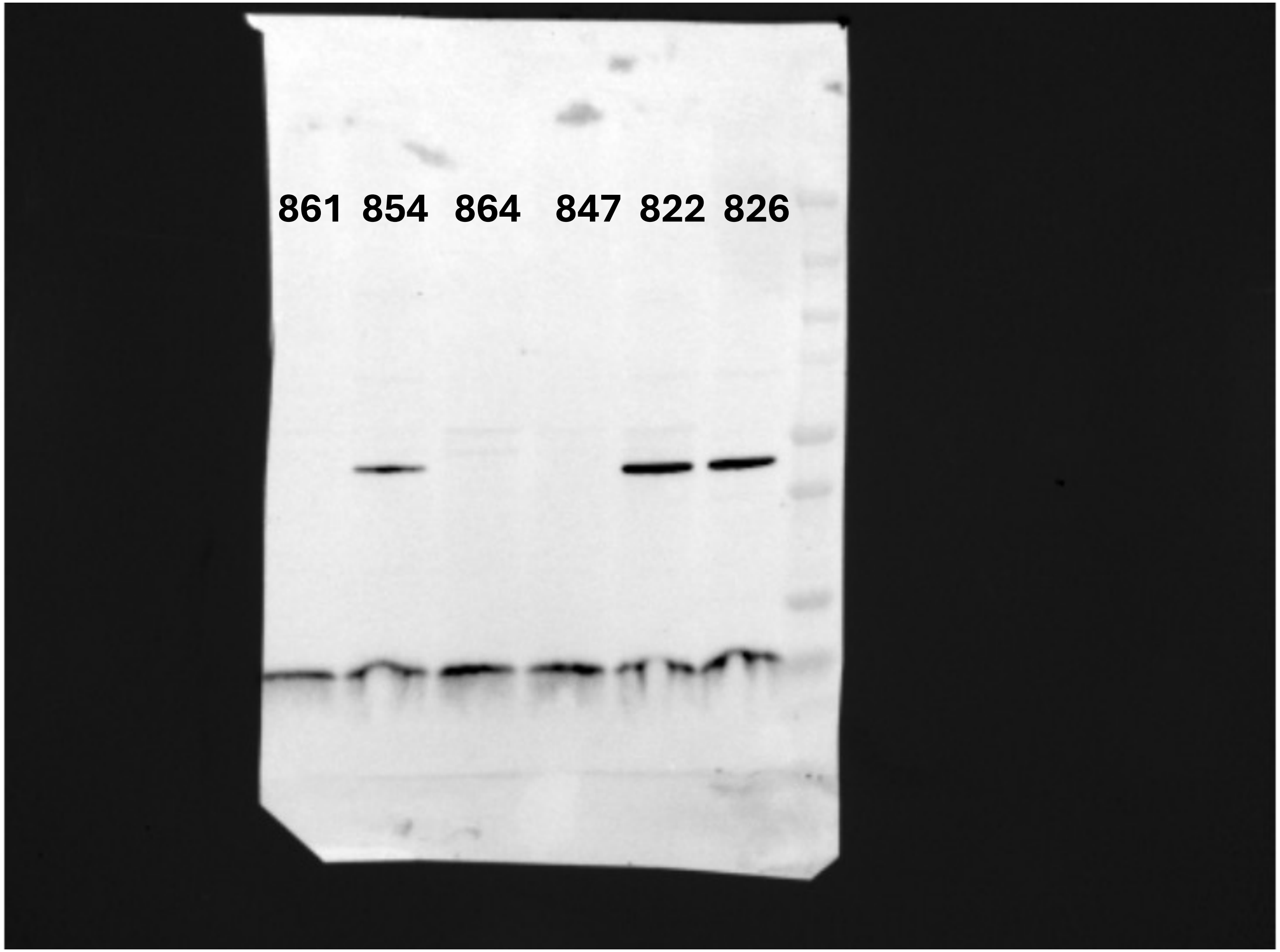

Supplement: Unedited blot and gel images [file jciinsight-9-176887-s090.pdf]
