# Supplementary material for: Corrigendum to: Mitochondrial bioenergetics and cardiolipin remodeling abnormalities in mitochondrial trifunctional protein deficiency
Source: JCI Insight. 2026 Apr 8;11(7):e206548. doi: 10.1172/jci.insight.206548 (PMC13134716; doi:10.1172/jci.insight.206548)
Supplement: Supplemental data [file jciinsight-11-206548-s269.pdf]

Table S1. Clinical characteristics of patients with TFP/LCHAD deficiency included in the study

Patient	Cell line	Sex	Current state	TFP/LCHAD Deficiency	Hypoglycemia	Hypotonia/Motor Delay	Muscle weakness	Rhabdomyolysis	Cardiomyopathy	Peripheral neuropathy	Retinopathy	Maternal HELLP syndrome
1	FB864	F	12y, AFOs	TFP (*HADHA*)	N	N	Y	Y	N	Y	N	N
2	FB847	M	7y	TFP (*HADHA*)	N	N	Y	Y	N	N	N	Y
3	FB822	M	12y	LCHAD	N	N	N	Y	Y (reversed by treatment)	N	N	Y
4	FB944	F	Died 19y	LCHAD	Y	N	Y	Y	Y	Y	Y	N
5	FB942	F	Died 19y	LCHAD	Y	N	Y	Y	N	N	Y	Y
6	FB854	F	Died 4y	TFP (*HADHB*)	Y	Y	Y	Y	Y (severe, dilated/ hypertrophic)	Y	N	N
7	FB861	M	20y, wheelchair-bound	TFP (*HADHB*)	Y	Y	Y	Y	N	Y	N	N

Legend: AFOs, Ankle-Foot Orthoses; HELLP syndrome, hemolysis, elevated liver enzymes, and low platelets syndrome

Table S2. TFP/LCHAD-deficient fibroblast cell lines and their respective genotypes

Patient	Sex	TFP/LCHAD Deficiency	Cell line	Gene Affected	Allele 1	Amino acid change	Reference sequence	Allele 2	Amino acid change	Reference sequence
1	F	TFP	FB864	*HADHA*	c.919-2A>G	—	NG_007121.1	c.703C>T	p.R235W	NM_000182.5
2	M	TFP	FB847	*HADHA*	c.2146+1G>A	—	NG_007121.1	c.403A>G	p.K135E	NM_000182.5
3	M	LCHAD	FB822	*HADHA*	c.1528G>C	p.E510Q	NM_000182.5	c.1528G>C	p.E510Q	NM_000182.5
4	F	LCHAD	FB944	*HADHA*	c.1528G>C	p.E510Q	NM_000182.5	c.1528G>C	p.E510Q	NM_000182.5
5	F	LCHAD	FB942	*HADHA*	c.1528G>C	p.E510Q	NM_000182.5	c.1528G>C	p.E510Q	NM_000182.5
6	F	TFP	FB854	*HADHB*	c.1165A>G	p.N389D	NM_000183.3	c.1289T>C	p.F430S	NM_000183.3
7	M	TFP	FB861	*HADHB*	c.693delC	p.A232Lfs*20	NM_000183.3	c.881C>G	p.P294R	NM_000183.3

Table S3. Control dermal fibroblast cell lines obtained from apparently healthy individuals used in this study

Cell line	Sex	Age (at sampling)	Origin	Skin source	Race/Ethnicity	Karyotype
FB826	F	40 yr.	ATCC	Abdominal	White	Unspecified
FB902	M	22 yr.	Coriell	Unspecified	White	Unspecified
FB554	M	8 yr.	Coriell	Inguinal	White	46,XY
FB549	M	3 yr.	Coriell	Inguinal	Hispanic/Latino	46,XY
FB550	F	19 yr.	Coriell	Unspecified	Unspecified	46,XX; 2% of cells show random chromosome loss/gain
FB552	F	9 yr.	Coriell	Unspecified	Black/African American	46,XX; 2% of cells show random chromosome loss and 4% show random chromosomal aberrations

Table S4. Pathogenicity and allele frequency of the variants found in TFP/LCHAD-deficient fibroblasts included in this study

Gene affected	Reference sequence	DNA change	Protein effect	Type	Gene region	Protein domain	Biological effect	Pathogenicity [a]	Allele frequency [b]
HADHA	NG_007121.1	c.919-2A>G	—	Splicing	Intron 9	—	Splice acceptor loss	Pathogenic/ Likely pathogenic	0.00003890
HADHA	NG_007121.1	c.2146+1G>A	—	Splicing	Intron 19	—	Splice donor loss	Pathogenic/ Likely pathogenic	—
HADHA	NM_000182.5	c.1528G>C	p.E510Q	Missense	Exon 15	Long-chain 3-hydroxyacyl-CoA dehydrogenase (LCHAD) catalytic site	LCHAD activity loss	Pathogenic	0.001294
HADHA	NM_000182.5	c.703C>T	p.R235W	Missense	Exon 8	Long-chain 2,3-enoyl-CoA hydratase (LCEH)	Destabilization of α and β subunits interaction	Likely pathogenic	—
HADHA	NM_000182.5	c.403A>G	p.K135E	Missense	Exon 5	Long-chain 2,3-enoyl-CoA hydratase (LCEH)	Possibly a change in protein folding that may be tolerated.	Likely pathogenic/ Uncertain significance	—
HADHB	NM_000183.3	c.1165A>G	p.N389D	Missense	Exon 14	Long-chain 3-ketoacyl-CoA thiolase activity (LKAT)	Reduces thiolase activity by increasing the distance between the catalytic site residues βC458, βC138 and βH428	Not previously reported in ClinVar	0.000006569
HADHB	NM_000183.3	c.1289T>C	p.F430S	Missense	Exon 15	Long-chain 3-ketoacyl-CoA thiolase activity (LKAT)	Interference with a stabilizing pocket near the catalytic site, affecting substrate binding and thiolase function	Conflicting interpretations of pathogenicity: likely pathogenic; uncertain significance	0.00001768
HADHB	NM_000183.3	c.693delC	p.A232Lfs*20	Frameshift deletion	Exon 9	Long-chain 3-ketoacyl-CoA thiolase activity (LKAT)	Protein truncation or nonsense-mediated mRNA decay	Not previously reported in ClinVar	—
HADHB	NM_000183.3	c.881C>G	p.P294R	Missense	Exon 10	Long-chain 3-ketoacyl-CoA thiolase activity (LKAT)	Loss of conformational rigidity that affects tertiary structure and protein stability	Likely pathogenic	—

[a] ClinVar

[b] The Genome Aggregation Database (gnomAD)

Table S5. Assessment of pathogenicity of variants found in this study by *in silico* predictive tools

Gene affected	DNA change	Protein effect	Mutation Taster prediction [a]	Provean score and prediction [b]	SIFT score and prediction [c]	Polyphen-2 score and prediction [d]
HADHA	c.919-2A>G	—	Disease causing; 1	—	—	—
HADHA	c.2146+1G>A	—	Disease causing; 1	—	—	—
HADHA	c.1528G>C	p.E510Q	Disease causing; 0.9999999998	-2.853; deleterious	0.00; deleterious	1.000; probably damaging
HADHA	c.703C>T	p.R235W	Disease causing; 0.9999988954	-5.437; deleterious	0.00; deleterious	1.000; probably damaging
HADHA	c.403A>G	p.K135E	Disease causing; 0.9999998388	-3.750; deleterious	0.00; deleterious	0.998; probably damaging
HADHB	c.1165A>G	p.N389D	Disease causing; 0.9999999981	-4.402; deleterious	0.01; deleterious	1.000; probably damaging
HADHB	c.1289T>C	p.F430S	Disease causing; 0.9999999999	-7.888; deleterious	0.00; deleterious	1.000; probably damaging
HADHB	c.693delC	p.A232Lfs*20	Disease causing; 1	—	—	—
HADHB	c.881C>G	p.P294R	Disease causing; 0.9999999995	-7.871; deleterious	0.00; deleterious	1.000; probably damaging

[a] Prediction and probability of the prediction, i.e. a value close to 1 indicates a high “security” of the prediction.

[b] Provean predictions are based on protein structure and function; variants with a score equal to or below -2.5 are considered “deleterious”.

[c] SIFT predictions are based on evolutionary conservation (sequence homology) and the physical properties of amino acids; amino acids with probabilities < 0.05 are predicted to be deleterious.

[d] Polyphen-2 predictions are based on evolutionary conservation (sequence homology) and protein structure and function. The HumDiv-trained Polyphen-2 model was chosen.

Table S6. Sequences of oligonucleotide primers used in genotype confirmation for exon and adjacent intronic region amplification. See also Key Resources Table.

***HADHA* Fragment**	***HADHA* Exons**	***HADHA* position cDNA**	***HADHA* Primer set**	**Size, bp**	***HADHA* sequence**
A	1+NC	1-179	Ex1-450HADHAfor	± 734	[-21M13]-AAGTGGAATCTCGCCTTTGG
A			In1MTPa rev		[M13rev]-GGAAGTGACAGTCTCTTCAG
B	2,3	68-180	In1MTPa for	± 520	[-21M13]-GCAGTAGTCATATGGCCTAG
B			In3MTPa rev		[M13rev]-CCCTTGGCAATCTGTCAAAG
C	4	181-314	In3HADHAfor	± 530	[-21M13]-AAGCTACTGACTTGCTGGGA
C			In4HADHArev		[M13rev]-GCTCTTGGCCTCATGTGATCC
D	5	315-453	In4MTPa for	± 570	[-21M13]-GGTCTCATGGATCCAGAAGT
D			In5MTPa rev		[M13rev]-GTGATGCACACCTGTAATCC
E	6	454-573	In5MTPII for	± 540	[-21M13]-GCTTCTCTAGCTTTCAGTAG
E			In6+131HADHArev		[M13rev]-GTCACTGATCCTGTACACTC
F	7	574-676	In6MTPa for3	± 490	[-21M13]-GCATAAACATATGGCTTGACTT
F			In7+232HADHArev		[M13rev]-TGGAACTACAGGTTTGCACC
G1	8	677-799	In7MTPa for	± 425	[-21M13]-CAGTCTCCAGTCTGAAATGG
G1			In8+144HADHArev		[M13rev]-GAGTTGTTACATCTCCTACC
G2	9	800-918	In8+125HADHAfor	± 593	[-21M13]-GGTAGGAGATGTAACAACTC
G2			In9MTP rev		[M13rev]-GGCAATAAGGAGGAGTGATC
H	10	919-975	In9MTPa for	± 640	[-21M13]-CATGCATCTGAGGGAGAAAG
H			In10MTPa rev		[M13rev]-CACATGCTGGTCTTGAACTC
I	11	976-1085	In10MTPa for	± 500	[-21M13]-CAGTGAGAGACAGACTTCTG
I			In11MTPa rev		[M13rev]-CTATAGGAATGACCAGAGGG
J	12	1086-1220	In11MTPa for	± 410	[-21M13]-CACCTTATAGTTGTGCCTGC
J			In12MTPa rev		[M13rev]-TGGCTTCACTACGGAGTATC
K	13	1221-1392	In12-141HADHAfor	± 392	[-21M13]-GAAAGTCTCCCTCAAAATGG
K			In13+43HADHArev		[M13rev]-TGTTCACTACACTAGGATTC
L	14	1393-1479	In13-82HADHAfor	± 380	[-21M13]-GGTGTCACTCATTAGCTTTG
L			In14MTPa rev		[M13rev]-CTTACTCCTGCATCTCACAC
M1	15	1480-1620	In14-152HADHAfor	± 490	[-21M13]-AAGACCCATGGAACCAAACC
M1			In15+164HADHArev		[M13rev]-CAGTGGGACAGTCAATACCA
M2	16	1621-1689	In15+149HADHAfor	± 580	[-21M13]-ATTGACTGTCCCACTGAGTC
M2			In16MTPa rev		[M13rev]-CAGTATAAGCCCAACTTCCG
N	17	1690-1885	In16MTPa for	± 730	[-21M13]-GTTGGTCATGACACACTCAG
N			In17+179HADHAarev		[M13rev]-AGACCATAAGTGGTTGCTAC

O	18	1886-2000	In17MTPa for	± 670	[-21M13]-CAGTGGCATAATCTCGTCTC
O			In18MTPa rev		[M13rev]-CTGGTTCTCACCTAGATGCTG
P	19,20	2001-2292	In18HADHAfor	± 570	[-21M13]-GACTTCCATTCTGCATCTGC
P			In20MTP rev		[M13rev]-TAGACACCACTCTGTTGGAG

HADHB **Fragment**	***HADHB*** **Exons**	***HADHB*** **position cDNA**	***HADHB*** **Primer set**	**Size, bp**	***HADHB*** **sequence**
NC	1	-251_-9	ex1-456HADHBfor	± 605	[-21M13]-GATCCTGAAGGCAGAAAAGC
NC			in1+121HADHBrev		[M13rev]-TCAACTCCCACTTTCCCAGG
A	2,3	1-112	HADHBbin1-173for	± 650	[-21M13]-ACCATGTTGGTGTTCCTATC
A			MTPb in3 rev		[M13rev]-CCCATTAACATCCTCAAGCC
B	4	113-212	MTPb in3 for	± 530	[-21M13]-CAGGAGAATTGCTTGAATC
B			MTPb in4 rev		[M13rev]-AAGCTATCTGGACCTCTAGG
C	5	213-257	MTPb in4 for	± 420	[-21M13]-GGAGTTAAGGCTTGCTAGAC
C			MTPb in5rev		[M13rev]-CTAAGACACTGTTAGGCCTG
D	6	258-357	In5 MTPb for	± 560	[-21M13]-GAGAAGGTGCCAAATGCTTG
D			In6 MTPb rev		[M13rev]-AGACAATGTCCTAAACCAGC
E	7	358-445	MTPb in6 for	± 510	[-21M13]-GTATCATAGCCTCGTGTCTG
E			MTPb in7 rev		[M13rev]-CTTGAGCTCAAGAGTTTGAGC
F1	8	446-633	MTPb in7 for	± 670	[-21M13]-GAAGTTGACGTCCATATGGC
F1			In8-123HDHBrev		[M13rev]-CAAGACAAGCAGAGGATCTC
F2	9	634-814	IN8FORWHADHB	± 470	[-21M13]-GCTTGTCTTGGACTTGATTGA
F2			IN9REVHADHB		[M13rev]-GGCTGAATACTTTGGAAACAG
G	10	815-936	MTPb in9 for	± 600	[-21M13]-CCTGAATCGTCTGACTTCTG
G			MTPb in10 rev		[M13rev]-GCTGGATTAGGTGACTTCTC
H	11,12	937-1064	In10HADHBfor	± 650	[-21M13]-TTTGAACAGCCTTCTCTACC
H			In12HADHBrev2		[M13rev]-ACCAATTATGAACTCAGGGC
I	13	1065-1152	MTPb in12 for	± 270	[-21M13]-CAGTTTGGGGAATATGAAGG
I			MTPb in13 rev		[M13rev]-ACTCCTGCTCTACTCACATC
J	14	1153-1227	In13-143HADHBfor	± 520	[-21M13]-TACAGATGTGAGCCACTGTG
J			MTPb in14 rev		[M13rev]-CATCTAATGTCAGAATGCAAG
K	15	1228-1392	MTPb in14 for	± 530	[-21M13]-CTTGCATTCTGACATTAGATG
K			In15HADHBrev		[M13rev]-CAGAATTACAGCTGTTATCC
L	16	1393-1428	MTPb in15 for	± 270	[-21M13]-CAGTGATTGATTGGCAGAGC
L			MTPb ex16 rev		[M13rev]-TCCTAAGAGGAGCTAGGAAC

-21M13	TGTAAAACGACGGCCAGT
M13rev	CAGGAAACAGCTATGACC

Table S7. Sequence of oligonucleotide primers and probes used in *HADHA* and *HADHB* ddPCR variant detection from cDNA and genomic DNA. See also Key Resources Table.

Oligo Name	Sequence (5′ to 3′)
Hs_HADHAc403_F	GCTATCACAAGAAGCACAGAGA
Hs_HADHAc403_R1	CATGAAATGGCAACCTCAAGTC
Hs_HADHAc403_R2	GCCACCAATGAGTTGGACAGT
Hs_HADHAc403A_pr	/56-FAM/CCACAATAG/ZEN/GCTTTGTGGACTTTTC/3IABkFQ/
Hs_HADHAc403G_pr	/56-FAM/CCACAATAG/ZEN/GCTCTGTGGACTTTTC/3IABkFQ/
Hs_HADHAc1528_F1	GACCTGAGAAGGTGATTGGCA
Hs_HADHAc1528_F2	CAGGTGATTGGCATGCACTAC
Hs_HADHAc1528_R	CTGAAGCACTGGTGTCTTTGG
Hs_HADHAc1528G_pr	/56-FAM/ATGCAGCTG/ZEN/CTGGAGATTATCACGA/3IABkFQ
Hs_HADHAc1528C_pr	/56-FAM/ATGCAGCTG/ZEN/CTGCAGATTATCACGA/3IABkFQ/
Hs_HADHBc881_F1	GTACCCTTCAAAGTACCAGGAA
Hs_HADHBc881_F2	ACCAAAGATAATGGCATCCGTC
Hs_HADHBc881_R	CAGCTGTCACTGTGCCGTAG
Hs_HADHBc881C_pr	/56-FAM/CCAAACTAA/ZEN/AACCTGCATTCATCAAG/3IABkFQ/
Hs_HADHBc881G_pr	/56-FAM/CCAAACTAA/ZEN/AACGTGCATTCATCAAG/3IABkFQ/
GAPDH_ex7_fwd	TGGCATTGCCCTCAACGACC
GAPDH_ex8_rev	TACTCCTTGGAGGCCATGTGG
GAPDH_probe	/5HEX/CTACAGCAA/ZEN/CAGGGTGGTGGAC/3IABkFQ/
Hs_RPP30_in1_fwd	TTCCTAGCGCGGGAAACTCG
Hs_RPP30_in1_rev	TGCAAATCCCTCGCCCTCGT
Hs_RPP30_in1_HEX	/5HEX/TCCTGCAAT/ZEN/GAGGGAACTGAGGC/3IABkFQ/

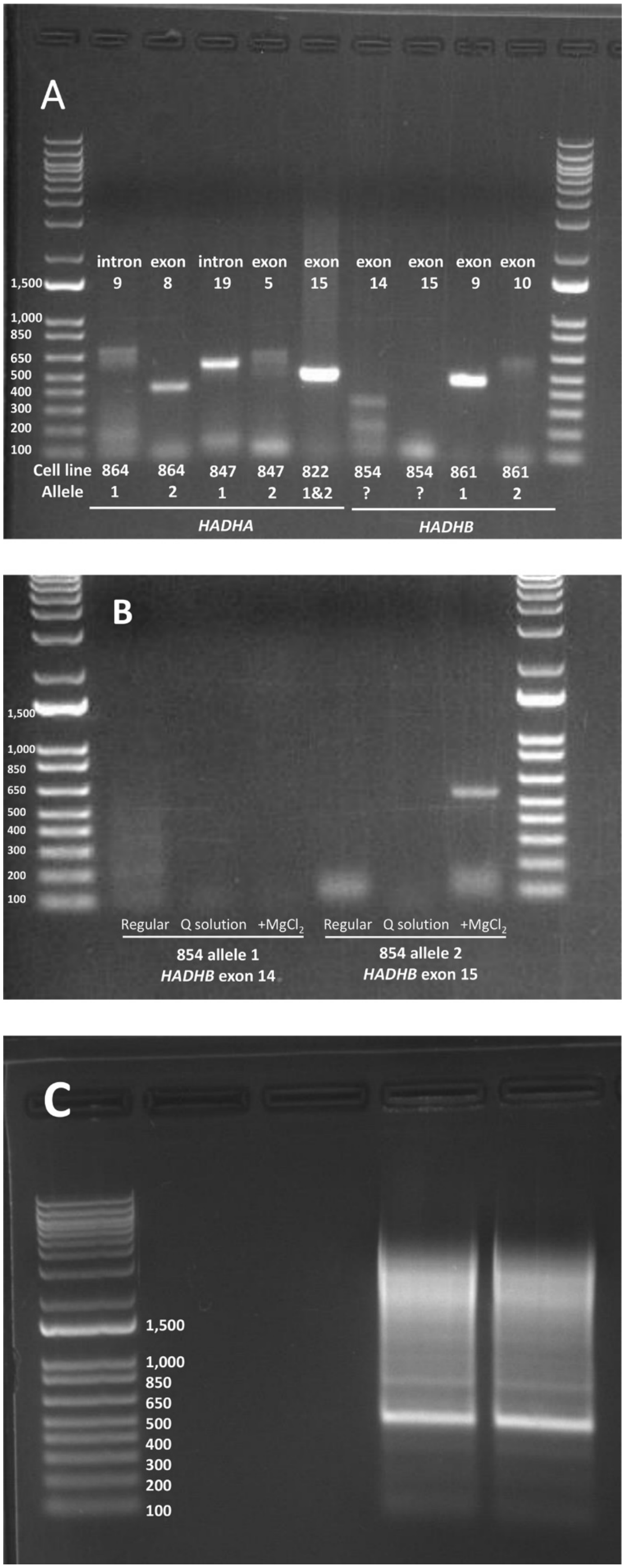

Figure S1. PCR amplification of all regions of interest for genotype confirmation. (A) PCR amplification with Promega PCR Master Mix was successful for all regions of interest, except exons 14 and 15 of HADHB for cell line 854. (B) Amplification of exon 15 of HADHB with Qiagen Taq DNA polymerase with added Q solution, and extra MgCl2. (C) Amplification of exon 14 of HADHB from cell line 854 was successful with Invitrogen AccuPrime™ High-fidelity Pfx DNA Polymerase.

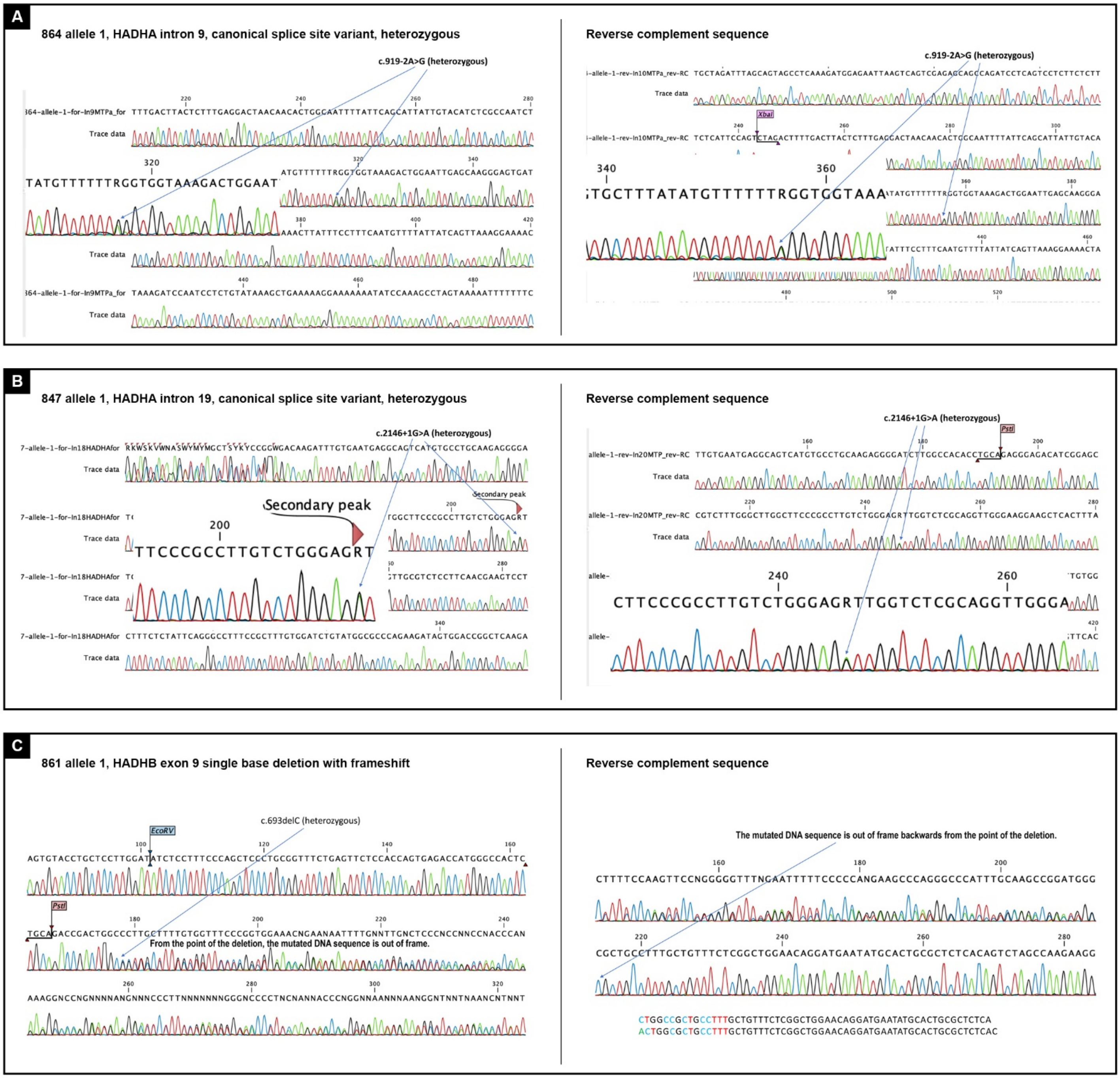

Figure S2. Sanger sequencing confirmation of three truncating variants found in patients of this study. (A) HADHA intron 9 canonical splice site variant, heterozygous; (B) HADHA intron 19 canonical splice site variant, heterozygous; (C) Single base HADHB deletion in exon 9 leading to a frameshift.

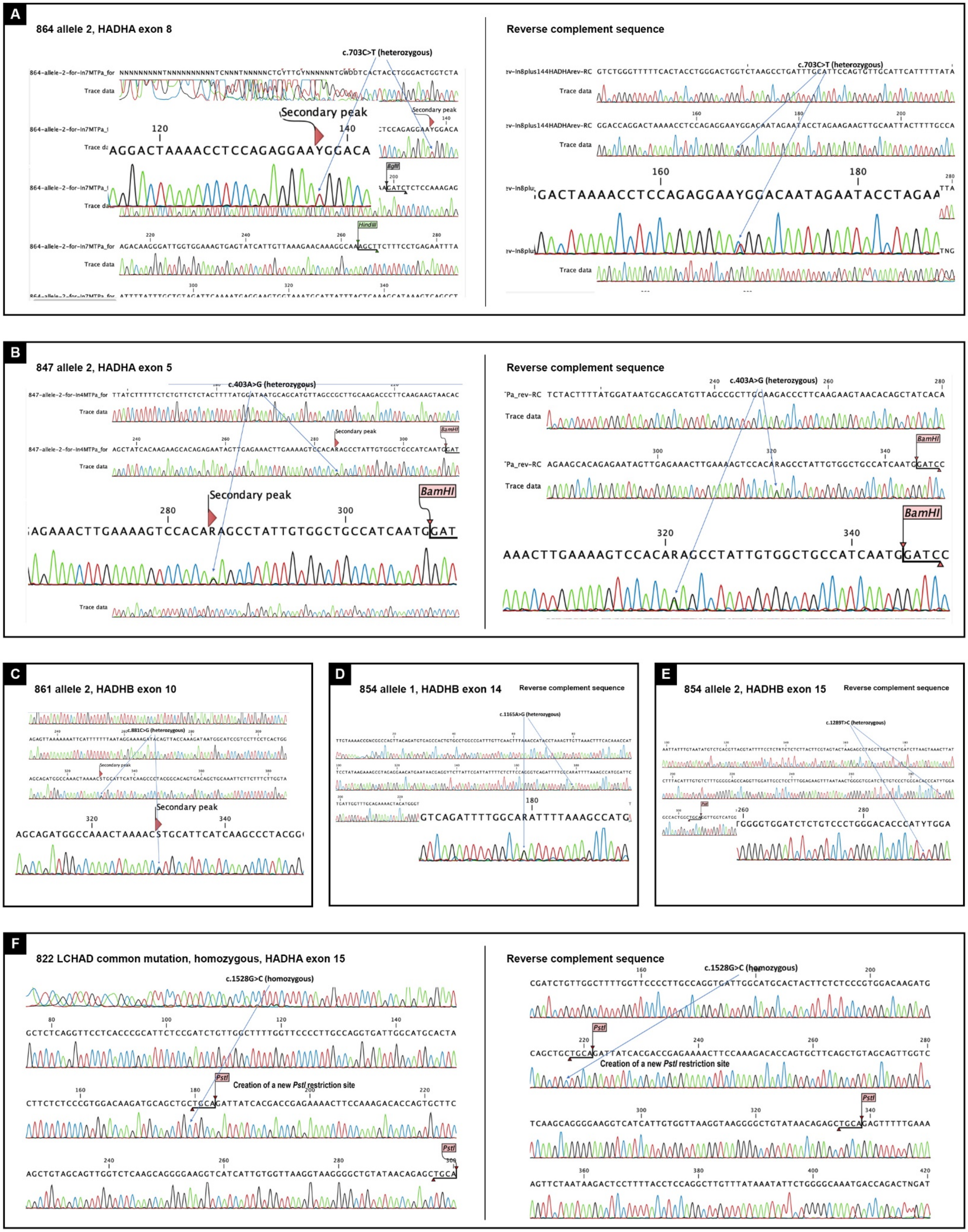

Figure S3. Sanger sequencing confirmation of missense variants found in patients of this study. (A) HADHA exon 8, c.703C>T, heterozygous; (B) HADHA exon 5, c.403A>G, heterozygous; (C) HADHB exon 10, c.881C>G, heterozygous; (D) HADHB exon 14, c.1165A>G, heterozygous; (E) HADHB exon 15, c.1289T>C, heterozygous; (F) HADHA exon 15, c.1528G>C, homozygous.

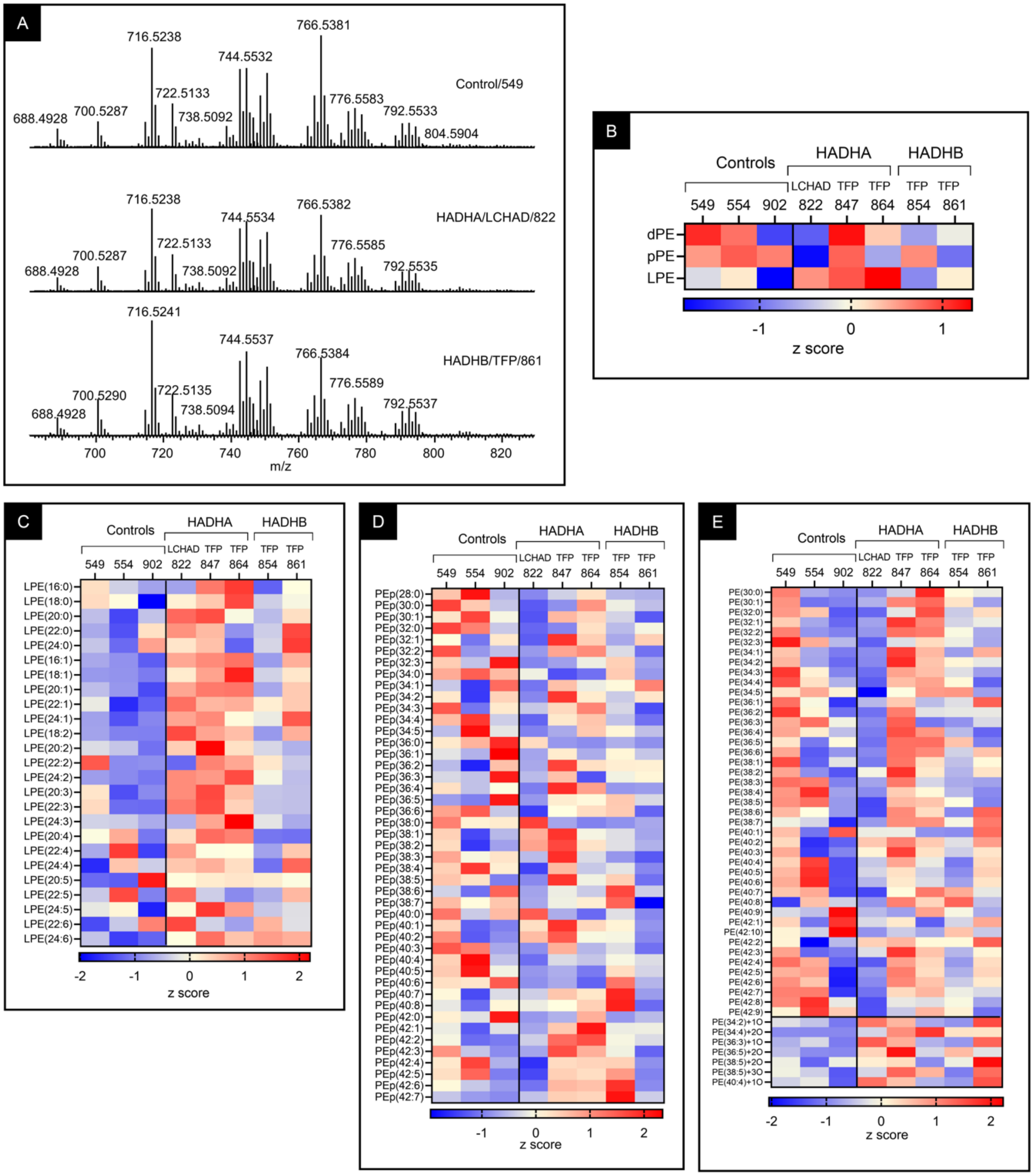

Figure S4. LC-MS/MS assessment of phosphatidylethanolamine (PE) species in human fibroblasts. Typical mass spectra of phosphatidylethanolamine (PE) obtained from control (FB549) and TFP/LCHAD-deficient fibroblasts (FB822 and FB861) (A). Diacyl-PE (dPE, PE) had significant variation among controls, hindering the interpretation of the patterns found in TFP/LCHAD-deficient fibroblasts (B&E). Lyso-PE (LPE) and oxidized PE had clearly increased levels in TFP/LCHAD-deficient fibroblasts (B,C&E). FB822 and FB861, the cells with the highest levels of oxidized PE, had the lowest levels of plasmalogen-PE (pPE) (D). Figures 10-15: Data (pmol/nmol of total phospholipids) are presented as heatmaps auto-scaled to z scores as in Figure 8. Each lane represents the mean of three technical repeats.

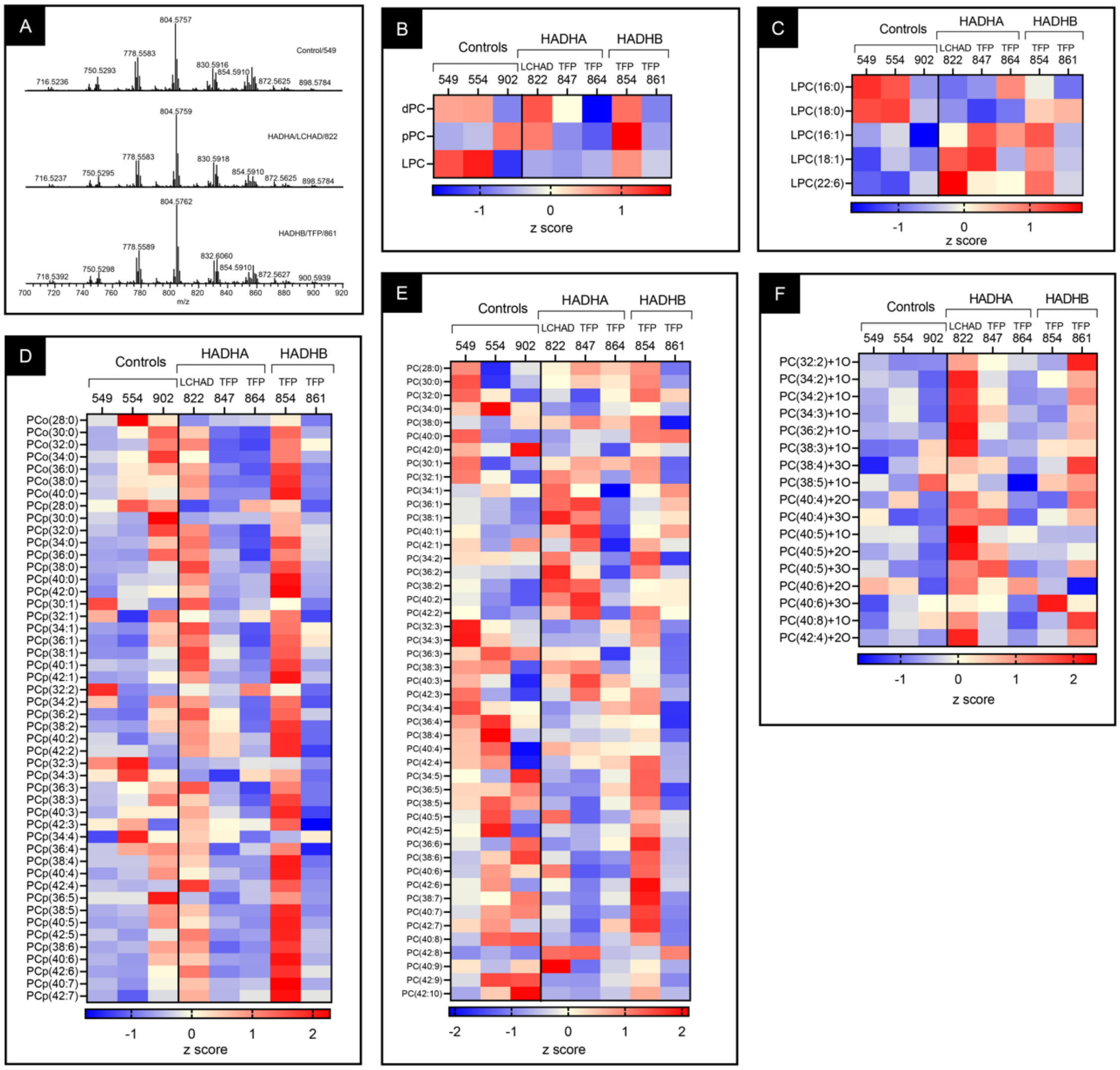

Figure S5. LC-MS/MS assessment of phosphatidylcholine (PC) species in human fibroblasts. Typical mass spectra of PC obtained from control (FB549) and TFP/LCHAD-deficient fibroblasts (FB822 and FB861). Diacyl-PC (dPC, PC), plasmalogen-PC (pPC), and lyso-PC (LPC) had significant variation among controls, hindering the interpretation of the patterns found in TFP/LCHAD-deficient fibroblasts (B-E). However, FB822 and FB854 had increased levels of PC and pPC when compared to the average of controls (B,D&E). Oxidized PC was clearly raised in FB822, FB847, and FB861 (F). Each lane represents the mean of three technical repeats.

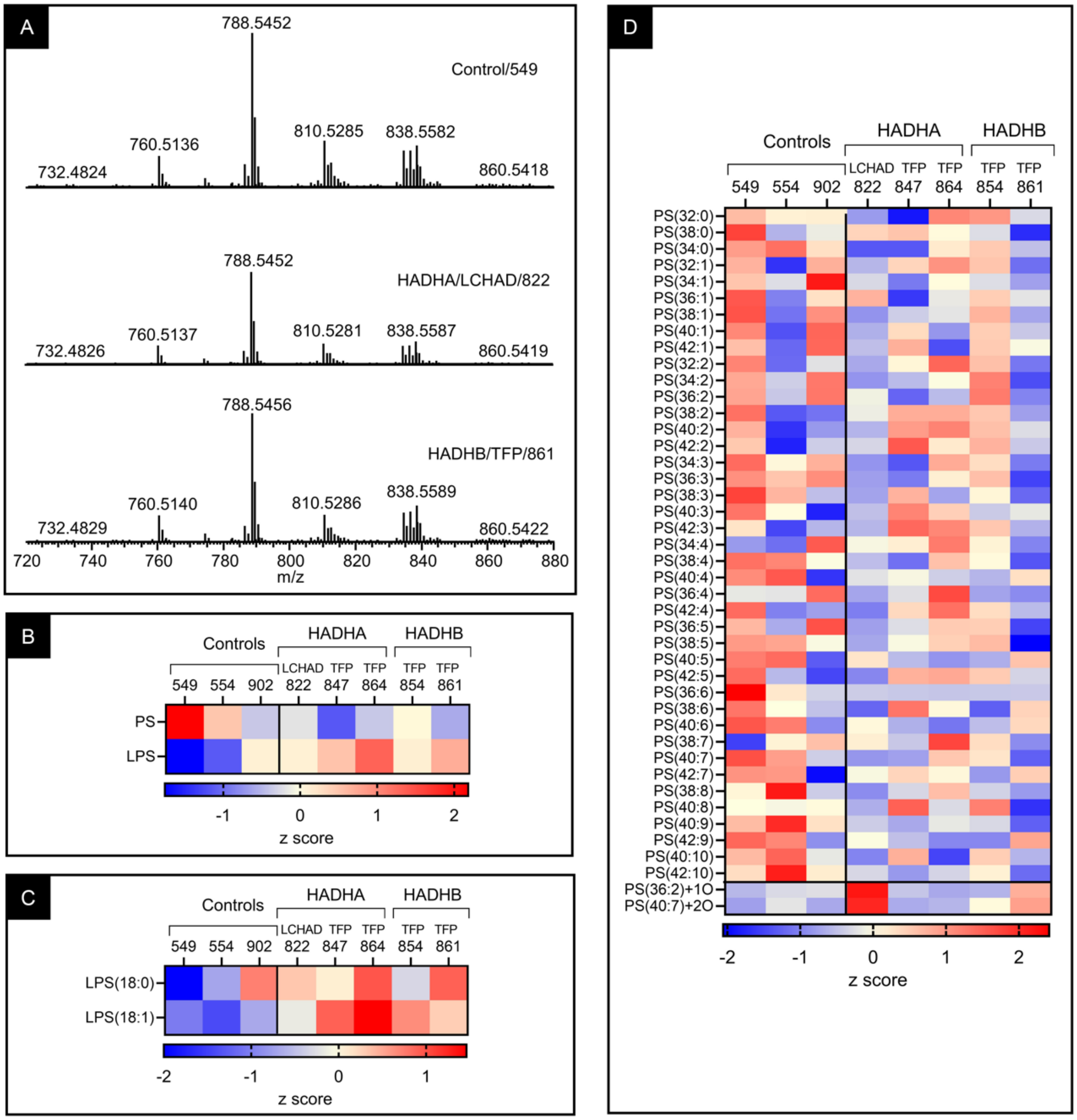

Figure S6. LC-MS/MS assessment of phosphatidylserine (PS) species in human fibroblasts. Typical mass spectra of PS obtained from control (FB549) and TFP/LCHAD-deficient fibroblasts (FB822 and FB861). Phosphatidylserine (PS) levels were decreased in all TFP/LCHAD-deficient fibroblasts, considering the average of the three controls (B&D). An opposite pattern was seen in LPS, which had increased levels in patient-derived fibroblasts compared to controls (B&C). Oxidized PS was clearly raised in FB822 and FB861 (D). Each lane represents the mean of three technical repeats.

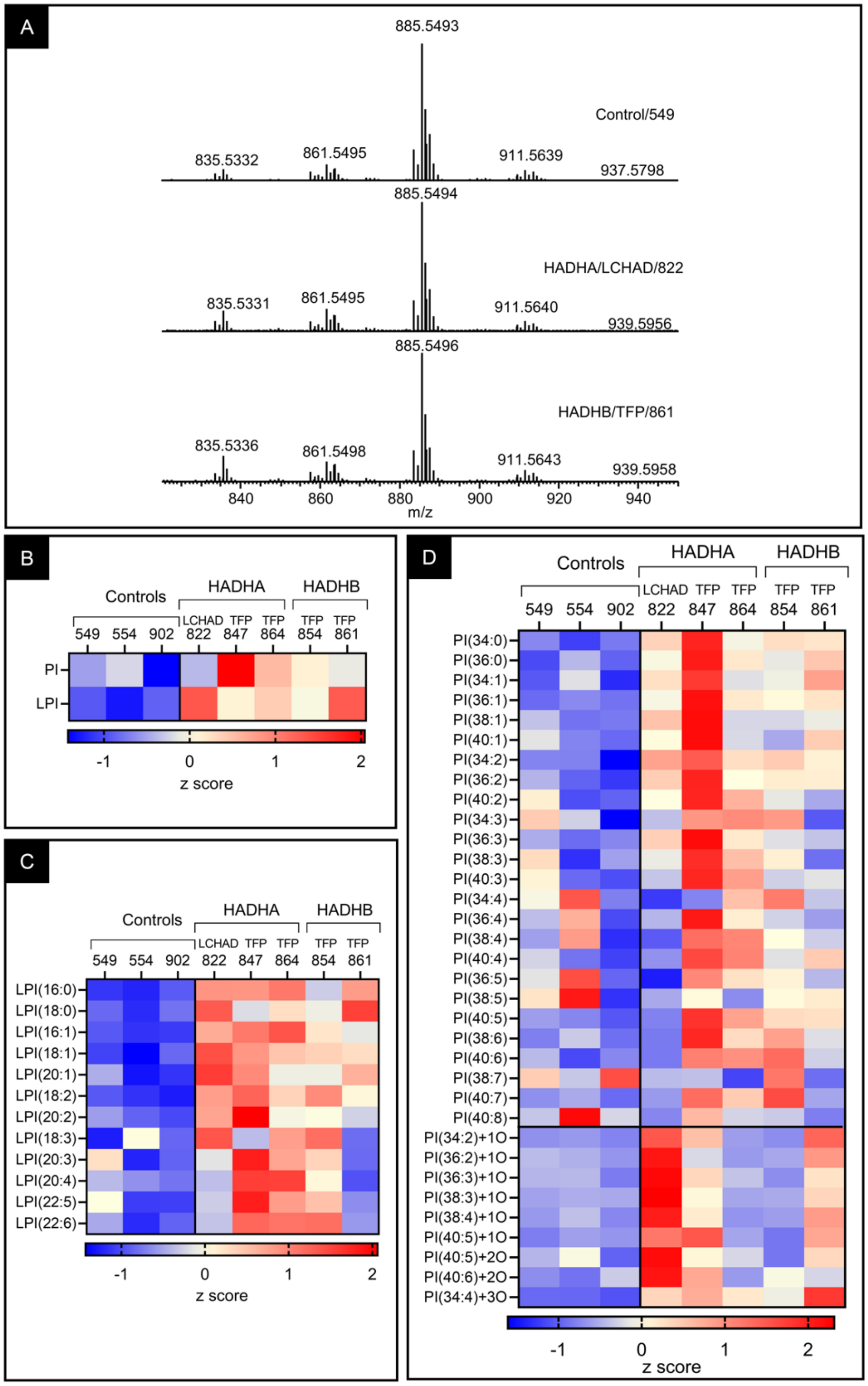

Figure S7. LC-MS/MS assessment of phosphatidylinositol (PI) species in human fibroblasts. Typical mass spectra of PI obtained from control (FB549) and TFP/LCHAD-deficient fibroblasts (FB822 and FB861). Phosphatidylinositol (PI) levels were increased in all TFP/LCHAD-deficient fibroblasts, considering the average of the three controls (B&D). The same pattern was seen in LPI, which had increased levels in patient-derived fibroblasts compared to controls (B&C). Oxidized PI was clearly raised in FB822, FB847, and FB861 (D). Each lane represents the mean of three technical repeats.

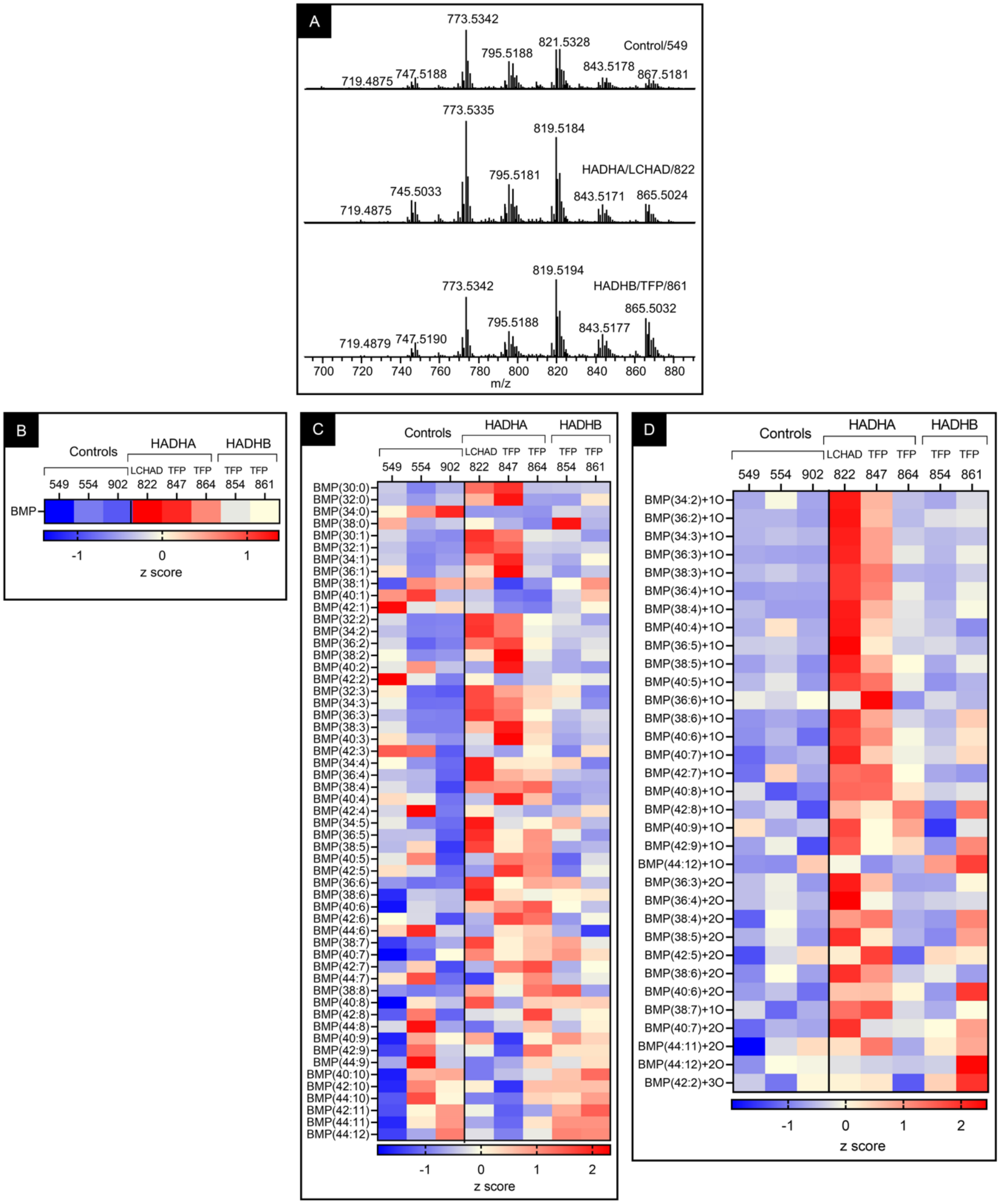

Figure S8. LC-MS/MS assessment of bis-monoacyl-glycerophosphate (BMP) species in human fibroblasts. Typical mass spectra of BMP obtained from control (FB549) and TFP/LCHAD-deficient fibroblasts (FB822 and FB861). BMP levels were increased in all TFP/LCHAD-deficient fibroblasts compared to controls (B&D). The same pattern was seen in LPI, which had increased levels in patient-derived fibroblasts compared to controls (B&C). Oxidized BMP was clearly raised in FB822, FB847, and FB861 (D). Each lane represents the mean of three technical repeats.

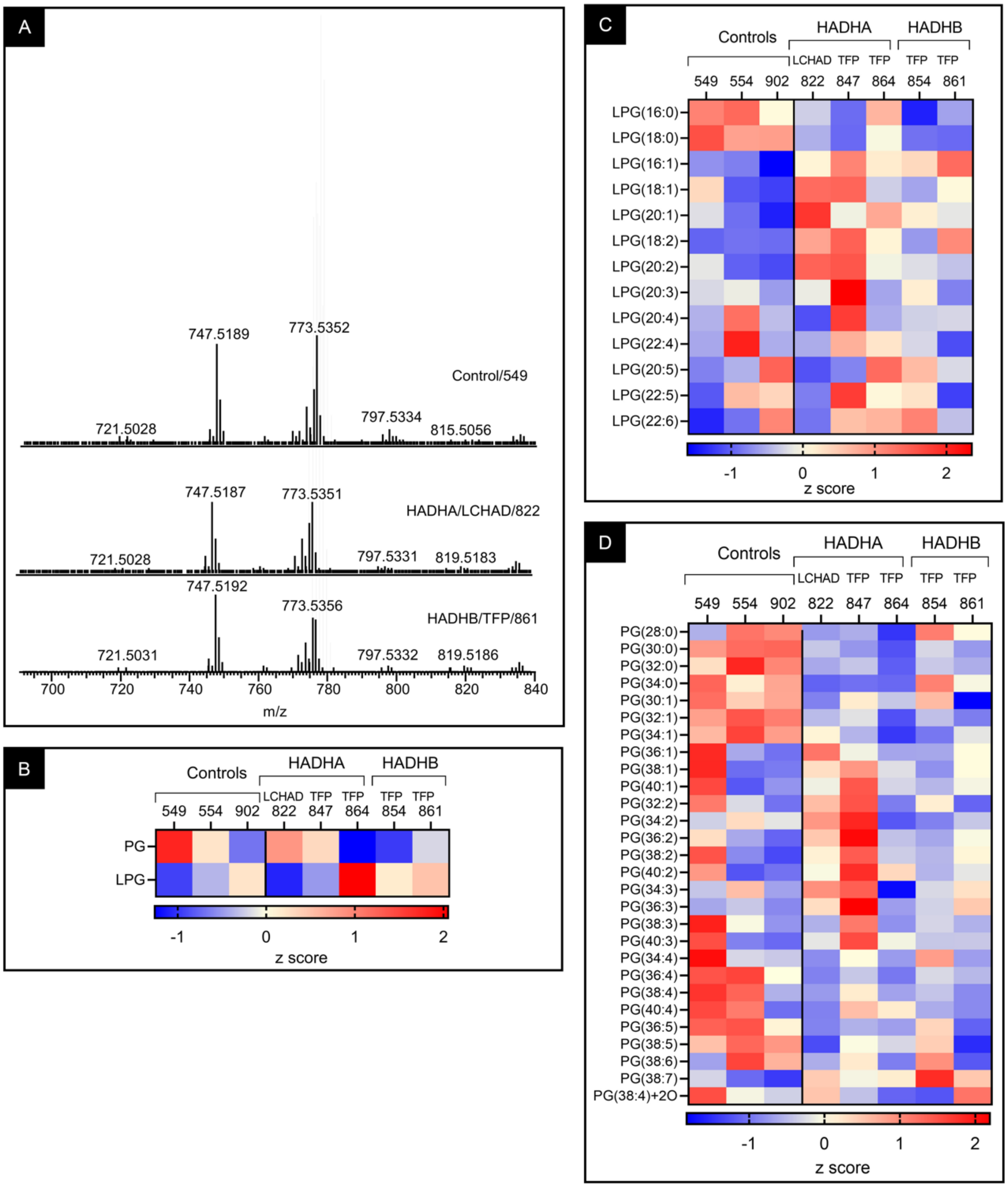

Figure S9. LC-MS/MS assessment of phosphatidylglycerol (PG) species in human fibroblasts. Typical mass spectra of PG obtained from control (FB549) and TFP/LCHAD-deficient fibroblasts (FB822 and FB861). PG and lyso-PG (LPG) levels had significant variation among controls, making it difficult to attribute the patterns found in TFP/LCHAD-deficient fibroblasts to genotype (B-D). Each lane represents the mean of three technical repeats.

Supplemental methods

Structure analysis and molecular dynamics (MD) simulations of variant proteins

Structure data of αTFP p.E510Q

Structure analysis of the variant protein αTFP p.E510Q was performed on a Silicon Graphics Fuel Workstation (Mountain View, CA, USA) using the Insight II 2000 software package (BIOVIA Dassault Systèmes, San Diego, CA, USA) using the previously published TFP atomic coordinates (6DV2.pdb) (1).

Structure data of other missense mutations

The tetrameric form of TFP, comprising of two α (HADHA) and two β (HADHB) subunits were modelled using Swiss-Model server (2). Reference sequences were retrieved from Protein [Internet], National Center for Biotechnology Information (3, 4). Crystallographic structure 6DV2 (1) was used as template. At total, two tetramers containing mutations K135E or R235W in the α subunit and three tetramers containing mutations N389D, F430S, or P294R in the β subunit were obtained. All structures were protonated at pH 7.5 using ProPKA algorithm in the PDB2PQR server (5).

Molecular dynamics (MD) simulations of missense mutations

To account for the effect of conformational changes due to mutations, the wild-type and the mutant tetrameric structures of TFP were submitted to 100 ns molecular dynamics simulations each. All MD simulations were performed using GROMACS 2020.6 (6) with the AMBER99SB forcefield (7). Each simulation system consisted of ~ 260,000 atoms that included the TFP tetramer, water molecules, and ions Na+ and Cl− at 150mM concentration. The integration steps of all simulations were set to 2 fs. Simulations were set using Particle Mesh Ewald (PME) method (8). Temperature coupling was done with V-rescale thermostat at 297.15 K, and the Parrinello-Rahman barostat (9). Water molecules were described using TIP3P water model (10). Covalent bonds were constrained to their

equilibrium length by the LINCS algorithm (11). Tetramer stability for all structures was assessed through visual inspection and root-mean-square deviation (RMSD) calculations using the “gmx rms” algorithm.

References

1. Xia C, Fu Z, Battaile KP, and Kim JP. Crystal structure of human mitochondrial trifunctional protein, a fatty acid β-oxidation metabolon. *Proc Natl Acad Sci U S A.* 2019;116(13):6069-74.
2. Biasini M, Bienert S, Waterhouse A, Arnold K, Studer G, Schmidt T, et al. SWISS-MODEL: modelling protein tertiary and quaternary structure using evolutionary information. *Nucleic Acids Res.* 2014;42(Web Server issue):W252-8.
3. Protein [Internet]. trifunctional enzyme subunit beta, mitochondrial isoform 1 precursor [Homo sapiens]. https://www.ncbi.nlm.nih.gov/protein/NP_000174.1. Updated 3/29 Accessed 6/13, 2023.
4. Protein [Internet]. trifunctional enzyme subunit alpha, mitochondrial precursor [Homo sapiens]. https://www.ncbi.nlm.nih.gov/protein/NP_000173.2. Updated 3/12 Accessed 6/13, 2023.
5. Dolinsky TJ, Czodrowski P, Li H, Nielsen JE, Jensen JH, Klebe G, et al. PDB2PQR: expanding and upgrading automated preparation of biomolecular structures for molecular simulations. *Nucleic Acids Res.* 2007;35(Web Server issue):W522-5.
6. Van Der Spoel D, Lindahl E, Hess B, Groenhof G, Mark AE, and Berendsen HJ. GROMACS: fast, flexible, and free. *J Comput Chem.* 2005;26(16):1701-18.
7. Villavicencio B, Ligabue-Braun R, and Verli H. All-Hydrocarbon Staples and Their Effect over Peptide Conformation under Different Force Fields. *J Chem Inf Model.* 2018;58(9):2015-23.
8. Essmann U, Perera L, Berkowitz ML, Darden T, Lee H, and Pedersen LG. A Smooth Particle Mesh Ewald Method. *J Chem Phys.* 1995;103(19):8577-93.
9. Parrinello M, and Rahman A. Polymorphic Transitions in Single-Crystals - a New Molecular-Dynamics Method. *J Appl Phys.* 1981;52(12):7182-90.
10. Bjelkmar P, Larsson P, Cuendet MA, Hess B, and Lindahl E. Implementation of the CHARMM Force Field in GROMACS: Analysis of Protein Stability Effects from Correction Maps, Virtual Interaction Sites, and Water Models. *J Chem Theory Comput.* 2010;6(2):459-66.
11. Hess B, Bekker H, Berendsen HJC, and Fraaije JGEM. LINCS: A linear constraint solver for molecular simulations. *Journal of Computational Chemistry.* 1997;18(12):1463-72.
